# Novel Disulfiram Derivatives as ALDH1a1-Selective Inhibitors

**DOI:** 10.3390/molecules27020480

**Published:** 2022-01-12

**Authors:** Ziad Omran

**Affiliations:** 1 Department of Pharmaceutical Sciences, Pharmacy Program, Batterjee Medical College, Jeddah 21442, Saudi Arabia; ziad.omran@bmc.edu.sa; 2 College of Pharmacy, Umm Al-Qura University, Makkah 21955, Saudi Arabia

**Keywords:** ALDH1a1, ALDH2, disulfiram

## Abstract

Aldehyde dehydrogenase-1a1 (ALDH1a1), the enzyme responsible for the oxidation of retinal into retinoic acid, represents a key therapeutic target for the treatment of debilitating disorders such as cancer, obesity, and inflammation. Drugs that can inhibit ALDH1a1 include disulfiram, an FDA-approved drug to treat chronic alcoholism. Disulfiram, by carbamylation of the catalytic cysteines, irreversibly inhibits ALDH1a1 and ALDH2. The latter is the isozyme responsible for important physiological processes such as the second stage of alcohol metabolism. Given the fact that ALDH1a1 has a larger substrate tunnel than that in ALDH2, replacing disulfiram ethyl groups with larger motifs will yield selective ALDH1a1 inhibitors. We report herein the synthesis of new inhibitors of ALDH1a1 where (hetero)aromatic rings were introduced into the structure of disulfiram. Most of the developed compounds retained the anti-ALDH1a1 activity of disulfiram; however, they were completely devoid of inhibitory activity against ALDH2.

## 1. Introduction

The aldehyde dehydrogenases (ALDHs) are a superfamily composed of 19 enzymes involved in a wide range of biological processes that are essential for cell survival and cell protection [1]. The ALDH enzymes catalyze the metabolism of both endogenous and exogenous aldehydes [2]. They are overexpressed in response to oxidative stress and lipid peroxidation [3]. They catalyze the oxidation of retinal into retinoic acid, thereby activating an important cellular differentiation pathway. Additionally, ALDHs are involved in metabolism of drugs and they have antioxidant and osmoregulatory functions [4].

Numerous studies have shown increased enzymatic activity of ALDHs, and particularly ALDH1a1, in both normal stem cells [5] and cancerous stem cells [6,7]. Overexpression of ALDH1a1 is associated with poor prognosis, tumor aggressiveness, and drug-resistance [8]. Moreover, suppressing ALDH1a1 activity depletes the stem cell pool and sensitizes stem cells to chemotherapy in different types of tissues [9]. 

ALDH1a1 is also a transcriptional regulator of metabolic responses to a high-fat diet. It is a crucial factor in adipogenesis and diet-induced obesity [10]. Additionally, mice with ALDH1a1 deficiency were shown to have decreased levels of fasting glucose, hepatic glucose production, and hepatic triacylglycerol synthesis [10,11]. Thus, ALDH1a1 inhibition reduced body weight and increased insulin sensitivity in both mice [10] and rats [12].

Moreover, it has been shown that increased production of retinoic acid by intestinal macrophages in Crohn’s disease patients is closely associated with local induction of ALDH1a1 expression, and these increased levels of retinoic acid contribute to the patient’s inflammatory phenotype [13]. Additionally, ALDH1a1-deficient mice were reported to be viable with no growth or survival defects [14]. Taken together, the available evidence supports ALDH1a1 inhibition as a promising approach to treat many diseases such as cancer, obesity, diabetes, and inflammation [15].

**Figure 1:**
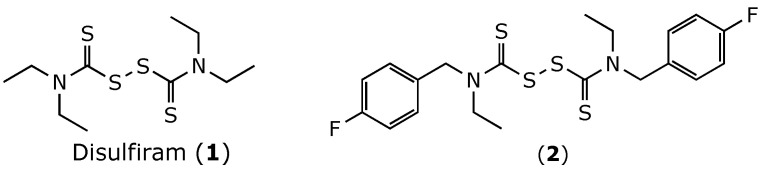
Chemical structure of disulfiram (**1**) and its analogue (**2**).

One of the most studied ALDH inhibitors is disulfiram (Figure 1), an FDA-approved drug for the treatment of chronic alcoholism. Disulfiram irreversibly inhibits ALDH2 by carbamylation of the catalytic Cys302 residue [16]. Thus, the metabolism of ethanol is blocked at the aldehyde stage, resulting in accumulation of acetaldehyde in the blood upon alcohol ingestion. This accumulation leads to unpleasant reactions such as vertigo, vomiting, tachypnea, and tachycardia, collectively known as the disulfiram reaction.

Numerous reports have pointed out the potential of repurposing disulfiram to treat cancer [17] and obesity [12]. Currently, disulfiram is progressing through several clinical trials as a treatment for different types of cancers, such as recurrent pancreatic carcinoma, metastatic breast cancer, and glioblastoma.

This study was designed to develop disulfiram analogues that can selectively inhibit ALDH1a1 without affecting ALDH2 activity. Considering the fact that ALDH2 has a smaller substrate tunnel than that of ALDH1a1 [18], bulkier disulfiram derivatives would selectively inhibit ALDH1a1. Indeed, we previously reported compound (**2**) (Figure 1), where two ethyl groups of disulfiram were replaced by *p*-fluorobenzyls [19]. Compound (**2**) showed an IC_50_ against ALDH1a1 of 0.17 *µ*M, which is comparable to that displayed by disulfiram. Nevertheless, compound (**2**) showed no inhibition for ALDH2, while disulfiram inhibited ALDH2 by an IC_50_ of 3.4 *µ*M. Thus, here we extend this work by developing new derivatives where the *p*-fluorobenzyl moiety of (**2**) was replaced by other (hetero)aromatic motifs.

## 2. Results and Discussion

**Figure 2:**
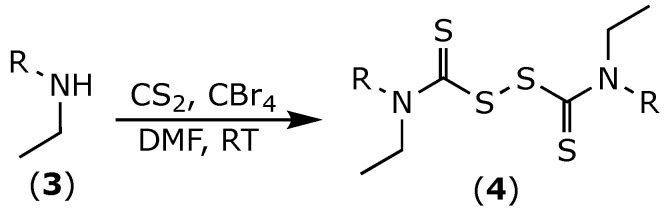
Chemical synthesis of disulfiram analogues (**4**).

Thiuram disulfides (**4**) were obtained as previously reported (Figure 2) [19,20]. Briefly, two equivalents of the corresponding secondary amine (**3**) were treated with carbon disulfide, followed by treatment with carbon tetrabromide. The desired compounds were obtained in moderate yields after classic chromatographic purification.

**Figure 3:**
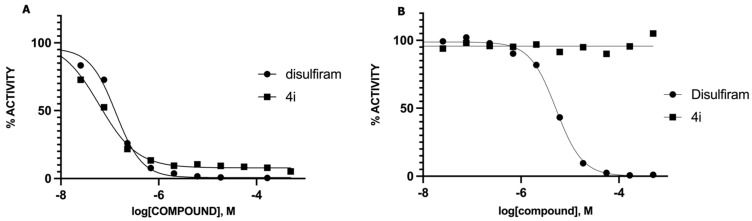
Inhibition of ALDH1a1 (**A**) and ALDH2 (**B**) by disulfiram and its synthesized analogue (**2**).

The inhibitory activity of the thiuram disulfide (**4**) was evaluated in vitro against human recombinant ALDH1a1 and ALDH2, as shown in Figure 3 and Table 1.

Disulfiram is a known and potent irreversible inhibitor of ALDH1a1. It showed an IC_50_ of 0.15 *µ*M. As reported previously, replacing two of the four ethyl groups in disulfiram with *p*-fluorobenzyl motifs was well tolerated as compound (**2**) had an of IC_50_ of 0.17 *µ*M. Changing the position of the fluoro substituent to *meta* (**4a**) or *ortho* (**4b**) slightly reduced the inhibitory activity vis à vis ALDH1a1 by 2 and 3.5 folds, respectively. Similarly, when the fluoro substituent of compound (**2**) was replaced with a moderately bigger halogen, namely a chloride, compound (**4c**) exhibited a further five-fold loss of its ALDH1a1 inhibitory activity. On the other hand, introducing a bulky group such as trifluoromethyl on the same *para* position of (**2**) resulted in the completely inactive derivative (**4d**) Aldh1a1. Conversely, introducing the electrodonating methoxy group restored the activity of compound (**4e**), as it inhibits ALDH1a1 with an IC_50_ of 0.58 *µ*M. Nevertheless, replacing the phenyl groups of (**2**) with their basic bioisostere, pyridine, reduced the inhibitory activity of compound (**4f**) by more than 33-fold. However, the five-membered ring analogues 2-furyl (**4g**) and 2-thienyl (**4h**) inhibited the enzymatic activity of ALDH1a1 by an IC_50_ of 0.39 *µ*M for both compounds. Interestingly, in compound (**4h**), where 3-thienyl was introduced, the anti-ALDH1a1 activity was significantly increased as it showed an IC_50_ of 0.17 *µ*M. This result was similar to those displayed by disulfiram and compound (**2**).

**Figure 4:**
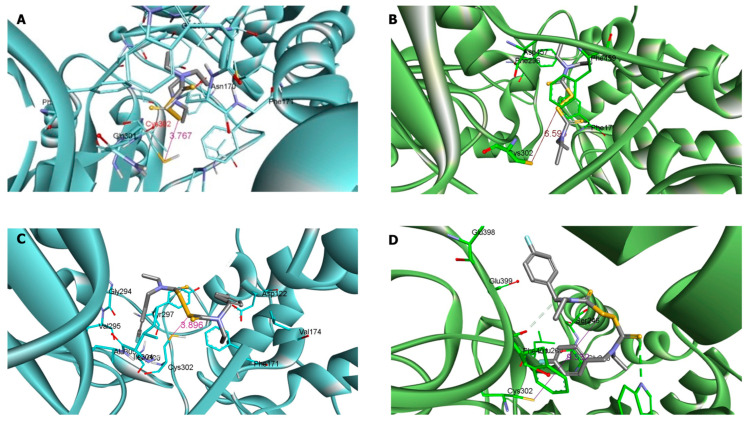
(**A**). Molecular docking of disulfiram into ALDH1a1. (**B**). Molecular docking of disulfiram into ALDH2. (**C**). Molecular docking of (**2**) into ALDH1a1. (**D**). Molecular docking of (**2**) into ALDH2.

The selectivity of the synthesized derivatives was assessed by evaluating their in vitro inhibitory activity against ALDH2. Only compound (**4g**) substituted with the relatively small furan ring showed a moderate inhibition of ALDH2, as attested by an IC_50_ of 200 *µ*M. All the other derivatives showed no ALDH2 inhibition under the assay conditions described below. This is in line with the fact that ALDH2 with its relatively small substrate tunnel cannot accommodate inhibitors with bulk substituents [18]. To further confirm these findings, disulfiram and its synthesized analogue (**2**) were docked in silico into the active sites of both ALDH1a1 and ALDH2, Figure 4. The docking results of (**2**) showed the distance between the thiuram disulfide group and thiol group of the conserved catalytic cysteine (Cys302) was significantly different in both isozymes, 3.9 Å and 9.5 Å in ALDH1a1 and ALDH2, respectively. Furthermore, the compound (**2**) exhibited additional binding interactions with back of tunnel (Phe170, Val174, and Met174) of ALDH1a1 compared to disulfiram. Moreover, (**2**) interact with Gly294, Tyr297, Glu269 and Val295 which are located at the bottom of substrate entrance tunnel. Additionally, the developed inhibitor (**2**) presented mainly binding interactions with amino acids of the surface loop (Phe170, Typ177 and Trp168) of ALDH2, and the back of the tunnel (Phe401 and Phe459). However, it does not interact with amino acids of the bottom of the substrate entrance tunnel, unlike disulfiram, which interacted with Phe296.

## 3. Materials and Methods

Solvents (EtOH, DCM, MeOH, DMF, chloroforme, toluene, and water), minerals (MgSO_4_ and CaCl_2_) were purchased from Carlo Erba Reagents (Dacit Group). All other chemicals were mainly purchased from Alfa Aesar (Karlsruhe, Germany), Merck (Sigma Aldrich) and TCI Europe (Boerenveldseweg, Belgium) or other commercial sources and used without further purification. Analytical thin layer chromatography (TLC) was performed with Nacherey Nagel (Polygram SILG/UV254) plates. Compounds were visualized by exposure to UV light or by dipping the plates in solutions of ninhydrin or potassium permanganate followed by heating. Flash column chromatography was performed with silica gel 60 (Acros Organics, Belgium). NMR spectra were recorded on BRUKER Avance III 400 MHz spectrometer in the indicated solvent. ^1^H-and ^13^CNMR chemical shifts (δ) are quoted in parts per million (ppm) relative to the TMS scale. Coupling constants *J* are quoted in Hz. The following abbreviations are used for the proton spectra multiplicities: s: singlet, d: doublet, t: triplet, q: quartet, qt: quintuplet, sp: septuplet, m: multiplet, br.: broad, dd: double doublet, dt: double triplet. Coupling constants (*J*) are reported in Hertz (Hz). Infrared spectra were run on a Shimadzu IR Affinity spectrophotometer coupled to a diamond ATR. High Resolution Mass Spectrometry (HRMS) was carried out on a Waters LCT Premier Time of Flight mass spectrometer using a Waters Acquity BEH C18 column (50 mm × 2.1 mm, 1.7 μm). HPLC separation was performed at 600 μL/min with an A/B gradient (A: water and B: CH_3_CN) using the following gradients: A (98%)/B(2%) to A (0%)/B(100%) in four minutes. This ratio was hold 1.3 min. before returning to initial conditions in 0.5 min. Mass spectra were obtained by electrospray ionization (ESI) in positive ionization mode detection. For all products, the parent ions corresponded to [M + H]^+^. The purities of all tested compounds were analyzed by HPLC, the purities being all above 95 %. Analyses were performed using a VARIAN Pro Star Module with a column X Bridge C18 (5 mm/4.6 × 50 mm) using the following isocratic eluent: Water (20 %) and CH_3_CN (80 %). Melting points were determined on a STUART Melting Point apparatus SMP20. All the spectra are provided in the Supplementary Materials.

### 3.1. Chemical Synthesis of Compounds

CS_2_ (152 mg, 2.0 mmol) was added to a solution of 4 mmol of amine (**3**) in 4 mL of DMF at 0 °C. The mixture was stirred for 5 min, after which CBr_4_ (663 mg, 2 mmol) was added. The mixture was further stirred at room temperature (RT) for 30 min. The reaction was poured into ice water (40 mL) and extracted with 2 × 40 mL CH_2_Cl_2_. The organic layer was then dried over CaCl_2_ and concentrated under vacuum. Purification by column chromatography on silica gel provided the desired products.

bis(*N*-3-Fluorobenzylethylthiocarbamoyl)disulphide (**4a**):

Column chromatography: Silica Gel, CHCl_3_. Yield: 57.4%. IR: 2978, 2932, 1614, 1589, 1481, 1439, 1412, 1346, 1250, 941, 777, 746, 681 cm^−1^. ^1^H NMR (400 MHz, CDCl_3_): 1.28–1.46 (6 H, bs), 4.02 (4 H, bs), 5.22–5.35 (4 H, bs), 6.99–7.35 (8 H, m). ^13^C NMR (100 MHz, CDCl_3_): 11.24, 13.35, 47.56, 52.35, 55.25, 59.08, 114.64, 115.11, 122.24, 130.47, 130.70, 137.21, 137.76, 161.91, 164.36, 193.81, 195.62. HR-MS (ESI^+^) *m*/*z* [M + 1] calculated 457.0712 found 457.0709.

bis(*N*-2-Fluorobenzylethylthiocarbamoyl)disulphide (**4b**):

Column chromatography: Silica Gel, CHCl_3_. Yield: 17.7%. IR: 2976, 2932, 1479, 1454, 1414, 1352, 1227, 1096, 922, 750 cm^−1^. ^1^H NMR (400 MHz, CDCl_3_): 1.29–1.47 (6 H, bs), 4.04 (4 H, bs), 5.28–5.41 (4 H, bs), 7.09–7.52 (8 H, m). ^13^C NMR (100 MHz, CDCl_3_): 11.25, 13.37, 47.79, 49.22, 52.45, 52.91, 115.32, 115.53, 121.78, 122.19, 124.72, 129.56, 159.57, 162.02, 194.12, 195.49. HR-MS (ESI^+^) *m*/*z* [M + 1] calculated 457.0712 found 457.0707.

bis(*N*-4-Chlorobenzylethylthiocarbamoyl)disulphide (**4c**):

Column chromatography: Silica Gel, CHCl_3_. Yield: 52.3%. IR: 2976, 2930, 1481, 1402, 1346, 1190, 1090, 922, 789, 750 cm^−1^. ^1^H NMR (400 MHz, CDCl_3_): 1.27–1.44 (6 H, bs), 4.00 (4 H, bs), 5.18–5.32 (4 H, bs), 7.26–7.37 (8 H, m). ^13^C NMR (100 MHz, CDCl_3_): 11.27, 13.38, 47.53, 52.27, 55.1. 58.99, 129.04, 129.09, 129.28, 133.08, 133.75, 134.24, 193.72, 195.52. HR-MS (ESI^+^) *m*/*z* [M + 1] calculated 489.0121 found 489.0128.

bis(*N*-4-trifluoromethylbenzylethylthiocarbamoyl)disulphide (**4d**):

Column chromatography: Silica Gel, CHCl_3_. Yield: 17.4%. IR: 2980, 2934, 1618, 1483, 1410, 1321, 1107, 1016, 928, 752, 650 cm^−1^. ^1^H NMR (400 MHz, CDCl_3_): 1.30–1.47 (6 H, bs), 4.04 (4 H, q), 5.28–5.41 (4 H, bs), 7.42–7.65 (8 H, m). ^13^C NMR (100 MHz, CDCl_3_): 11.28, 13.44, 47.94, 52.56, 55.23, 59.22, 122.76, 125.47, 125.87, 126.09, 127.75, 128.17, 129.96, 130.28, 138.71, 139.22, 193.89, 195.79. HR-MS (ESI^+^) *m*/*z* [M + 1] calculated 557.0648 found 557.0676.

bis(*N*-4-Methoxybenzylethylthiocarbamoyl)disulphide (**4e**):

Column chromatography: Silica Gel, CHCl_3_. Yield: 12.8%. IR: 2930, 2833, 1611, 1510, 1481, 1412, 1244, 1175, 1107,1030, 916, 789, 515 cm^−1^. ^1^H NMR (400 MHz, CDCl_3_): 1.25–1.42 (6 H, bs), 3.79 (6 H, s), 3.99 (4 H, bs), 5.16–5.31 (4 H, bs), 6.86–7.38 (8 H, m). ^13^C NMR (100 MHz, CDCl_3_): 11.18, 13.28, 46.96, 51.80, 55.38 (2C), 59.10, 114.19, 114.40, 126.48, 127.38, 129.08, 129.30, 159.36, 159.59, 193.44, 195.10. HR-MS (ESI^+^) *m*/*z* [M + 1] calculated 481.1112 found 481.1102.

bis(*N*-(4-pyridylmethyl)ethylthiocarbamoyl)disulphide (**4f**):

Column chromatography: Silica Gel, CHCl_3_-MeOH (98-2). Yield: 9.1%. IR: 2974, 2928, 1597, 1562, 1481, 1410, 1248, 1190, 930, 783, 629, 471 cm^−1^. ^1^H NMR (400 MHz, CDCl_3_): 1.21–1.43 (6 H, bs), 2.51 (0.4 H, bs), 3.99–4.04 (3.6 H, q), 5.18–5.29 (4 H, bs), 7.15–7.28 (4 H, m), 8.51–8.58 (4 H, m). ^13^C NMR (100 MHz, CDCl_3_): 11.19 (CH_3_), 13.37, 48.21, 52.79, 54.49, 58.55, 121.89, 122.00, 143.74, 144.05, 149.75, 150.09, 193.86, 195.61. HR-MS (ESI^+^) *m*/*z* [M + 1] calculated 423.0806 found 423.0813.

bis(*N*-(2-furylmethyl)ethylthiocarbamoyl)disulphide (**4g**):

Column chromatography: Silica Gel, CHCl_3_. Yield: 48.2%. IR: 3113, 2976, 2932, 1479, 1412, 1344, 1260, 1182, 1146, 1011, 935, 733, 598 cm^−1^. ^1^H NMR (400 MHz, CDCl_3_): 1.22–1.40 (6 H, bs), 4.07 (4 H, bs), 5.20–5.26 (4 H, bs), 6.34–6.51 (4 H, m), 7.38 (2 H, m). ^13^C NMR (100 MHz, CDCl_3_): 11.05, 13.19, 47.70, 49.05, 52.45, 52.61, 110.12, 110.78, 142.51, 142.97, 148.25, 148.85, 194.02, 194.44. HR-MS (ESI^+^) *m*/*z* [M + 1] calculated 401.0486 found 401.0474.

bis(*N*-(2-thienylmethyl)ethylthiocarbamoyl)disulphide (**4h**):

Column chromatography: Silica Gel, CHCl_3_. Yield: 14.2%. IR: 3069, 2980, 2928, 1531, 1483, 1412, 1341, 1260, 1155, 991, 924, 795, 725,706, 640, 511 cm^−1^. ^1^H NMR (400 MHz, CDCl_3_): 1.29–1.46 (6 H, bs), 4.03 (4 H, bs), 5.43 (4 H, bs), 6.97–7.26 (6 H, m). ^13^C NMR (100 MHz, CDCl_3_): 11.25, 13.33, 47.09, 50.84, 51.90, 54.61, 126.32, 126.44, 127.25, 127.73, 136.99, 137.45, 193.53, 194.45. HR-MS (ESI^+^) *m*/*z* [M + 1] calculated 433.0029 found 433.0027.

bis(*N*-(3-thienylmethyl)ethylthiocarbamoyl)disulphide (**4i**):

Column chromatography: Silica Gel, CHCl_3_. Yield: 12.0%. IR: 3086, 2974, 2928, 2870, 1533, 1479, 1410, 1344, 1246, 1188, 1105, 1072, 997, 974, 935, 914, 768, 704, 637, 567 cm^−1^. ^1^H NMR (400 MHz, CDCl_3_): 1.26–1.43 (6 H, bs), 4.02 (4 H, bs), 5.19–5.32 (4 H, bs), 7.13–7.37 (6 H, m). ^13^C NMR (100 MHz, CDCl_3_): 11.24 (CH_3_), 13.34, 47.31, 51.57, 52.11, 55.34, 123.44, 123.76, 126.47, 127.07, 127.25, 127.59, 127.72, 135.36, 135.90, 193.27, 194.76. HR-MS (ESI^+^) *m*/*z* [M + 1] calculated 433.0029 found 433.0048.

### 3.2. ALDH Enzyme Inhibition Assay

All tested compounds were prepared as 100 mM stock solutions in DMSO. The compounds were tested in the 10-dose IC_50_ mode, with 3-fold serial dilution at a starting concentration of 500 *μ*M. Five µL of desalted enzyme [19] (150 nM for ALDH1a1 and 200 nM for ALDH2 in reaction buffer) were added to assay wells in a Corning 384-well black plate. Five µL of reaction buffer were also added to the “no enzyme” background control wells. The test compounds were added to the assay wells. The reaction plate was then centrifuged briefly at 1200 rpm, then incubated for 20 min at RT. Five µL of substrate solution (reaction buffer containing 250 *µ*M acetaldehyde, 500 *µ*M NAD^+^ for the ALDH1a1 and 100 *µ*M acetaldehyde, 500 *µ*M NAD^+^ for the ALDH2) were then added to the assay wells. The reaction plate was briefly centrifuged and incubated at RT for 60 min. Ten µL of detection reagent composed of 15 *µ*g/mL diaphorase and 30 *µ*M were added. The reaction plate was briefly centrifuged and incubated for a further 10 min at RT in the dark. The fluorescent signal from the resorufin was measured by a Perkin Elmer Envision plate reader at Ex/Em = 535/590 nm.

### 3.3. Molecular Modelling

The crystal structure of ALDH1a1 (5L2M) and ALDH2 (5L13) [21] were downloaded from Protein Data Bank. Autodock tools (ADT) [22] was used to remove the water molecules, ligand, sulfate molecules and any unwanted residues present in the downloaded crystal structure; to add polar hydrogens; to convert the ‘clean’ crystal structure of the enzymes and saved them as ‘pdbqt’ format. Compound (**2**) and disulfiram were drawn, their energies were minimized and converted to ‘pdbqt’ format in ChemBioDraw 3D (PerkinElmer Informatics). The compounds were docked to the active site of the enzyme using PyRx [23]. The docking results generated for each of the proposed compounds were saved in ‘sdf’ format. The different conformations, or “poses,” of the docked compounds and the modes of interactions between each conformation and the amino acids in each enzyme’s tunnel were analyzed.

## 4. Conclusions

In conclusion, ALDH1a1 has important physiological roles. ALDH1a1 inhibitors are promising therapeutic agents for various disorders, including metabolic disorders, cancer, and inflammation. In this work, it was shown that disulfiram analogues in which the ethyl groups were replaced by (hetero)aromatic rings preserved the entire inhibitory activity against ALDH1a1. By contrast, with the exception of compound (**4g**), those derivatives were completely devoid of inhibitory activity against ALDH2. These promising results call for evaluating the biological comportment of these new derivatives by further in vitro and in vivo studies.

## Figures and Tables

**Table 1:**
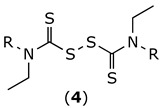
Inhibition of ALDH1a1 and ALDH2 by disulfiram and its analogues. The data are presented as the average of at least two different experiments ± the standard error. NI: no inhibition seen at a concentration of up to 0.5 mM.

Compound	R	IC_50_ (*µ*M) ± SE (ALDH1a1)	IC_50_ (*µ*M) ± SE (ALDH2)
**1** (Disulfiram)	Et	0.15 ± 0.01	3.85 ± 0.10
**2**	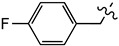	0.17 ± 0.06	NI
**4a**	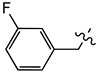	0.31 ± 0.14	NI
**4b**	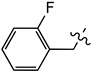	0.59 ± 0.46	NI
**4c**	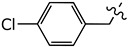	0.86 ± 0.68	NI
**4d**	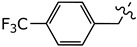	NI	NI
**4e**	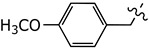	0.58 ± 0.41	NI
**4f**	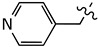	5.76 ± 4.01	NI
**4g**	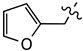	0.39 ± 0.27	200.22 ± 129.08
**4h**	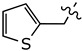	0.39 ± 0.31	NI
**4i**	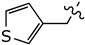	0.17 ± 0.10	NI

## Data Availability

Samples of the compounds described in this paper are available from the author.

## Supplementary Materials

The IR, ^1^HNMR, ^13^CNMR and ^1^HRMS spectra for the new compounds are included in the online supplementary material.

## Sample Availability

Samples of the compound described in this paper are available from the author.
