# Supplementary material for: Novel Disulfiram Derivatives as ALDH1a1-Selective Inhibitors
Source: Molecules. 2022 Jan 12;27(2):480. doi: 10.3390/molecules27020480 (PMC8778300; doi:10.3390/molecules27020480)
Supplement: Supplementary file 1 [file molecules-27-00480-s001.zip › molecules-1534918-supplementary.pdf]

Supplementary Materials

Novel Disulfiram Derivatives as Aldh1a1-Selective Inhibitors

Ziad Omran[1,2]

[1] Department of Pharmaceutical Sciences, Pharmacy Program, Batterjee Medical College, Jeddah 21442, Saudi Arabia; ziad.omran@bmc.edu.sa

[2] College of Pharmacy, Umm Al-Qura University, Makkah 21955, Saudi Arabia

Table of Contents

Analytical data for compound **4a**	Page 2
Analytical data for compound **4b**	Page 9
Analytical data for compound **4c**	Page 16
Analytical data for compound **4d**	Page 24
Analytical data for compound **4e**	Page 31
Analytical data for compound **4f**	Page 38
Analytical data for compound **4g**	Page 45
Analytical data for compound **4h**	Page 52
Analytical data for compound **4i**	Page 59

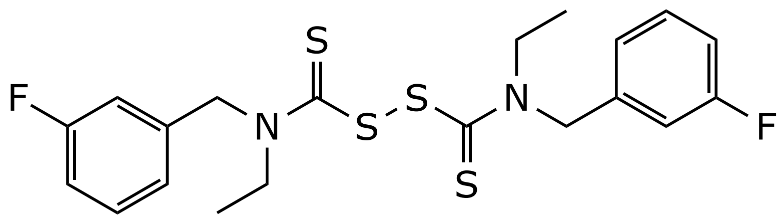

(**4a**)

Chemical Formula: $C_{20}H_{22}F_2N_2S_4$
Exact Mass: 456.0634

SHIMADZU

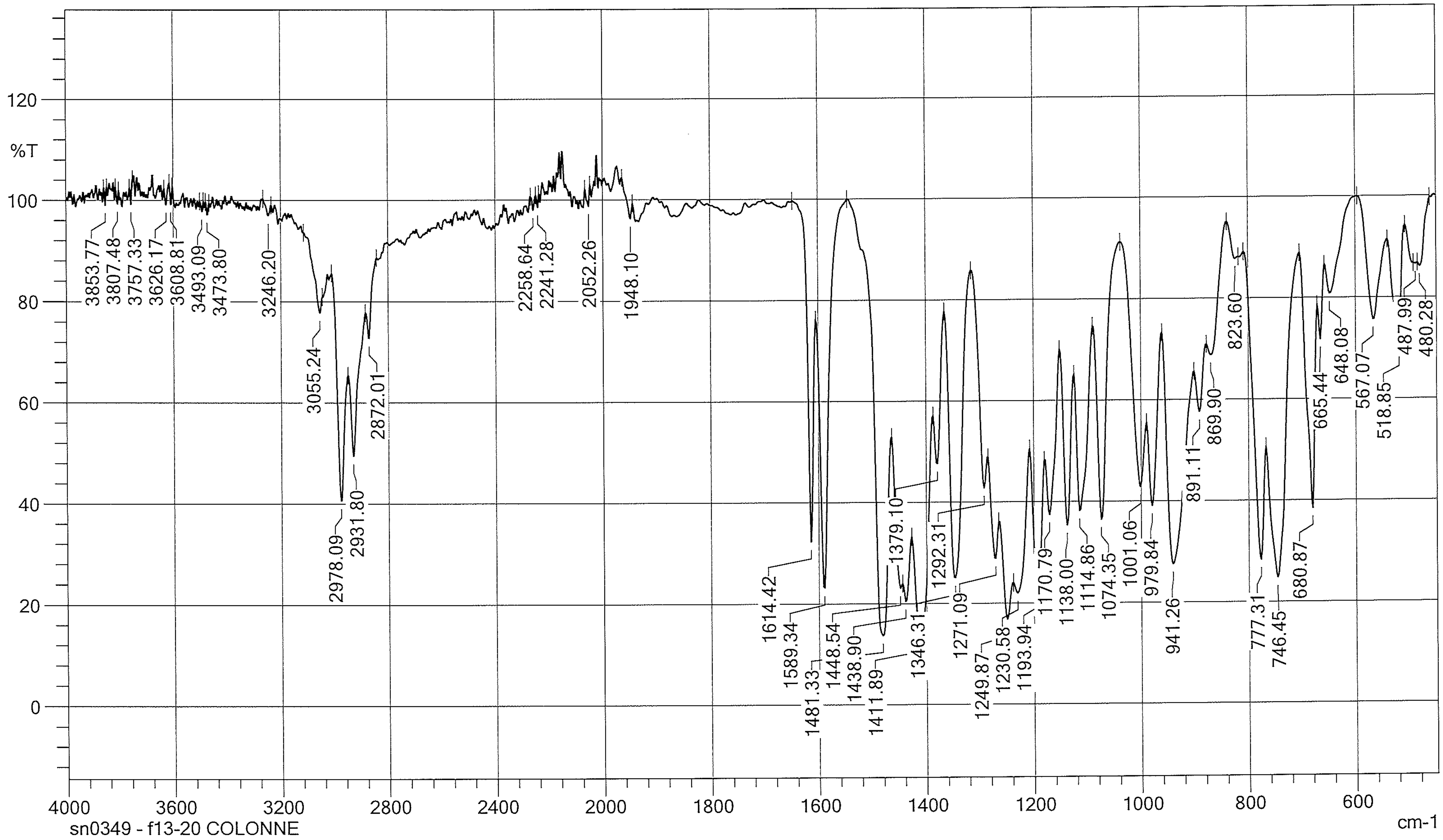

sn0349 – f13–20 COLONNE

18/05/2021 15:38:08

Elemental Composition Report

Single Mass Analysis
Tolerance = 5.0 PPM / DBE: min = 0.0, max = 14.0
Element prediction: Off
Number of isotope peaks used for i-FIT = 4

Monoisotopic Mass, Even Electron Ions
536 formula(e) evaluated with 1 results within limits (up to 50 best isotopic matches for each mass)
Elements Used:
C: 0-500 H: 0-1000 N: 0-5 F: 1-3 S: 0-6

SN0349 2:12

01-Jun-2021
2106005 567 (3.443) Cm (567:570-(535:540+596:601))

1: TOF MS ES+
1.68e+003

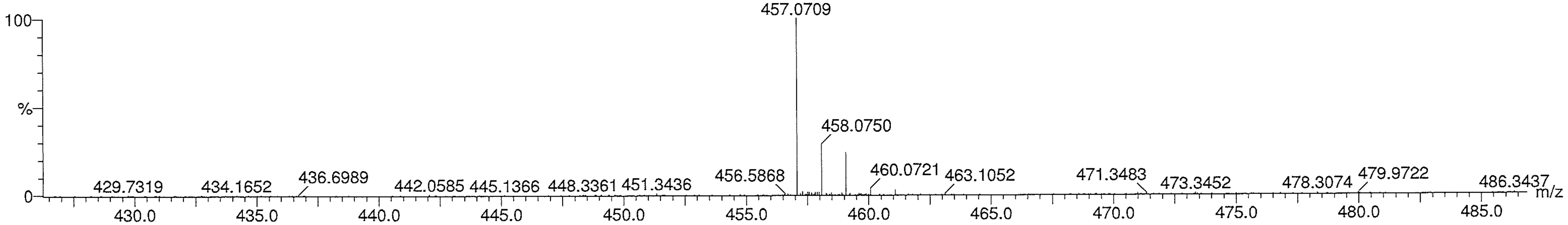

Minimum:				0.0			
Maximum:		5.0	5.0	14.0			
Mass	Calc. Mass	mDa	PPM	DBE	i-FIT	i-FIT (Norm)	Formula
457.0709	457.0712	-0.3	-0.7	9.5	153.0	0.0	C20 H23 N2 F2 S4

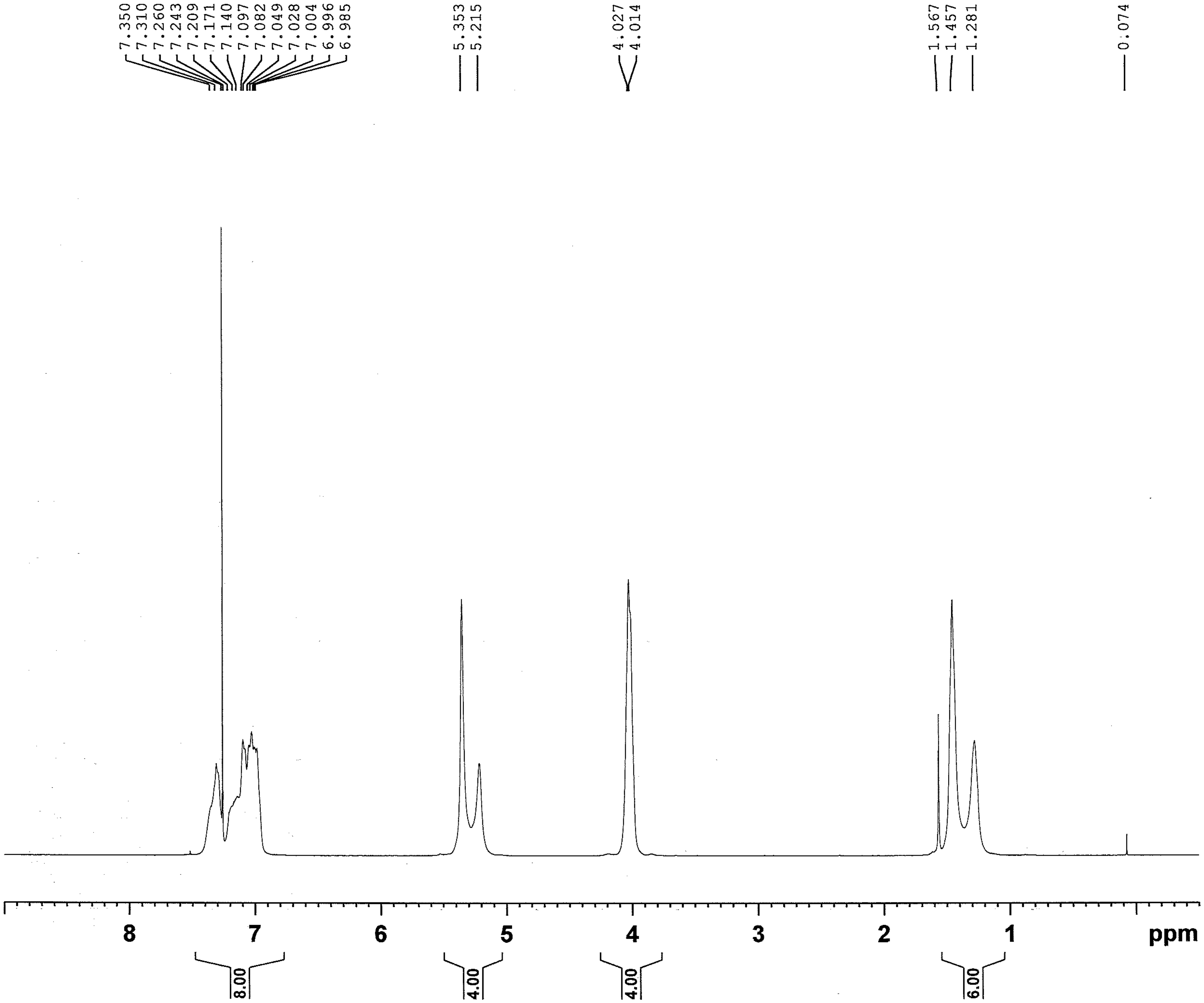

Current Data Parameters
NAME SN0349-L1
EXPNO 10
PROCNO 1

F2 - Acquisition Parameters
Date_ 20210519
Time_ 11.17
INSTRUM spect
PROBHD 5 mm PABBO BB/
PULPROG zg30
TD 32768
SOLVENT CDCl3
NS 16
DS 2
SWH 7978.724 Hz
FIDRES 0.243491 Hz
AQ 2.0534613 sec
RG 69.13
DW 62.667 usec
DE 6.50 usec
TE 296.2 K
D1 1.00000000 sec
TD0 1

======== CHANNEL f1 ========
SFO1 399.7524686 MHz
NUC1 1H
P1 9.00 usec
PLW1 19.20000076 W

F2 - Processing parameters
SI 65536
SF 399.7500096 MHz
WDW EM
SSB 0
LB 0.30 Hz
GB 0
PC 1.00

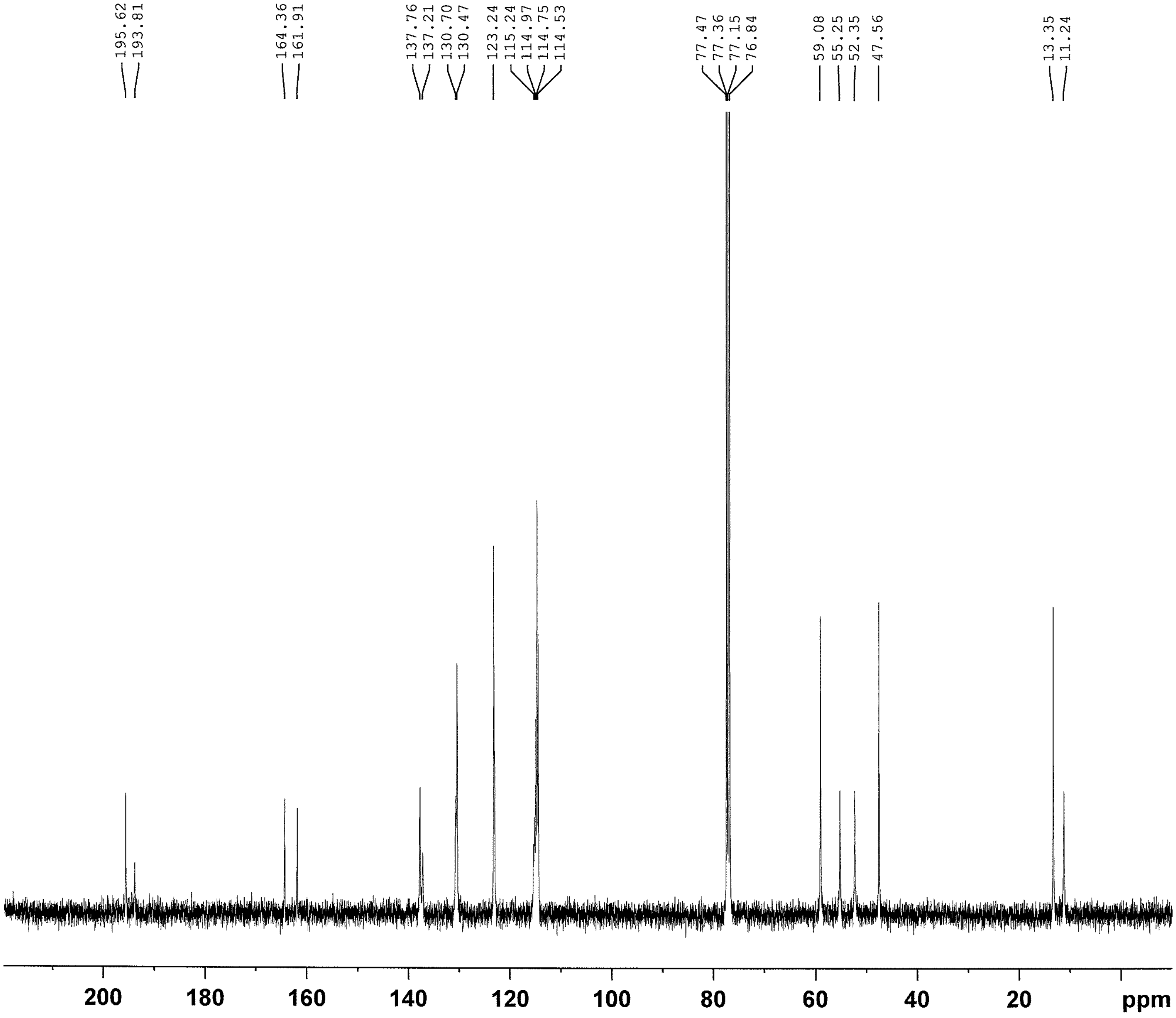


```
Current Data Parameters
NAME          SN0349-LOT1
EXPNO                  10
PROCNO                  1

F2 - Acquisition Parameters
Date_            20210520
Time                 0.01
INSTRUM             spect
PROBHD   5 mm PABBO BB/
PULPROG           zgpg30
TD                 65536
SOLVENT            CDCl3
NS                  2048
DS                     4
SWH            24038.461 Hz
FIDRES          0.366798 Hz
AQ             1.3631488 sec
RG                 199.3
DW                20.800 usec
DE                  6.50 usec
TE                 295.0 K
D1            2.00000000 sec
D11           0.03000000 sec
TD0                    1

======== CHANNEL f1 ========
SFO1         100.5272687 MHz
NUC1                 13C
P1                  8.60 usec
PLW1         87.00000000 W

======== CHANNEL f2 ========
SFO2         399.7515990 MHz
NUC2                  1H
CPDPRG[2         waltz16
PCPD2              90.00 usec
PLW2         19.20000076 W
PLW12         0.20000000 W
PLW13         0.15552001 W

F2 - Processing parameters
SI                 32768
SF           100.5172094 MHz
WDW                   EM
SSB       0
LB                  1.00 Hz
GB        0
PC                  1.40
```

Title :
Run File : C:\Users\DG\Documents\SYNTHENOVA\Mode opératoire\SN0346-49 421-26\thiuram HPLC\HPLC ok\SN0349-L1.run
Method File : C:\star\startup1.mth
Sample ID : SN0349-L1

Injection Date: 21/05/2021 14:56   Calculation Date: 21/05/2021 15:16

Operator : dG   Detector Type: 0800 (1 Volt)
Workstation: HDD   Bus Address : 81
Instrument : Instrument #1   Sample Rate : 10.00 Hz
Channel : 2 = 2 1   Run Time : 20.002 min

Chart Speed = 1.08 cm/min   Attenuation = 4057   Zero Offset = 2%
Start Time = 0.000 min   End Time = 20.002 min   Min / Tick = 1.00

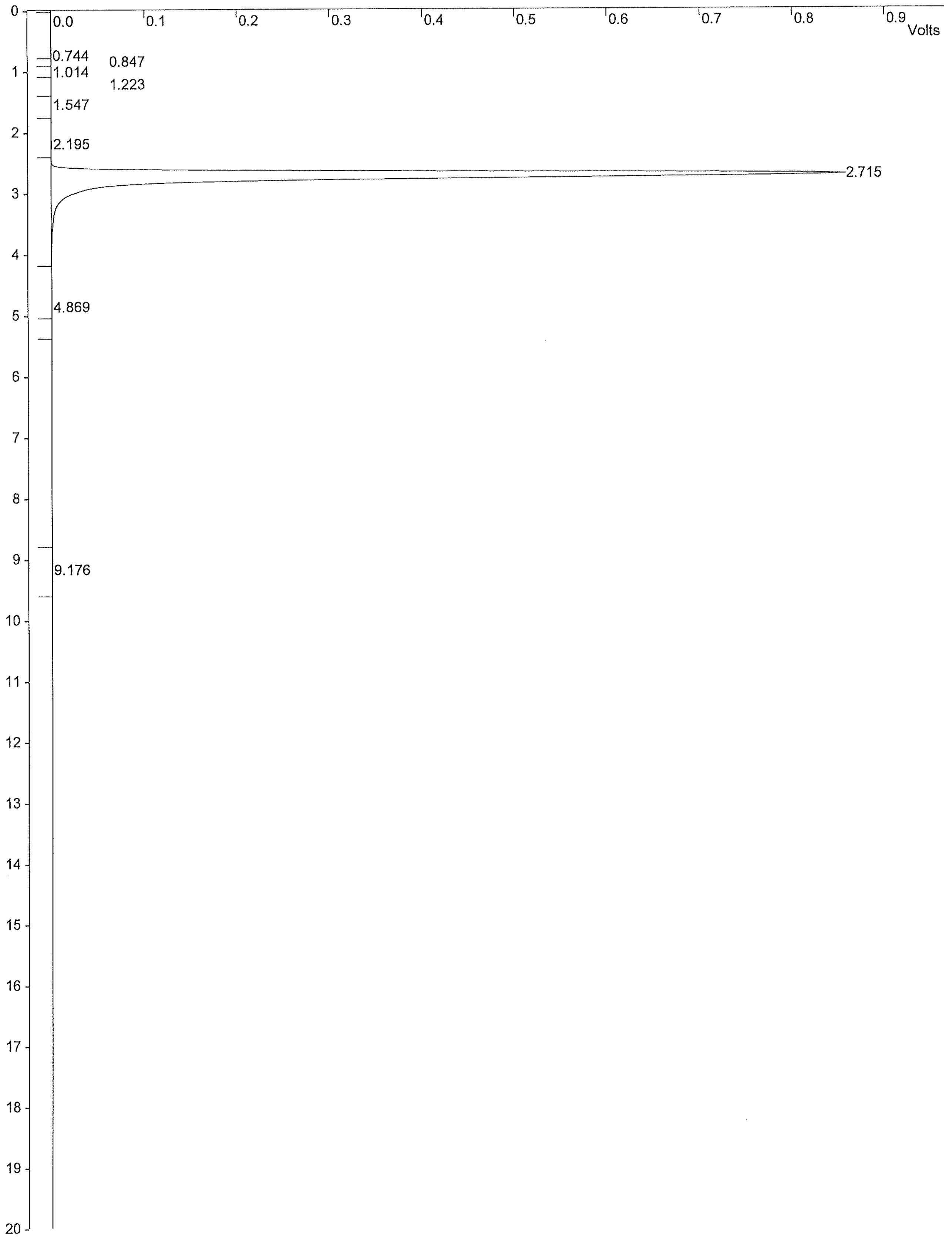

Title :
Run File : C:\Users\DG\Documents\SYNTHENOVA\Mode opératoire\SN0346-49 421-26\thiuram HPLC\HPLC ok\SN0349-L1.run
Method File : C:\star\startup1.mth
Sample ID : SN0349-L1

Injection Date: 21/05/2021 14:56 Calculation Date: 21/05/2021 15:16

Operator : dG
Workstation: HDD
Instrument : Instrument #1
Channel : 2 = 2 1

Detector Type: 0800 (1 Volt)
Bus Address : 81
Sample Rate : 10.00 Hz
Run Time : 20.002 min

Run Mode : Analysis
Peak Measurement: Peak Area
Calculation Type: Percent

Peak No.	Peak Name	Result ()	Ret. Time (min)	Time Offset (min)	Area (counts)	Sep. Code	Width 1/2 (sec)	Status Codes
1		0.0288	0.744	0.000	2116	BV	10.2	
2		0.0147	0.847	0.000	1083	VV	6.1	
3		0.0201	1.014	0.000	1480	VV	6.8	
4		0.0350	1.223	0.000	2573	VV	13.4	
5		0.0512	1.547	0.000	3761	VV	12.3	
6		0.0811	2.195	0.000	5960	VV	7.8	
7		99.6518	2.715	0.000	7321431	VP	7.2	
8		0.0470	4.869	0.000	3456	PV	0.0	
9		0.0701	9.176	0.000	5152	BB	18.9	
	Totals:	99.9998		0.000	7347012			

Total Unidentified Counts : 7347011 counts

Detected Peaks: 10 Rejected Peaks: 1 Identified Peaks: 0

Multiplier: 1 Divisor: 1 Unidentified Peak Factor: 1

Baseline Offset: -152 microVolts LSB: 1 microVolts

Noise (used): 29 microVolts - monitored before this run

Manual injection

****

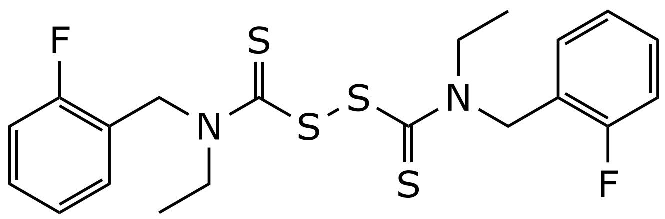

(**4b**)

Chemical Formula: $C_{20}H_{22}F_2N_2S_4$
Exact Mass: 456.0634

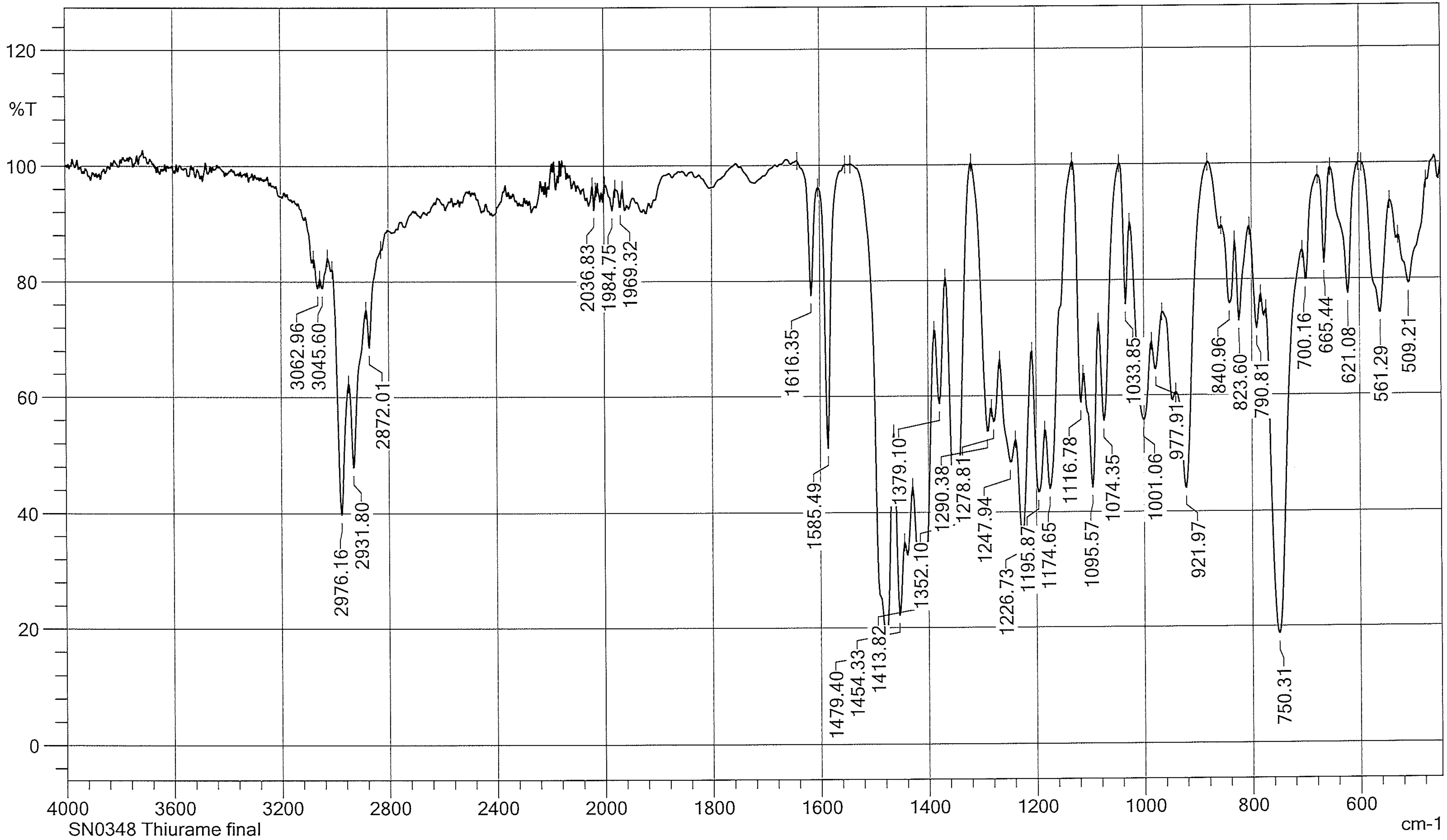

SN0348 Thiurame final

06/05/2021 12:38:39

Elemental Composition Report

Single Mass Analysis
Tolerance = 10.0 PPM / DBE: min = 0.0, max = 14.0
Element prediction: Off
Number of isotope peaks used for i-FIT = 4

Monoisotopic Mass, Even Electron Ions
536 formula(e) evaluated with 2 results within limits (up to 50 best isotopic matches for each mass)
Elements Used:
C: 0-500 H: 0-1000 N: 0-5 F: 1-3 S: 0-6

SN0348 2:11

01-Jun-2021
2106004 579 (3.501) Cm (578:581-(549:553+622:627)) 1: TOF MS ES+ 1.04e+004

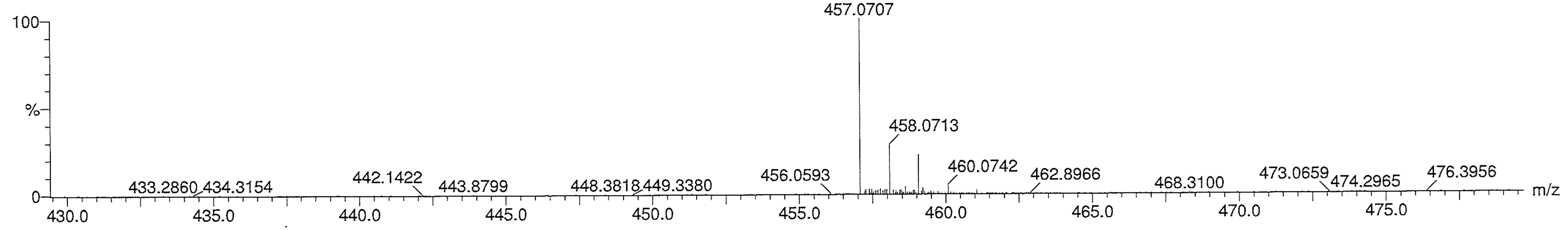

Minimum:				0.0			
Maximum:		5.0	10.0	14.0			
Mass	Calc. Mass	mDa	PPM	DBE	i-FIT	i-FIT (Norm)	Formula
457.0707	457.0712	-0.5	-1.1	9.5	434.3	0.0	C20 H23 N2 F2 S4
	457.0746	-3.9	-8.5	4.5	439.7	5.3	C17 H27 N2 F2 S5

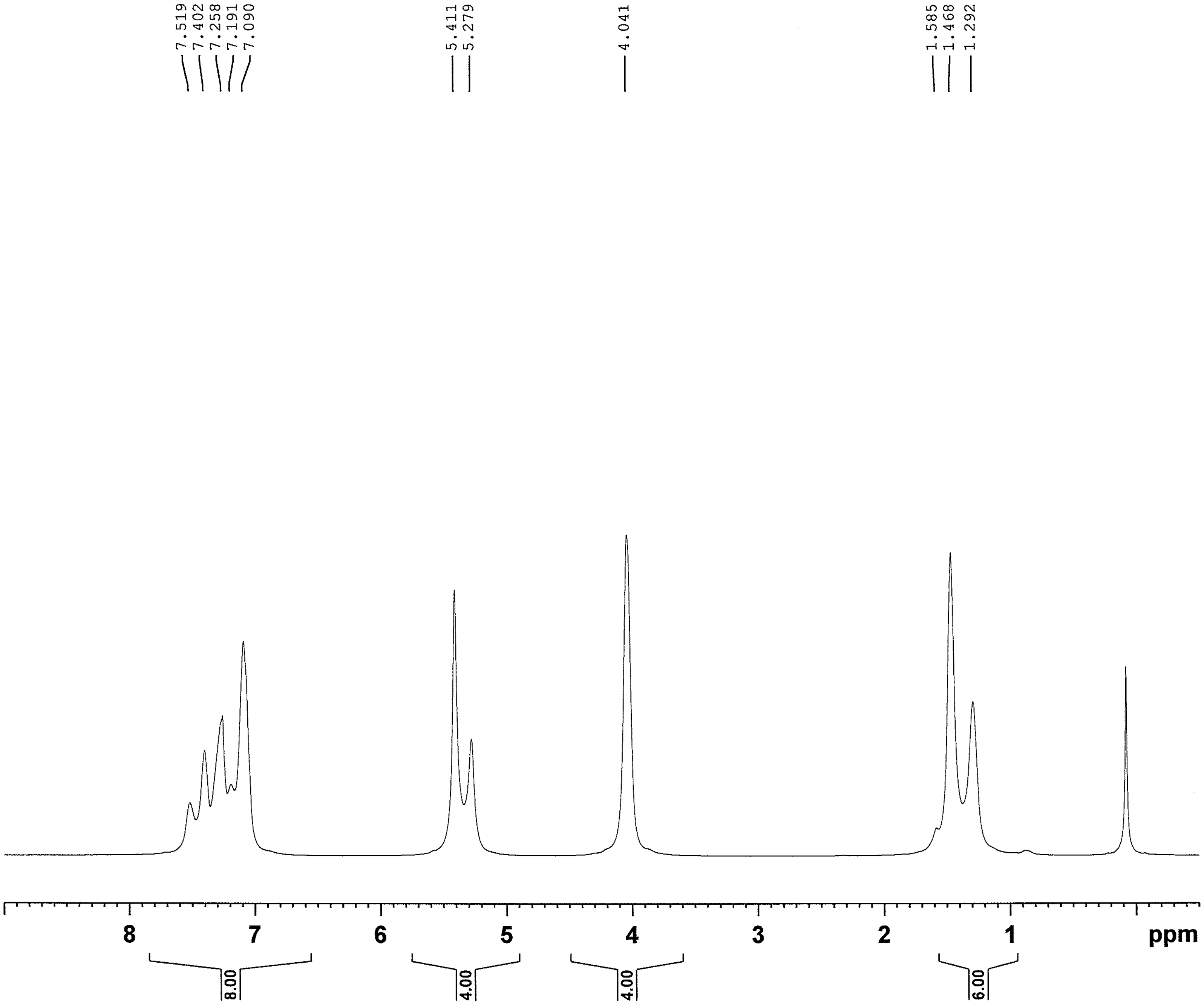


```
Current Data Parameters
NAME         SN0348-LOT1
EXPNO                 10
PROCNO                 1

F2 - Acquisition Parameters
Date_           20210506
Time               18.19
INSTRUM            spect
PROBHD   5 mm PABBO BB/
PULPROG             zg30
TD                 32768
SOLVENT            CDCl3
NS                    16
DS                     2
SWH             7978.724 Hz
FIDRES          0.243491 Hz
AQ             2.0534613 sec
RG                  30.7
DW                62.667 usec
DE                  6.50 usec
TE                 296.2 K
D1            1.00000000 sec
TD0                    1

======== CHANNEL f1 ========
SFO1         399.7524686 MHz
NUC1                  1H
P1                  9.00 usec
PLW1         19.20000076 W

F2 - Processing parameters
SI                 65536
SF           399.7500108 MHz
WDW                   EM
SSB      0
LB                  0.30 Hz
GB       0
PC                  1.00
```

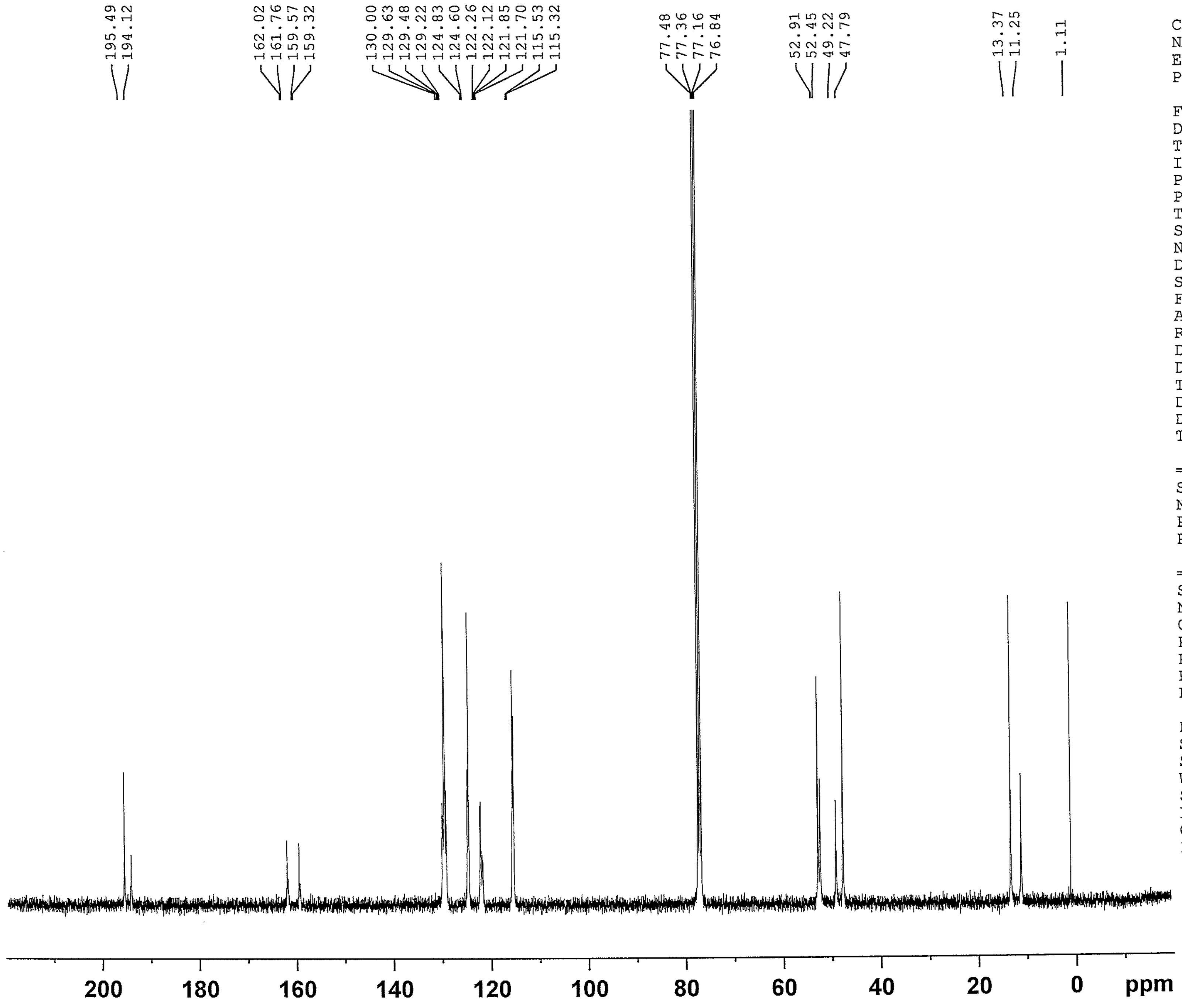

Current Data Parameters
NAME SN0348LOT1
EXPNO 10
PROCNO 1

F2 - Acquisition Parameters
Date_ 20210508
Time 15.13
INSTRUM spect
PROBHD 5 mm PABBO BB/
PULPROG zgpg30
TD 65536
SOLVENT CDCl3
NS 2048
DS 4
SWH 24038.461 Hz
FIDRES 0.366798 Hz
AQ 1.3631488 sec
RG 199.3
DW 20.800 usec
DE 6.50 usec
TE 295.0 K
D1 2.00000000 sec
D11 0.03000000 sec
TD0 1

======== CHANNEL f1 ========
SFO1 100.5272687 MHz
NUC1 13C
P1 8.60 usec
PLW1 87.00000000 W

======== CHANNEL f2 ========
SFO2 399.7515990 MHz
NUC2 1H
CPDPRG[2 waltz16
PCPD2 90.00 usec
PLW2 19.20000076 W
PLW12 0.20000000 W
PLW13 0.15552001 W

F2 - Processing parameters
SI 32768
SF 100.5172122 MHz
WDW EM
SSB 0
LB 1.00 Hz
GB 0
PC 1.40

Title :
Run File : C:\Users\DG\Documents\SYNTHENOVA\Mode opératoire\SN0346-49 421-26\thiuram HPLC\HPLC ok\SN0348-L1.run
Method File : C:\star\startup1.mth
Sample ID : SN0348-L1

Injection Date: 12/05/2021 16:02   Calculation Date: 12/05/2021 16:22

Operator : dG   Detector Type: 0800 (1 Volt)
Workstation: HDD   Bus Address : 81
Instrument : Instrument #1   Sample Rate : 10.00 Hz
Channel : 2 = 2 1   Run Time : 20.002 min

Chart Speed = 1.08 cm/min   Attenuation = 3698   Zero Offset = 2%
Start Time = 0.000 min   End Time = 20.002 min   Min / Tick = 1.00

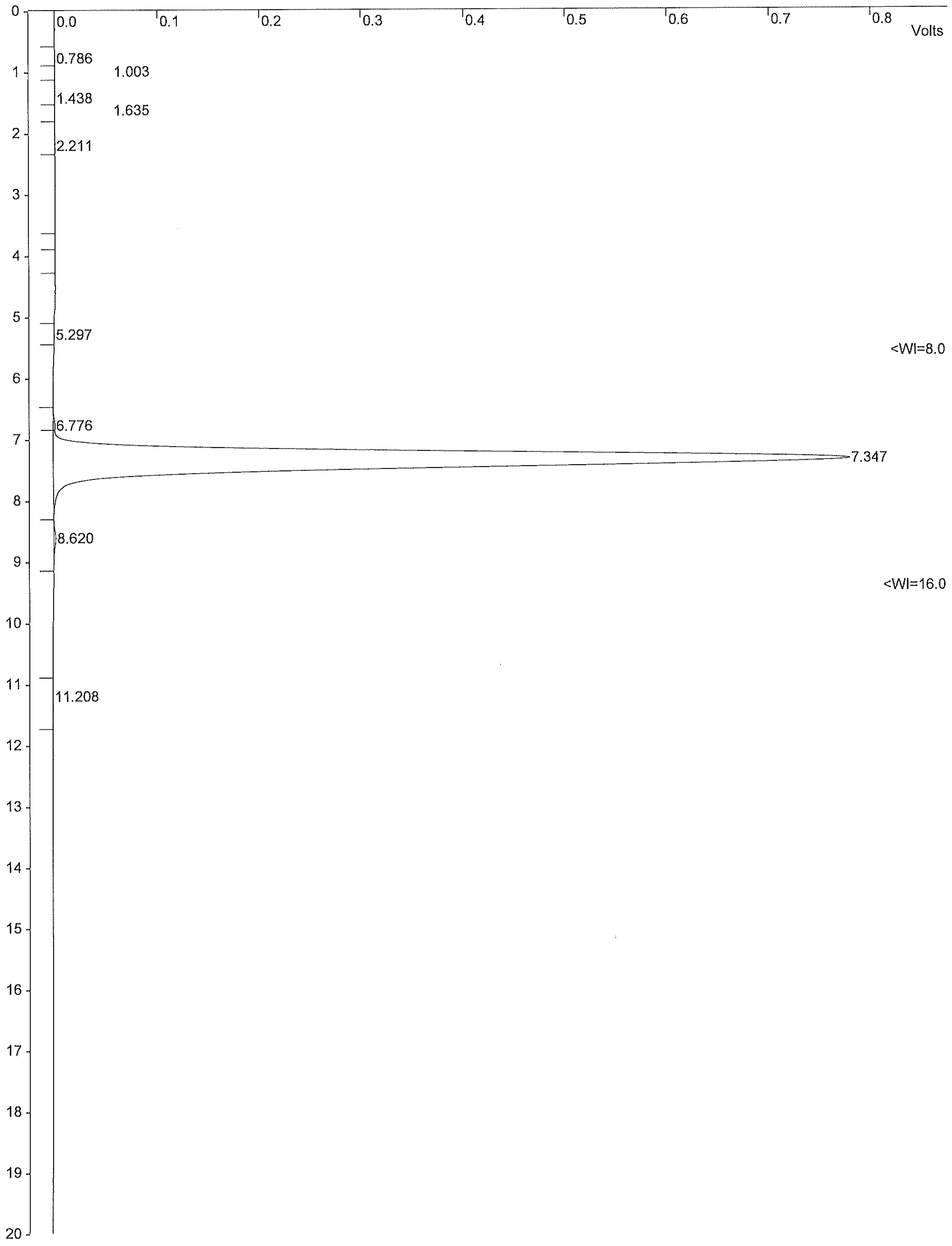

Title :
Run File : C:\Users\DG\Documents\SYNTHENOVA\Mode opératoire\SN0346-49 421-26\thiuram HPLC\HPLC ok\SN0348-L1.run
Method File : C:\star\startup1.mth
Sample ID : SN0348-L1

Injection Date: 12/05/2021 16:02 Calculation Date: 12/05/2021 16:22

Operator : dG
Workstation: HDD
Instrument : Instrument #1
Channel : 2 = 2 1

Detector Type: 0800 (1 Volt)
Bus Address : 81
Sample Rate : 10.00 Hz
Run Time : 20.002 min

Run Mode : Analysis
Peak Measurement: Peak Area
Calculation Type: Percent

Peak No.	Peak Name	Result ()	Ret. Time (min)	Time Offset (min)	Area (counts)	Sep. Code	Width 1/2 (sec)	Status Codes
1		0.0236	0.786	0.000	3277	BV	8.3	
2		0.0127	1.003	0.000	1761	VP	9.8	
3		0.0205	1.438	0.000	2838	PV	20.8	
4		0.0123	1.635	0.000	1713	VV	15.1	
5		0.0096	2.211	0.000	1332	VB	29.0	
6		0.0232	5.297	0.000	3214	BB	7.9	
7		0.1443	6.776	0.000	20032	BV	14.6	
8		99.4616	7.347	0.000	13805326	VB	16.1	
9		0.2707	8.620	0.000	37575	TS	0.0	
10		0.0216	11.208	0.000	2993	BB	19.8	
	Totals:	100.0001		0.000	13880061			

Total Unidentified Counts : 13880062 counts

Detected Peaks: 12 Rejected Peaks: 2 Identified Peaks: 0

Multiplier: 1 Divisor: 1 Unidentified Peak Factor: 1

Baseline Offset: -24 microVolts LSB: 1 microVolts

Noise (used): 31 microVolts - monitored before this run

Manual injection

****

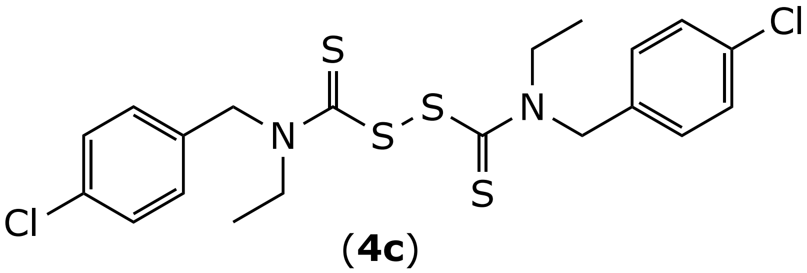

(**4c**)

Chemical Formula: $C_{20}H_{22}Cl_2N_2S_4$
Exact Mass: 488.0043

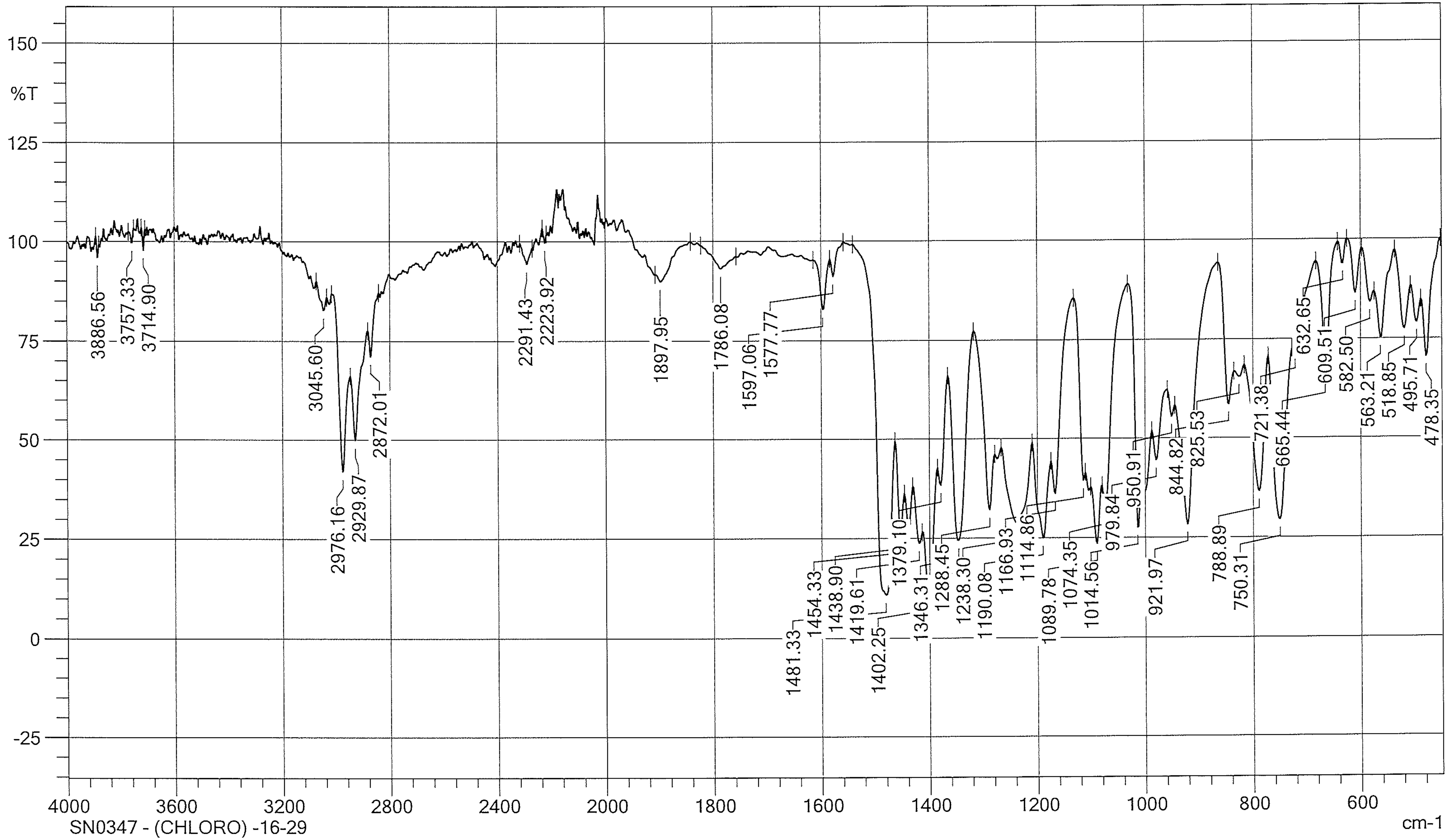

SN0347 – (CHLORO) –16–29

18/05/2021 19:11:34

Elemental Composition Report

Multiple Mass Analysis: 3 mass(es) processed

Tolerance = 10.0 PPM / DBE: min = 0.0, max = 14.0
Element prediction: Off
Number of isotope peaks used for i-FIT = 4

Monoisotopic Mass, Even Electron Ions
4839 formula(e) evaluated with 27 results within limits (up to 50 best isotopic matches for each mass)
Elements Used:
C: 0-500 H: 0-1000 N: 0-5 S: 0-6 35Cl: 0-2 37Cl: 0-2

SN0347 2:10

01-Jun-2021
2106003 619 (3.767) Cm (617:622-(576:598+677:700)) 1: TOF MS ES+ 6.65e+003

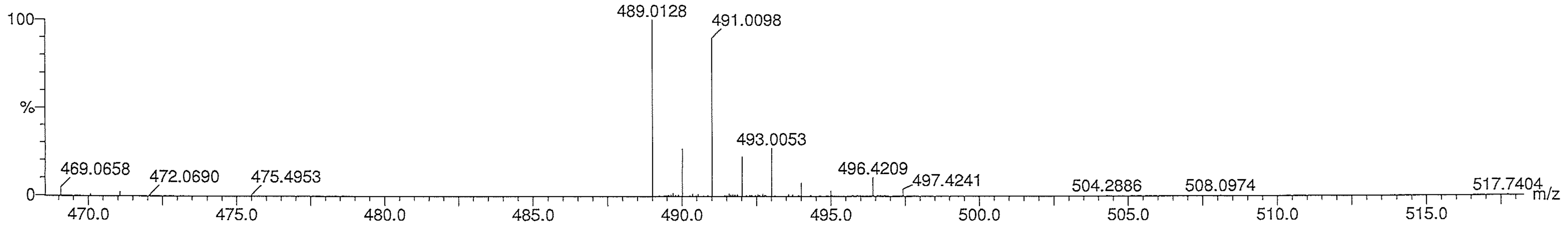

Mass	RA	Calc. Mass	mDa	PPM	DBE	i-FIT	i-FIT (Norm)	Formula
Minimum:	26.70				0.0			
Maximum:	100.00		5.0	10.0	14.0			
489.0128	100.00	489.0137	-0.9	-1.8	9.5	167.6	1.1	C18 H22 N4 S5 35Cl
		489.0121	0.7	1.4	9.5	167.6	1.1	C20 H23 N2 S4 35Cl2
		489.0155	-2.7	-5.5	4.5	168.4	1.9	C17 H27 N2 S5 35Cl2
		489.0170	-4.2	-8.6	4.5	169.3	2.7	C15 H26 N4 S6 35Cl
		489.0136	-0.8	-1.6	11.5	169.6	3.0	C20 H20 N4 S2 35Cl 37Cl2
		489.0154	-2.6	-5.3	6.5	170.1	3.6	C19 H25 N2 S2 35Cl2 37Cl2
		489.0170	-4.2	-8.6	6.5	170.3	3.8	C17 H24 N4 S3 35Cl 37Cl2
		489.0121	0.7	1.4	11.5	172.8	6.3	C22 H21 N2 S 35Cl2 37Cl2
491.0098	89.42	491.0141	-4.3	-8.8	4.5	124.7	0.3	C15 H26 N4 S6 37Cl
		491.0107	-0.9	-1.8	9.5	126.3	1.9	C18 H22 N4 S5 37Cl
		491.0125	-2.7	-5.5	4.5	127.2	2.8	C17 H27 N2 S5 35Cl 37Cl
		491.0124	-2.6	-5.3	12.5	127.3	2.9	C23 H23 S6
		491.0060	3.8	7.7	4.5	128.5	4.1	C15 H25 N4 S5 35Cl2
		491.0110	-1.2	-2.4	4.5	129.5	5.1	C19 H28 S4 35Cl2 37Cl
		491.0092	0.6	1.2	9.5	129.5	5.1	C20 H23 N2 S4 35Cl 37Cl
		491.0093	0.5	1.0	1.5	131.6	7.2	C14 H27 N4 S3 35Cl2 37Cl2
		491.0076	2.2	4.5	9.5	132.6	8.2	C22 H24 S3 35Cl2 37Cl
		491.0059	3.9	7.9	6.5	135.0	10.6	C17 H23 N4 S2 35Cl2 37Cl2
493.0053	26.71	493.0047	0.6	1.2	9.5	64.6	0.5	C22 H24 S3 35Cl 37Cl2
		493.0062	-0.9	-1.8	9.5	65.8	1.7	C20 H23 N2 S4 37Cl2
		493.0080	-2.7	-5.5	4.5	66.7	2.6	C19 H28 S4 35Cl 37Cl2
		493.0047	0.6	1.2	7.5	67.3	3.2	C20 H26 S6 35Cl
		493.0030	2.3	4.7	4.5	67.7	3.6	C15 H25 N4 S5 35Cl 37Cl
		493.0015	3.8	7.7	4.5	68.1	4.0	C17 H26 N2 S4 35Cl2 37Cl
		493.0029	2.4	4.9	12.5	68.6	4.5	C21 H21 N2 S6

Elemental Composition Report

Multiple Mass Analysis: 3 mass(es) processed
Tolerance = 10.0 PPM / DBE: min = 0.0, max = 14.0
Element prediction: Off
Number of isotope peaks used for i-FIT = 4

Monoisotopic Mass, Even Electron Ions
4839 formula(e) evaluated with 27 results within limits (up to 50 best isotopic matches for each mass)
Elements Used:
C: 0-500 H: 0-1000 N: 0-5 S: 0-6 35Cl: 0-2 37Cl: 0-2

SN0347 2:10

01-Jun-2021
2106003 619 (3.767) Cm (617:622-(576:598+677:700)) 1: TOF MS ES+ 6.65e+003

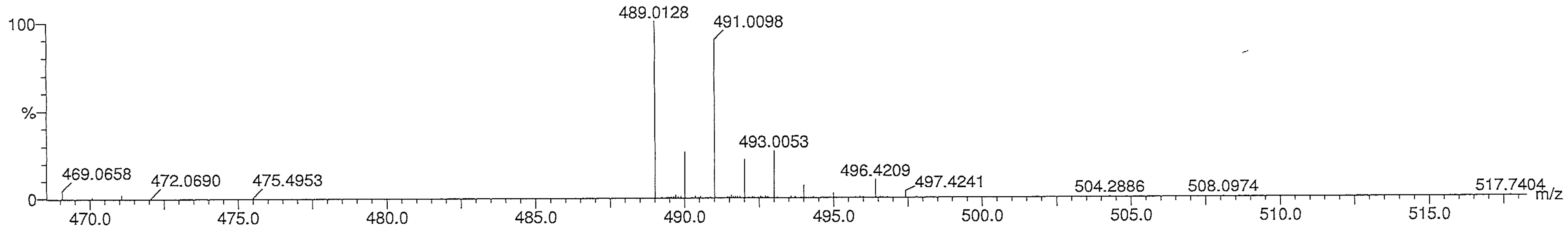

Mass	RA	Calc. Mass	mDa	PPM	DBE	i-FIT	i-FIT (Norm)	Formula
Minimum:	26.70				0.0			
Maximum:	100.00		5.0	10.0	14.0			
		493.0014	3.9	7.9	12.5	69.5	5.4	C23 H22 S5 35Cl

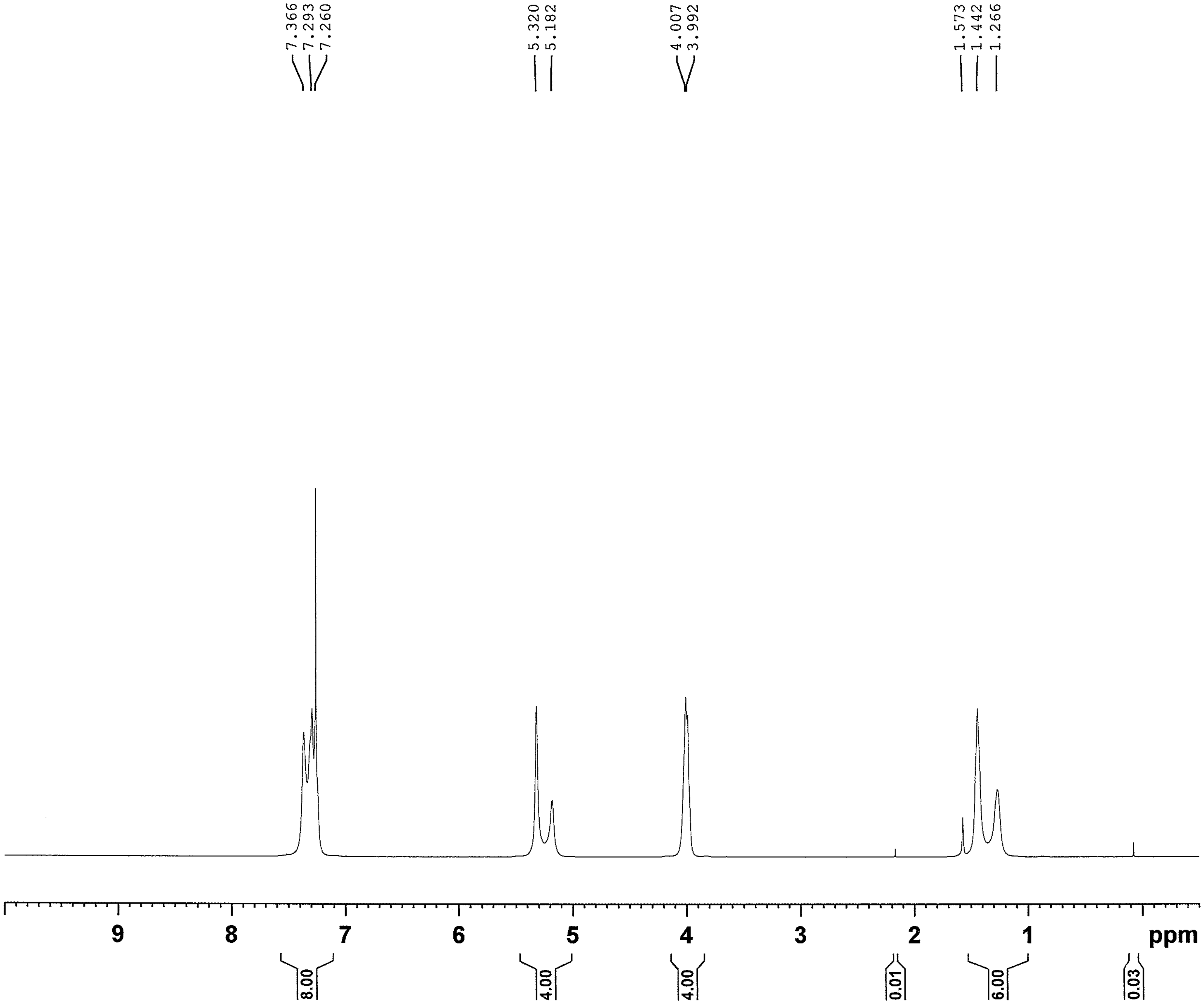


```
Current Data Parameters
NAME              SN0347-L1
EXPNO                    10
PROCNO                    1

F2 - Acquisition Parameters
Date_              20210519
Time                  10.53
INSTRUM               spect
PROBHD     5 mm PABBO BB/
PULPROG                zg30
TD                    32768
SOLVENT               CDCl3
NS                       16
DS                        2
SWH                7978.724 Hz
FIDRES             0.243491 Hz
AQ                2.0534613 sec
RG                    69.13
DW                   62.667 usec
DE                     6.50 usec
TE                    296.2 K
D1               1.00000000 sec
TD0                       1

======== CHANNEL f1 ========
SFO1            399.7524686 MHz
NUC1                     1H
P1                     9.00 usec
PLW1            19.20000076 W

F2 - Processing parameters
SI                    65536
SF              399.7500098 MHz
WDW                      EM
SSB       0
LB                     0.30 Hz
GB        0
PC                     1.00
```

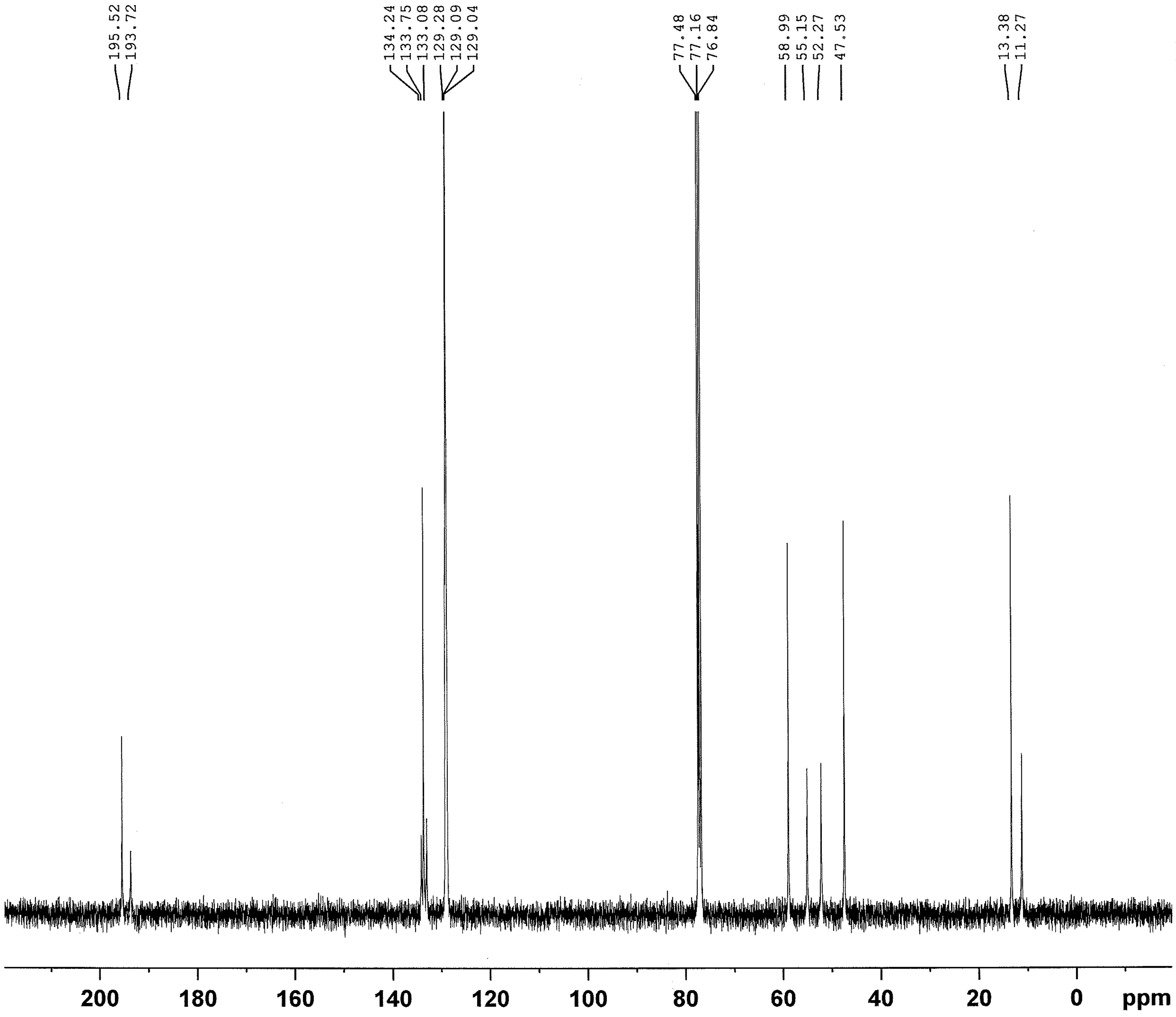

Current Data Parameters
NAME SN0347-LOT1
EXPNO 10
PROCNO 1

F2 - Acquisition Parameters
Date_ 20210521
Time_ 0.24
INSTRUM spect
PROBHD 5 mm PABBO BB/
PULPROG zgpg30
TD 65536
SOLVENT CDCl3
NS 2048
DS 4
SWH 24038.461 Hz
FIDRES 0.366798 Hz
AQ 1.3631488 sec
RG 199.3
DW 20.800 usec
DE 6.50 usec
TE 295.0 K
D1 2.00000000 sec
D11 0.03000000 sec
TD0 1

======== CHANNEL f1 ========
SFO1 100.5272687 MHz
NUC1 13C
P1 8.60 usec
PLW1 87.00000000 W

======== CHANNEL f2 ========
SFO2 399.7515990 MHz
NUC2 1H
CPDPRG[2 waltz16
PCPD2 90.00 usec
PLW2 19.20000076 W
PLW12 0.20000000 W
PLW13 0.15552001 W

F2 - Processing parameters
SI 32768
SF 100.5172106 MHz
WDW EM
SSB 0
LB 1.00 Hz
GB 0
PC 1.40

Title :
Run File : C:\Users\DG\Documents\SYNTHENOVA\Mode opératoire\SN0346-49 421-26\thiuram HPLC\HPLC ok\SN0347-L1.run
Method File : C:\STAR\STARTUP1.MTH
Sample ID : SN0347-L1

Injection Date: 21/05/2021 14:17   Calculation Date: 21/05/2021 14:37

Operator : dG	Detector Type: 0800 (1 Volt)
Workstation: HDD	Bus Address : 81
Instrument : Instrument #1	Sample Rate : 10.00 Hz
Channel : 2 = 2 1	Run Time : 20.002 min

Chart Speed = 1.08 cm/min   Attenuation = 3778   Zero Offset = 2%
Start Time = 0.000 min   End Time = 20.002 min   Min / Tick = 1.00

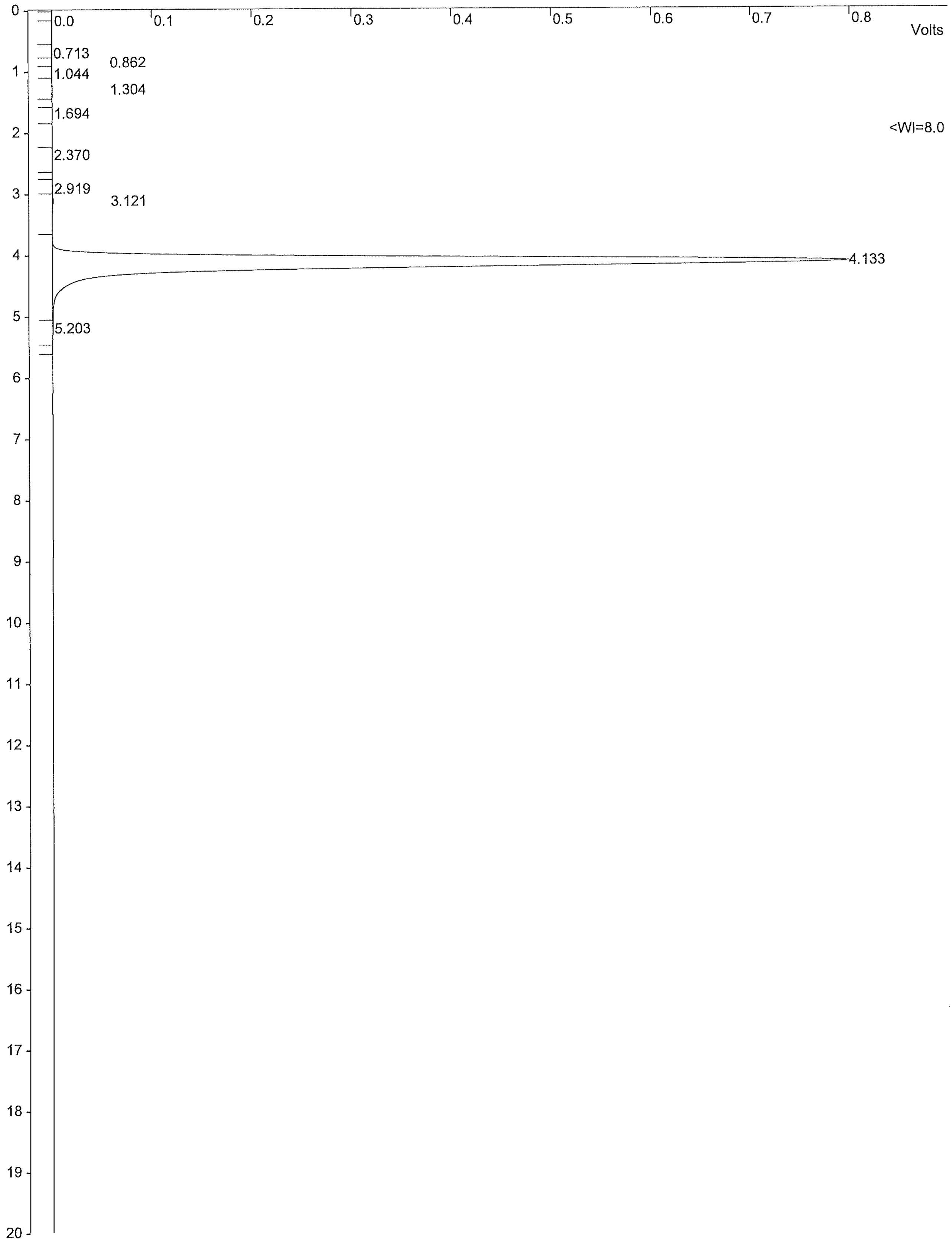

Title :
Run File : C:\Users\DG\Documents\SYNTHENOVA\Mode opératoire\SN0346-49 421-26\thiuram HPLC\HPLC ok\SN0347-L1.run
Method File : C:\STAR\STARTUP1.MTH
Sample ID : SN0347-L1

Injection Date: 21/05/2021 14:17 Calculation Date: 21/05/2021 14:37

Operator : dG
Workstation: HDD
Instrument : Instrument #1
Channel : 2 = 2 1

Detector Type: 0800 (1 Volt)
Bus Address : 81
Sample Rate : 10.00 Hz
Run Time : 20.002 min

Run Mode : Analysis
Peak Measurement: Peak Area
Calculation Type: Percent

Peak No.	Peak Name	Result ()	Ret. Time (min)	Time Offset (min)	Area (counts)	Sep. Code	Width 1/2 (sec)	Status Codes
1		0.0110	0.713	0.000	1018	BV	7.6	
2		0.0123	0.862	0.000	1138	VP	3.2	
3		0.0166	1.044	0.000	1539	PV	5.4	
4		0.0328	1.304	0.000	3048	VV	5.5	
5		0.0236	1.694	0.000	2195	VB	5.0	
6		0.0290	2.370	0.000	2697	BB	6.1	
7		0.0539	2.919	0.000	5010	BV	6.8	
8		0.1185	3.121	0.000	11007	VP	8.2	
9		99.6787	4.133	0.000	9260407	PB	10.2	
10		0.0236	5.203	0.000	2195	TS	0.0	
	Totals:	100.0000		0.000	9290254			

Total Unidentified Counts : 9290255 counts

Detected Peaks: 12 Rejected Peaks: 2 Identified Peaks: 0

Multiplier: 1 Divisor: 1 Unidentified Peak Factor: 1

Baseline Offset: -160 microVolts LSB: 1 microVolts

Noise (used): 37 microVolts - monitored before this run

Manual injection

****

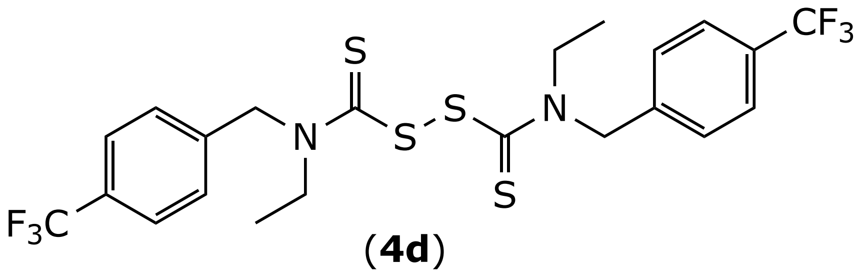

(**4d**)

Chemical Formula: $C_{22}H_{22}F_6N_2S_4$
Exact Mass: 556.0570

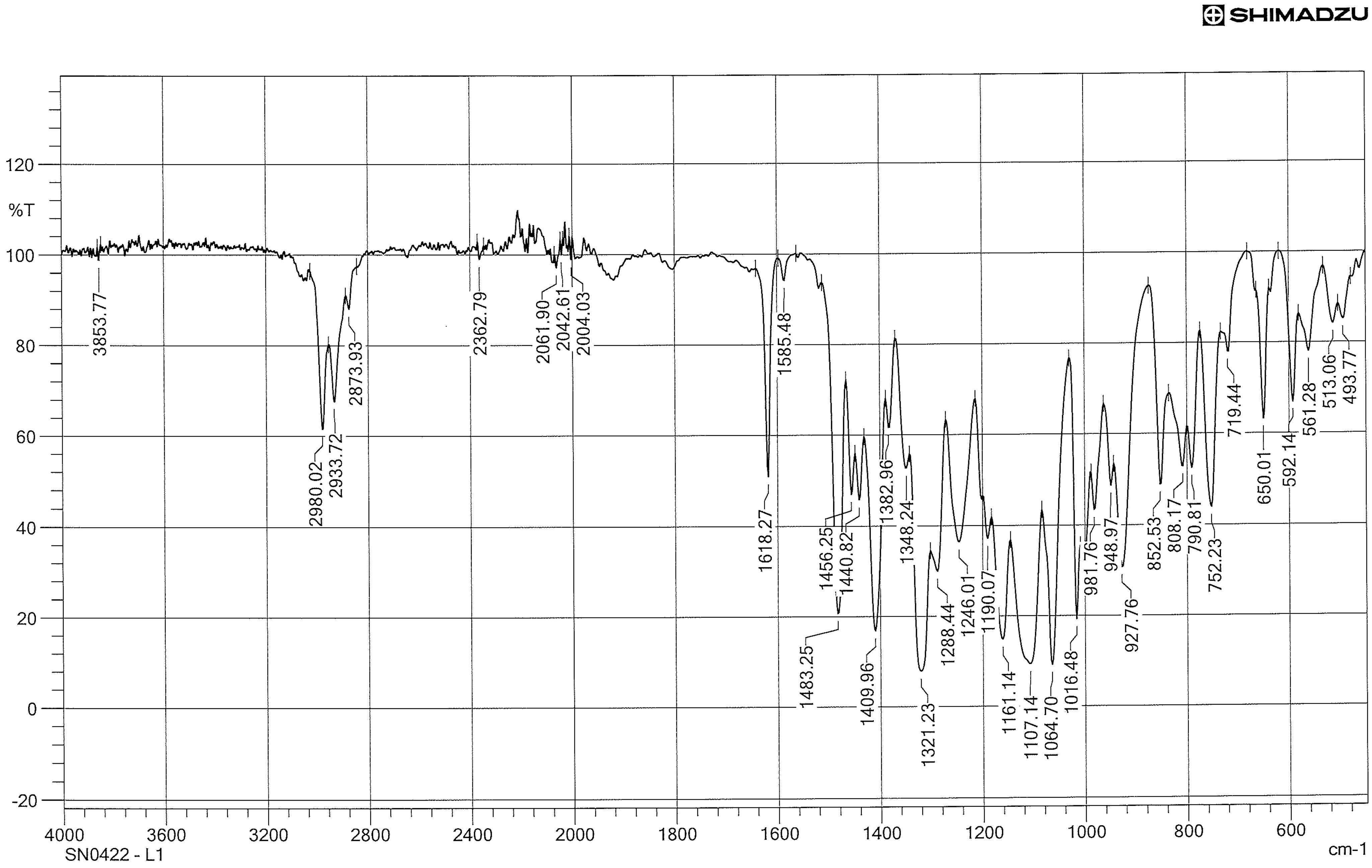

SN0422 – L1

28/05/2021 10:50:57

Elemental Composition Report

Single Mass Analysis

Tolerance = 5.0 PPM / DBE: min = 0.0, max = 14.0
Element prediction: Off
Number of isotope peaks used for i-FIT = 4

Monoisotopic Mass, Even Electron Ions
522 formula(e) evaluated with 2 results within limits (up to 50 best isotopic matches for each mass)
Elements Used:
C: 0-500 H: 0-1000 N: 0-3 S: 0-6 F: 4-7

SN0422 2:14

01-Jun-2021
2106007 612 (3.714) Cm (612:613-(594:595+648:663)) 1: TOF MS ES+ 8.37e+003

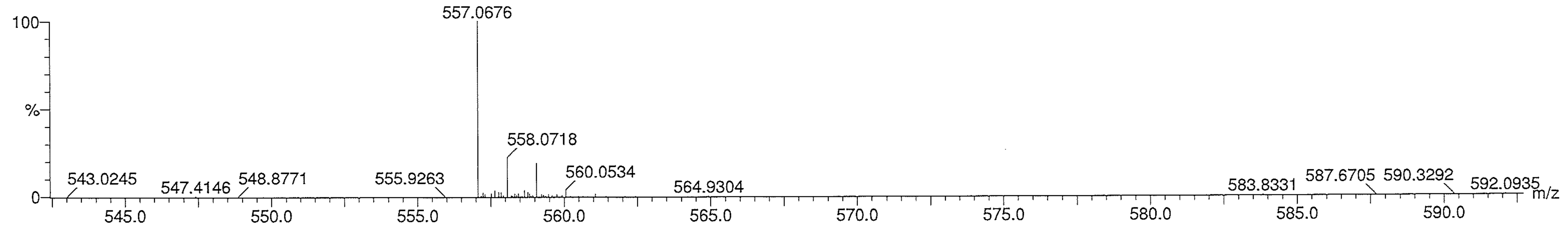

Minimum:				0.0			
Maximum:		5.0	5.0	14.0			
Mass	Calc. Mass	mDa	PPM	DBE	i-FIT	i-FIT (Norm)	Formula
557.0676	557.0648	2.8	5.0	9.5	248.5	0.4	C22 H23 N2 S4 F6
	557.0682	-0.6	-1.1	4.5	249.3	1.2	C19 H27 N2 S5 F6

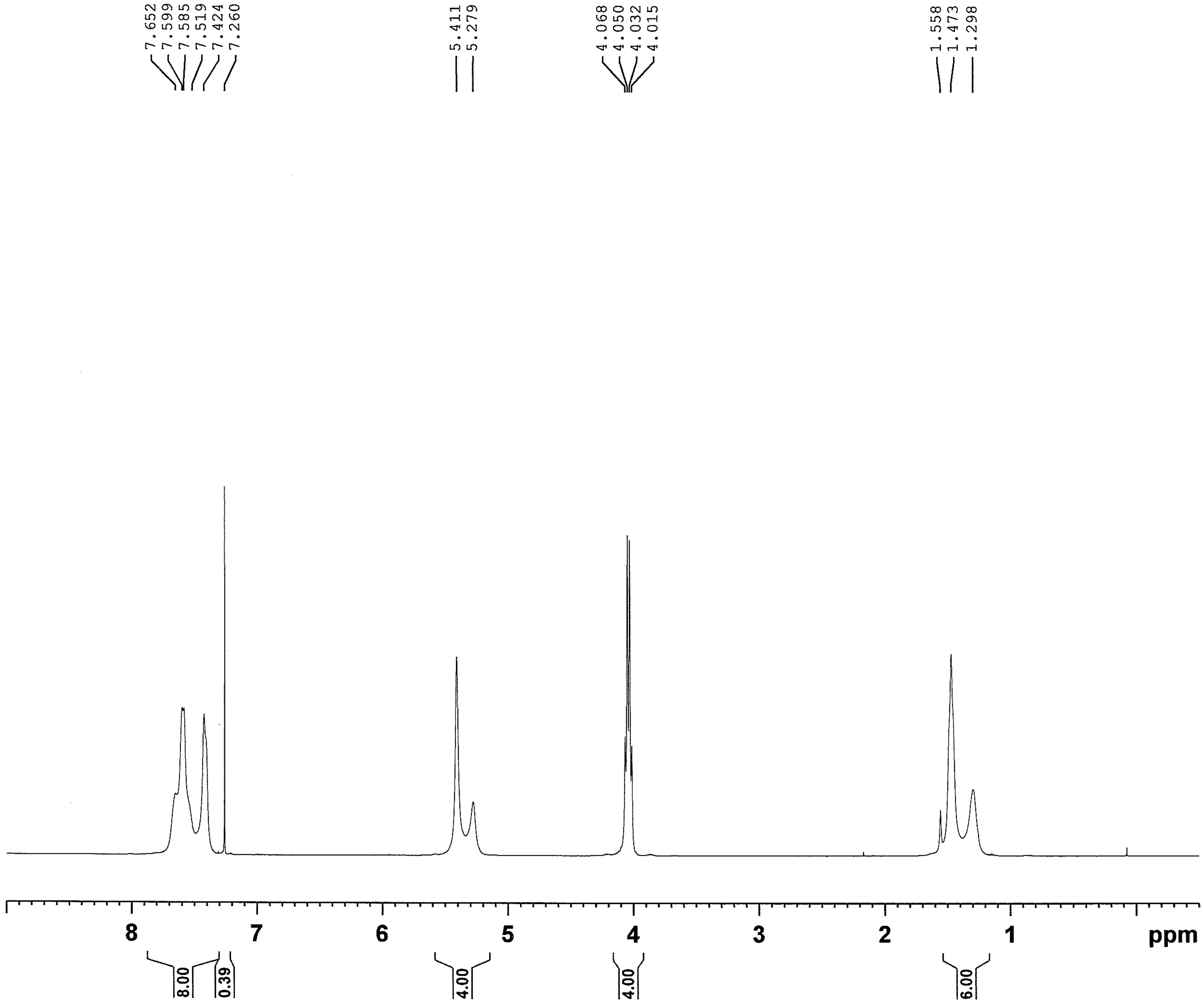


```
Current Data Parameters
NAME              SN0422-L1
EXPNO                    10
PROCNO                    1

F2 - Acquisition Parameters
Date_              20210526
Time                  18.01
INSTRUM               spect
PROBHD     5 mm PABBO BB/
PULPROG                zg30
TD                    32768
SOLVENT               CDCl3
NS                       16
DS                        2
SWH                7978.724 Hz
FIDRES             0.243491 Hz
AQ                2.0534613 sec
RG                     79.2
DW                   62.667 usec
DE                     6.50 usec
TE                    296.2 K
D1               1.00000000 sec
TD0                       1

======== CHANNEL f1 ========
SFO1            399.7524686 MHz
NUC1                     1H
P1                     9.00 usec
PLW1            19.20000076 W

F2 - Processing parameters
SI                    65536
SF              399.7500098 MHz
WDW                      EM
SSB      0
LB                     0.30 Hz
GB       0
PC                     1.00
```

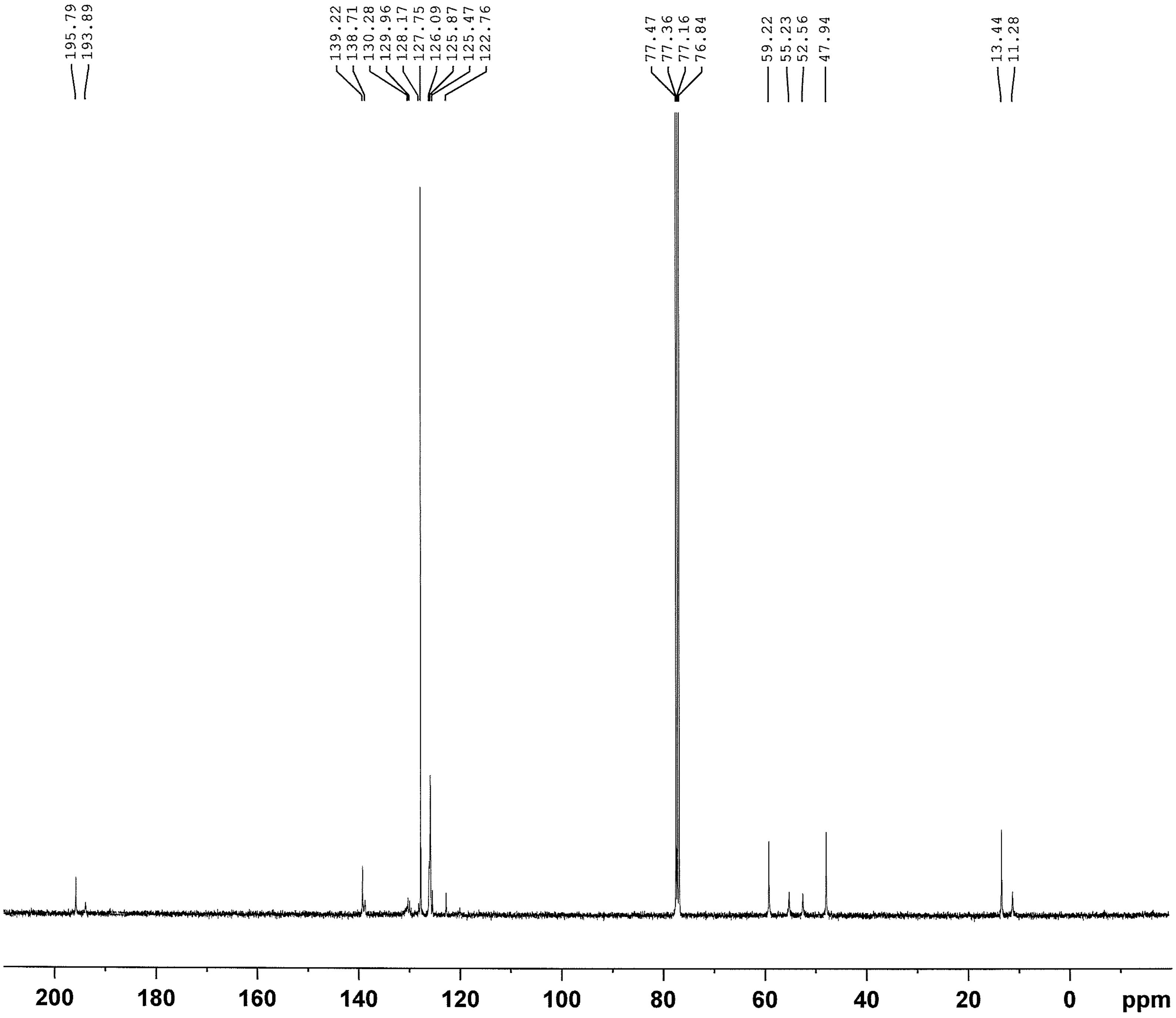

Current Data Parameters

NAME	SN0422-lot1
EXPNO	10
PROCNO	1

F2 - Acquisition Parameters

Date_	20210527
Time	23.18
INSTRUM	spect
PROBHD	5 mm PABBO BB/
PULPROG	zgpg30
TD	65536
SOLVENT	CDCl3
NS	2048
DS	4
SWH	24038.461 Hz
FIDRES	0.366798 Hz
AQ	1.3631488 sec
RG	199.3
DW	20.800 usec
DE	6.50 usec
TE	295.0 K
D1	2.00000000 sec
D11	0.03000000 sec
TD0	1

======== CHANNEL f1 ========

SFO1	100.5272687 MHz
NUC1	13C
P1	8.60 usec
PLW1	87.00000000 W

======== CHANNEL f2 ========

SFO2	399.7515990 MHz
NUC2	1H
CPDPRG[2	waltz16
PCPD2	90.00 usec
PLW2	19.20000076 W
PLW12	0.20000000 W
PLW13	0.15552001 W

F2 - Processing parameters

SI	32768
SF	100.5172078 MHz
WDW	EM
SSB	0
LB	1.00 Hz
GB	0
PC	1.40

```
Title        :
Run File     : C:\Users\DG\Documents\SYNTHENOVA\Mode opératoire\SN0346-49 421-26\thiuram HPLC\HPLC ok\SN0422-L1.run
Method File  : C:\star\startup1.mth
Sample ID    : SN0422-L1

Injection Date: 26/05/2021 15:42    Calculation Date: 26/05/2021 16:02

Operator    : dG                    Detector Type: 0800 (1 Volt)
Workstation: HDD                    Bus Address  : 81
Instrument : Instrument #1          Sample Rate  : 10.00 Hz
Channel     : 2 = 2 1               Run Time     : 20.002 min


Chart Speed =    1.08 cm/min   Attenuation = 1959          Zero Offset = 2%
Start Time  =   0.000   min     End Time    = 20.002  min   Min / Tick  = 1.00
```

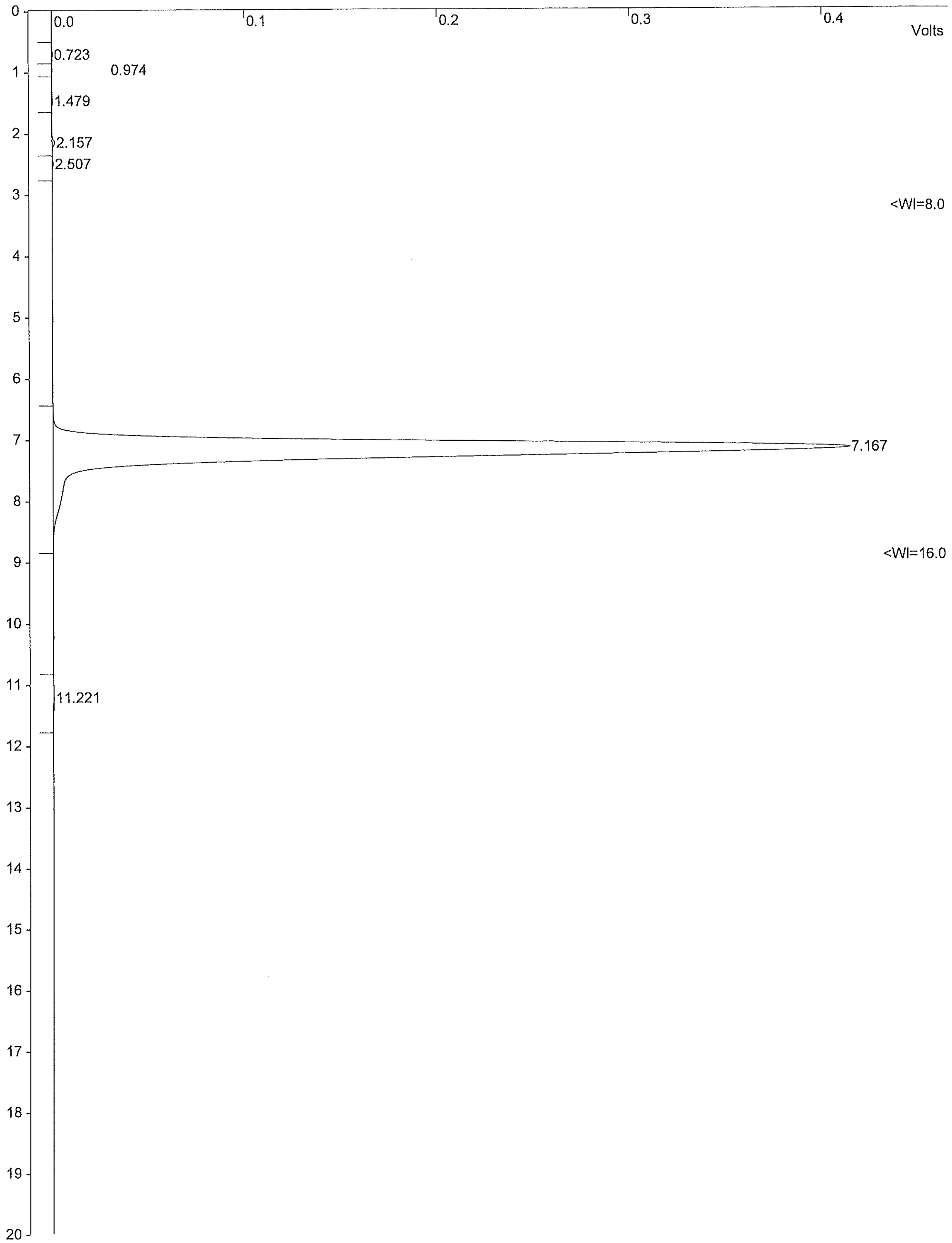

Title :
Run File : C:\Users\DG\Documents\SYNTHENOVA\Mode opératoire\SN0346-49 421-26\thiuram HPLC\HPLC ok\SN0422-L1.run
Method File : C:\star\startup1.mth
Sample ID : SN0422-L1

Injection Date: 26/05/2021 15:42 Calculation Date: 26/05/2021 16:02

Operator : dG
Workstation: HDD
Instrument : Instrument #1
Channel : 2 = 2 1

Detector Type: 0800 (1 Volt)
Bus Address : 81
Sample Rate : 10.00 Hz
Run Time : 20.002 min

Run Mode : Analysis
Peak Measurement: Peak Area
Calculation Type: Percent

Peak No.	Peak Name	Result ()	Ret. Time (min)	Time Offset (min)	Area (counts)	Sep. Code	Width 1/2 (sec)	Status Codes
1		0.0483	0.723	0.000	3523	BP	8.8	
2		0.0212	0.974	0.000	1544	PP	6.6	
3		0.0646	1.479	0.000	4706	PV	7.3	
4		0.1678	2.157	0.000	12235	VV	6.4	
5		0.0671	2.507	0.000	4893	VB	7.3	
6		99.5413	7.167	0.000	7257482	BB	15.6	
7		0.0897	11.221	0.000	6539	BB	22.8	
	Totals:	100.0000		0.000	7290922			

Total Unidentified Counts : 7290923 counts

Detected Peaks: 7 Rejected Peaks: 0 Identified Peaks: 0

Multiplier: 1 Divisor: 1 Unidentified Peak Factor: 1

Baseline Offset: -12 microVolts LSB: 1 microVolts

Noise (used): 68 microVolts - monitored before this run

Manual injection

****

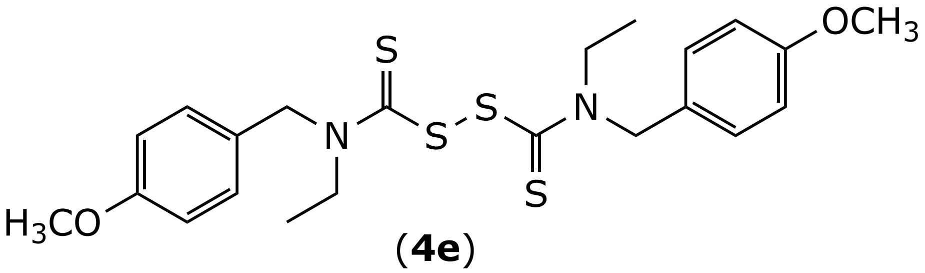

(**4e**)

Chemical Formula: $C_{22}H_{28}N_2O_2S_4$
Exact Mass: 480.1034

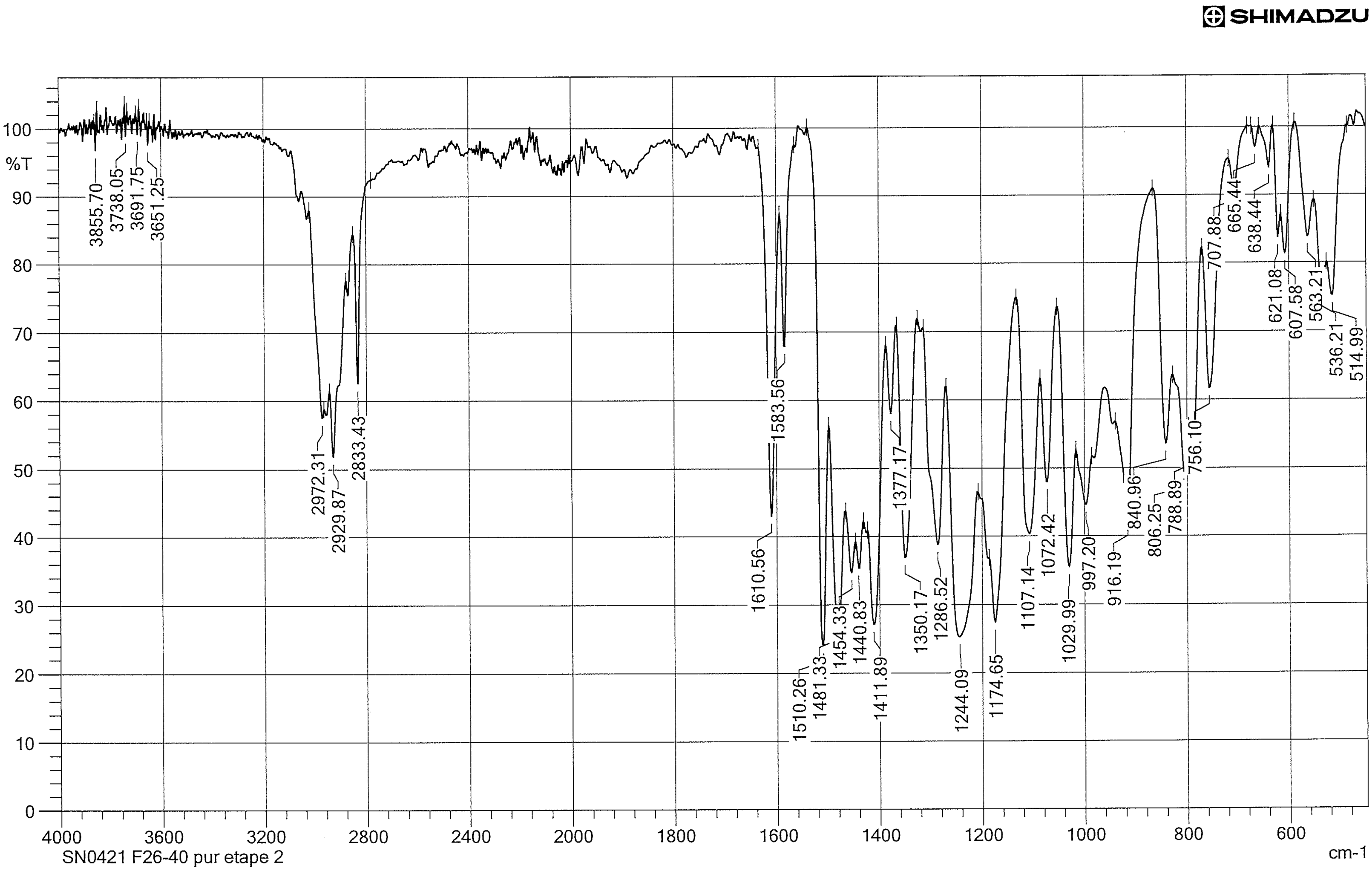

SN0421 F26-40 pur etape 2

SN0421

28/04/2021 08:37:49

Elemental Composition Report

Single Mass Analysis

Tolerance = 5.0 PPM / DBE: min = 0.0, max = 14.0
Element prediction: Off
Number of isotope peaks used for i-FIT = 4

Monoisotopic Mass, Even Electron Ions
560 formula(e) evaluated with 1 results within limits (up to 50 best isotopic matches for each mass)
Elements Used:
C: 0-500 H: 0-1000 N: 0-3 O: 0-3 S: 0-6

SN0421 2:13

01-Jun-2021
2106006 554 (3.365) Cm (552:557) 1: TOF MS ES+ 5.62e+004

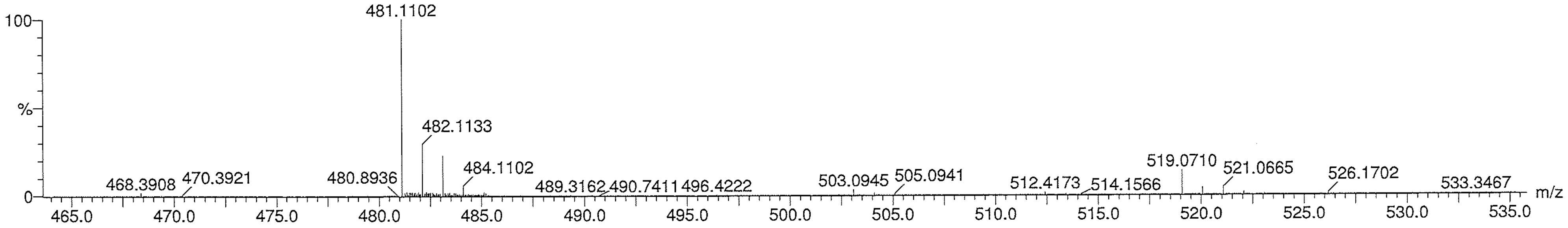

	Mass	Calc. Mass	mDa	PPM	DBE	i-FIT	i-FIT (Norm)	Formula
Minimum:					0.0			
Maximum:			5.0	5.0	14.0			
	481.1102	481.1112	-1.0	-2.1	9.5	690.6	0.0	C22 H29 N2 O2 S4

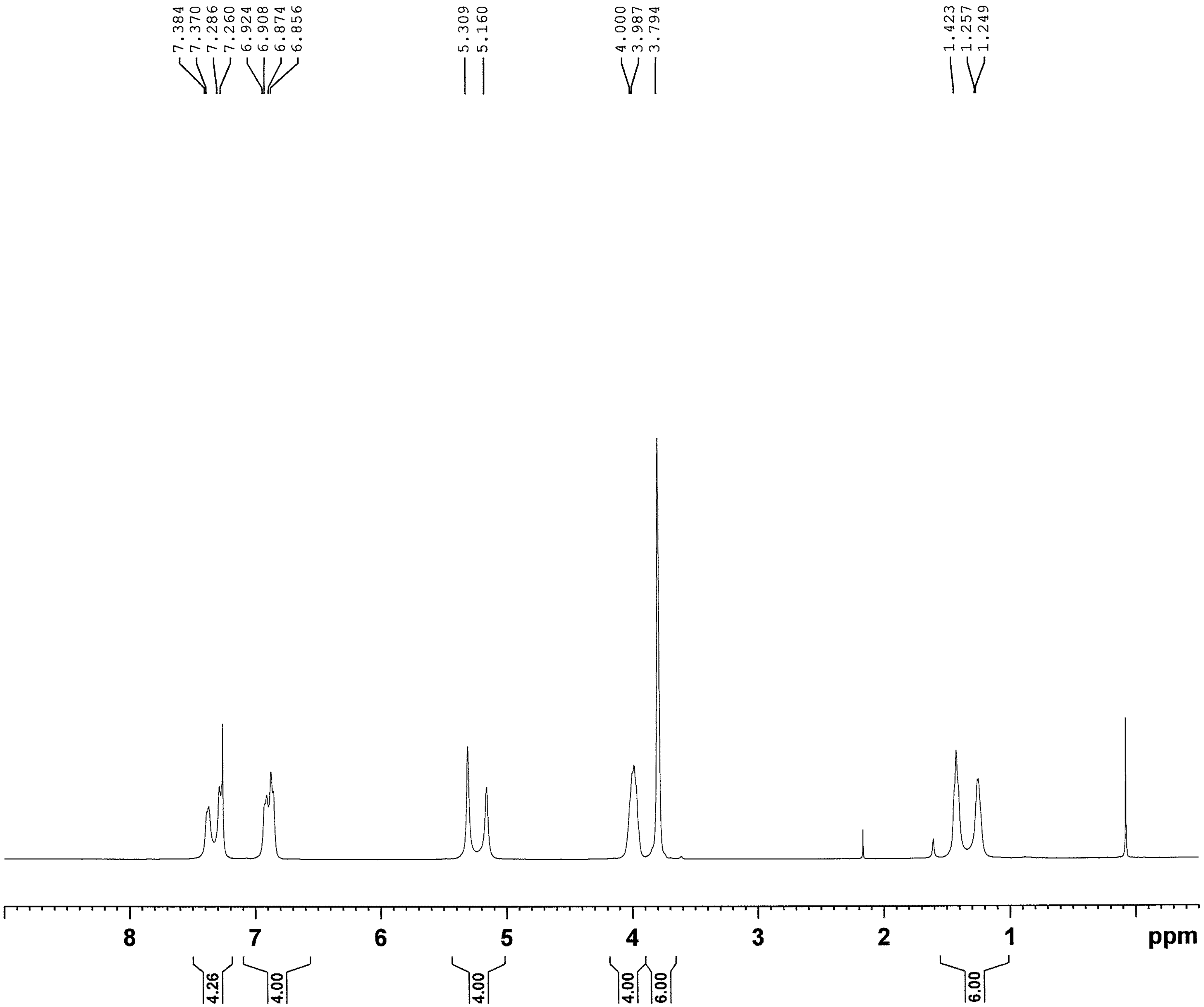

Current Data Parameters
NAME SN0421
EXPNO 10
PROCNO 1

F2 - Acquisition Parameters
Date_ 20210427
Time_ 17.55
INSTRUM spect
PROBHD 5 mm PABBO BB/
PULPROG zg30
TD 32768
SOLVENT CDCl3
NS 16
DS 2
SWH 7978.724 Hz
FIDRES 0.243491 Hz
AQ 2.0534613 sec
RG 30.7
DW 62.667 usec
DE 6.50 usec
TE 296.2 K
D1 2.00000000 sec
TD0 1

======== CHANNEL f1 ========
SFO1 399.7524686 MHz
NUC1 1H
P1 9.00 usec
PLW1 19.20000076 W

F2 - Processing parameters
SI 65536
SF 399.7500098 MHz
WDW EM
SSB 0
LB 0.30 Hz
GB 0
PC 1.00

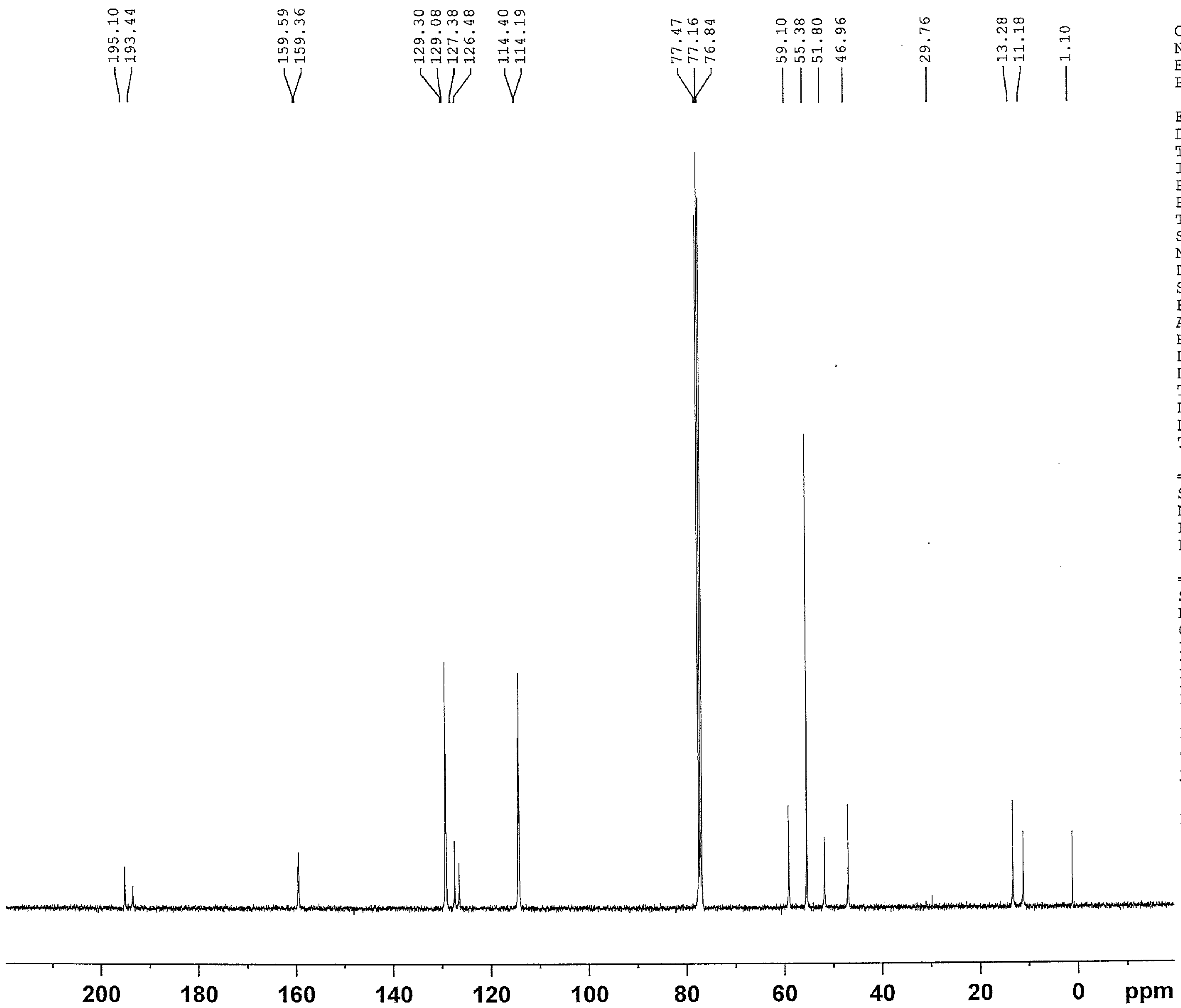

Current Data Parameters
NAME SN0421-L1
EXPNO 10
PROCNO 1

F2 - Acquisition Parameters
Date_ 20210501
Time 20.34
INSTRUM spect
PROBHD 5 mm PABBO BB/
PULPROG zgpg30
TD 65536
SOLVENT CDCl3
NS 2048
DS 4
SWH 24038.461 Hz
FIDRES 0.366798 Hz
AQ 1.3631488 sec
RG 199.3
DW 20.800 usec
DE 6.50 usec
TE 295.0 K
D1 2.00000000 sec
D11 0.03000000 sec
TD0 1

======== CHANNEL f1 ========
SFO1 100.5272687 MHz
NUC1 13C
P1 8.60 usec
PLW1 87.00000000 W

======== CHANNEL f2 ========
SFO2 399.7515990 MHz
NUC2 1H
CPDPRG[2 waltz16
PCPD2 90.00 usec
PLW2 19.20000076 W
PLW12 0.20000000 W
PLW13 0.15552001 W

F2 - Processing parameters
SI 32768
SF 100.5172136 MHz
WDW EM
SSB 0
LB 1.00 Hz
GB 0
PC 1.40

Title :
Run File : C:\Users\DG\Documents\SYNTHENOVA\Mode opératoire\SN0346-49 421-26\thiuram HPLC\HPLC ok\SN0421-L1.run
Method File : C:\star\startup1.mth
Sample ID : SN0421-L1

Injection Date: 12/05/2021 17:16   Calculation Date: 12/05/2021 17:36

Operator : dG	Detector Type: 0800 (1 Volt)
Workstation: HDD	Bus Address : 81
Instrument : Instrument #1	Sample Rate : 10.00 Hz
Channel : 2 = 2 1	Run Time : 20.002 min

Chart Speed = 1.08 cm/min	Attenuation = 3035	Zero Offset = 2%
Start Time = 0.000 min	End Time = 20.002 min	Min / Tick = 1.00

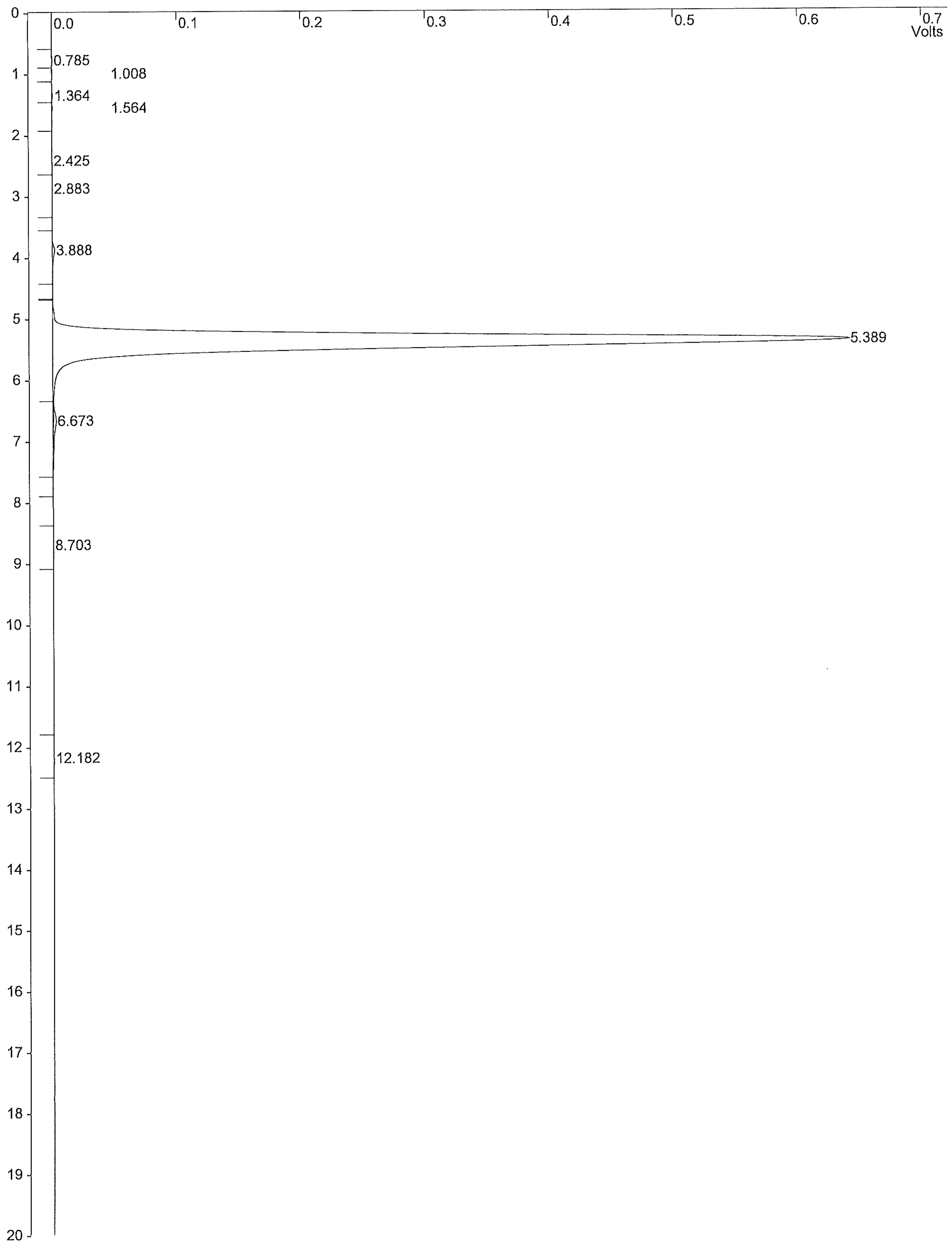

Title :
Run File : C:\Users\DG\Documents\SYNTHENOVA\Mode opératoire\SN0346-49 421-26\thiuram HPLC\HPLC ok\SN0421-L1.run
Method File : C:\star\startup1.mth
Sample ID : SN0421-L1

Injection Date: 12/05/2021 17:16 Calculation Date: 12/05/2021 17:36

Operator : dG
Workstation: HDD
Instrument : Instrument #1
Channel : 2 = 2 1

Detector Type: 0800 (1 Volt)
Bus Address : 81
Sample Rate : 10.00 Hz
Run Time : 20.002 min

Run Mode : Analysis
Peak Measurement: Peak Area
Calculation Type: Percent

Peak No.	Peak Name	Result ()	Ret. Time (min)	Time Offset (min)	Area (counts)	Sep. Code	Width 1/2 (sec)	Status Codes
1		0.0469	0.785	0.000	4502	BV	7.8	
2		0.0185	1.008	0.000	1772	VV	10.3	
3		0.0628	1.364	0.000	6027	VV	7.0	
4		0.0660	1.564	0.000	6334	VV	7.5	
5		0.0466	2.425	0.000	4470	VV	0.0	
6		0.0438	2.883	0.000	4201	VB	10.1	
7		0.3073	3.888	0.000	29472	BB	11.2	
8		98.7962	5.389	0.000	9475442	VB	13.2	
9		0.5346	6.673	0.000	51273	TS	0.0	
10		0.0537	8.703	0.000	5148	BB	15.3	
11		0.0235	12.182	0.000	2256	BB	0.0	
	Totals:	99.9999		0.000	9590897			

Total Unidentified Counts : 9590898 counts

Detected Peaks: 12 Rejected Peaks: 1 Identified Peaks: 0

Multiplier: 1 Divisor: 1 Unidentified Peak Factor: 1

Baseline Offset: -38 microVolts LSB: 1 microVolts

Noise (used): 34 microVolts - monitored before this run

Manual injection

****

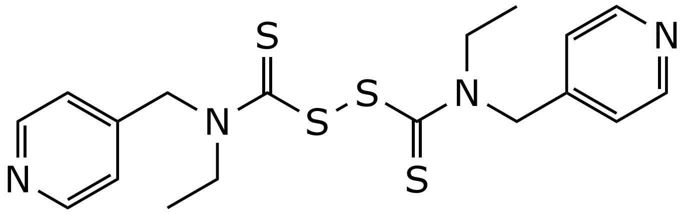

(**4f**)

Chemical Formula: $C_{18}H_{22}N_4S_4$
Exact Mass: 422.0727

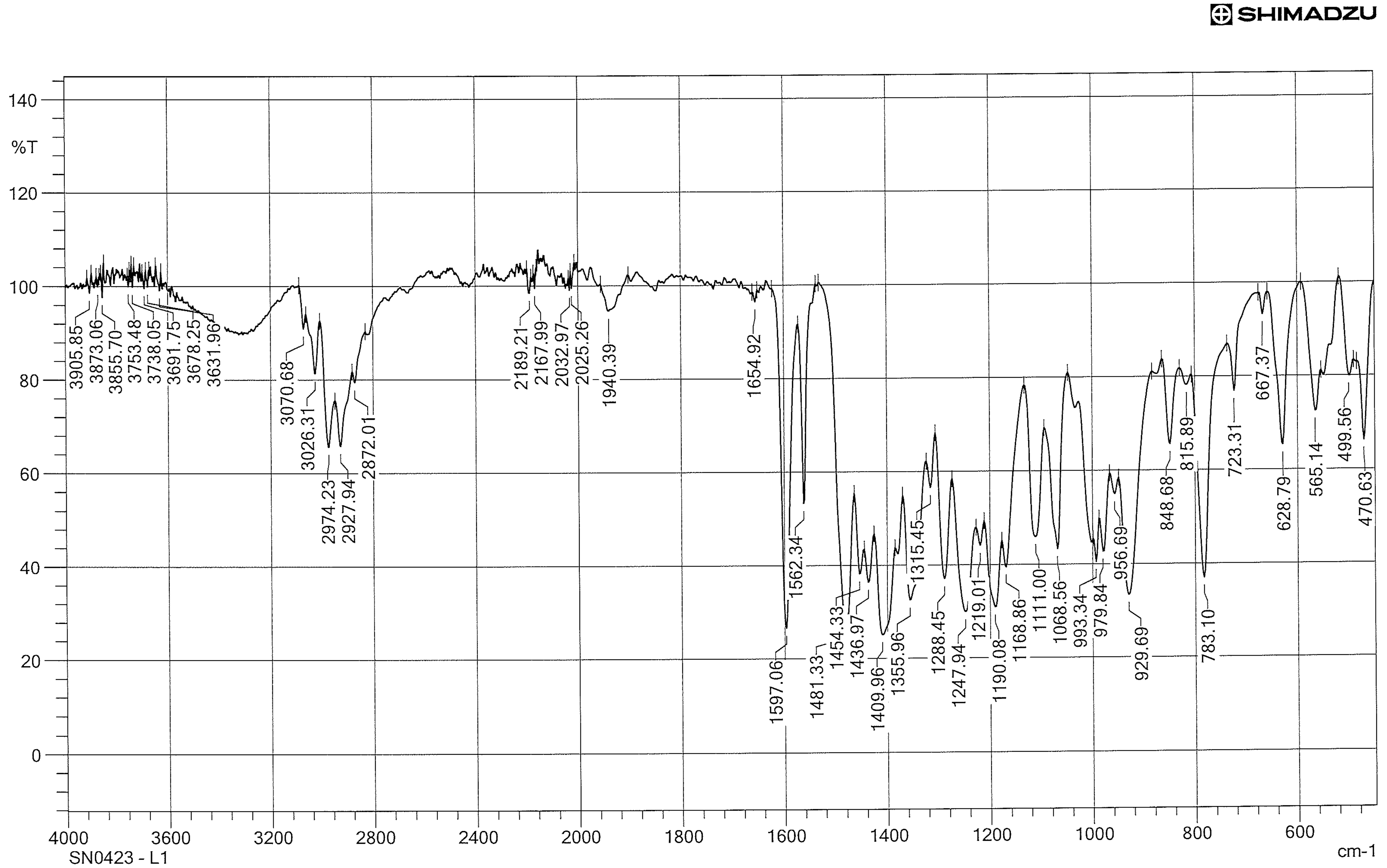

SN0423 – L1

20/05/2021 11:35:02

Elemental Composition Report

Single Mass Analysis
Tolerance = 5.0 PPM / DBE: min = 0.0, max = 14.0
Element prediction: Off
Number of isotope peaks used for i-FIT = 4

Monoisotopic Mass, Even Electron Ions
189 formula(e) evaluated with 1 results within limits (up to 50 best isotopic matches for each mass)
Elements Used:
C: 0-500 H: 0-1000 N: 0-5 S: 0-6

SN0423 2:15

01-Jun-2021
2106008 360 (2.176) Cm (360-(343+391)) 1: TOF MS ES+ 6.39e+003

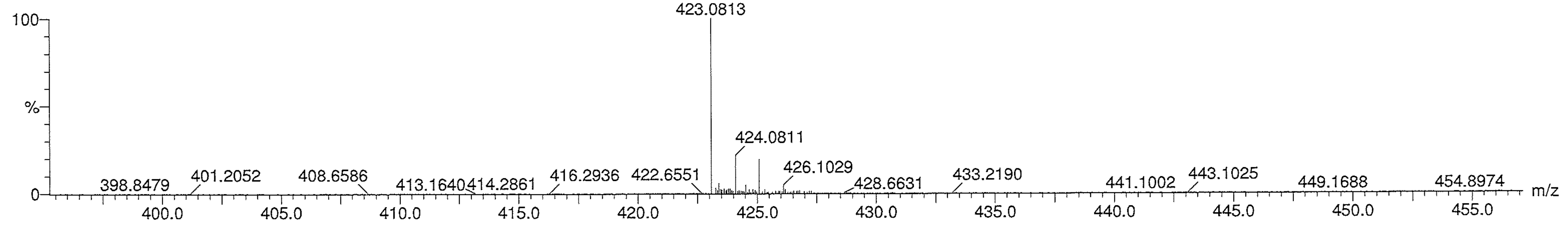

Minimum:				0.0			
Maximum:		5.0	5.0	14.0			
Mass	Calc. Mass	mDa	PPM	DBE	i-FIT	i-FIT (Norm)	Formula
423.0813	423.0806	0.7	1.7	9.5	357.3	0.0	C18 H23 N4 S4

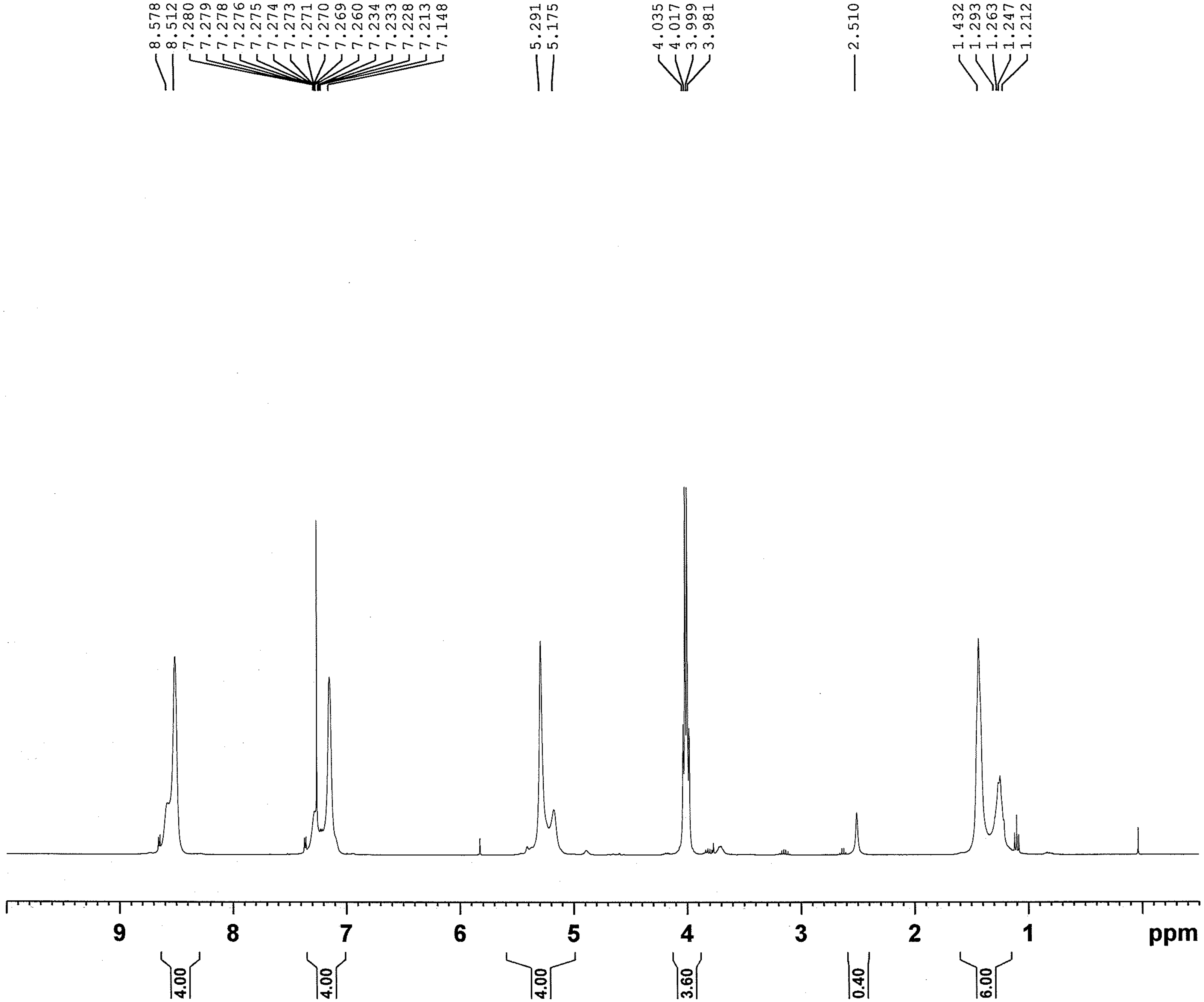

Current Data Parameters
NAME SN0423-L1
EXPNO 10
PROCNO 1

F2 - Acquisition Parameters
Date_ 20210520
Time 17.47
INSTRUM spect
PROBHD 5 mm PABBO BB/
PULPROG zg30
TD 32768
SOLVENT CDCl3
NS 16
DS 2
SWH 7978.724 Hz
FIDRES 0.243491 Hz
AQ 2.0534613 sec
RG 30.7
DW 62.667 usec
DE 6.50 usec
TE 296.2 K
D1 1.00000000 sec
TD0 1

======== CHANNEL f1 ========
SFO1 399.7524686 MHz
NUC1 1H
P1 9.00 usec
PLW1 19.20000076 W

F2 - Processing parameters
SI 65536
SF 399.7500100 MHz
WDW EM
SSB 0
LB 0.30 Hz
GB 0
PC 1.00

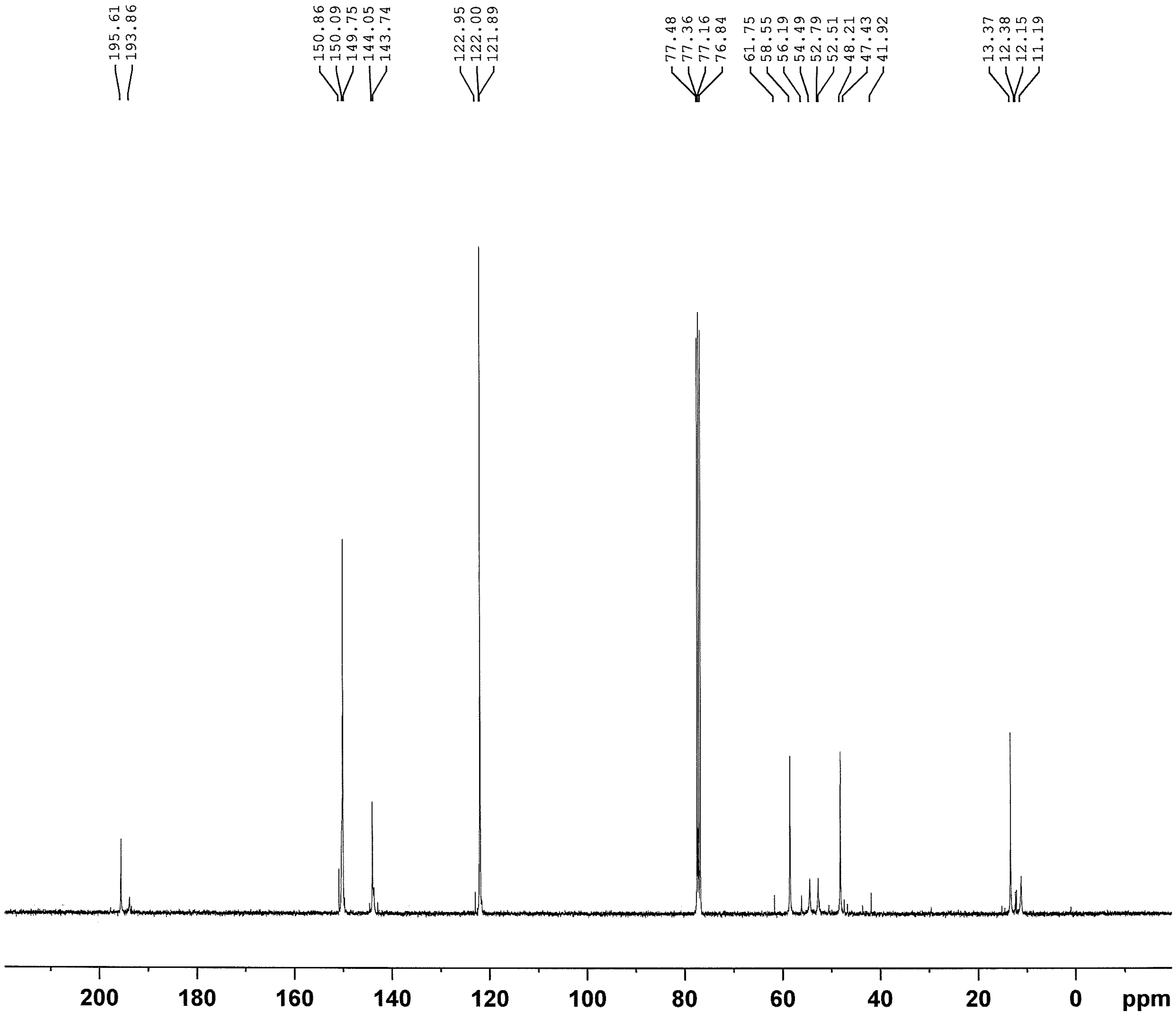

Current Data Parameters
NAME SN0423-LOT1
EXPNO 10
PROCNO 1

F2 - Acquisition Parameters
Date_ 20210523
Time 9.02
INSTRUM spect
PROBHD 5 mm PABBO BB/
PULPROG zgpg30
TD 65536
SOLVENT CDCl3
NS 2048
DS 4
SWH 24038.461 Hz
FIDRES 0.366798 Hz
AQ 1.3631488 sec
RG 199.3
DW 20.800 usec
DE 6.50 usec
TE 295.0 K
D1 2.00000000 sec
D11 0.03000000 sec
TD0 1

======== CHANNEL f1 ========
SFO1 100.5272687 MHz
NUC1 13C
P1 8.60 usec
PLW1 87.00000000 W

======== CHANNEL f2 ========
SFO2 399.7515990 MHz
NUC2 1H
CPDPRG[2 waltz16
PCPD2 90.00 usec
PLW2 19.20000076 W
PLW12 0.20000000 W
PLW13 0.15552001 W

F2 - Processing parameters
SI 32768
SF 100.5172217 MHz
WDW EM
SSB 0
LB 1.00 Hz
GB 0
PC 1.40

Title :
Run File : C:\Users\DG\Documents\SYNTHENOVA\Mode opératoire\SN0346-49 421-26\thiuram HPLC\HPLC ok\SN0423-L1.run
Method File : C:\star\startup1.mth
Sample ID : SN0423-L1

Injection Date: 27/05/2021 10:55   Calculation Date: 27/05/2021 11:15

Operator : DG   Detector Type: 0800 (1 Volt)
Workstation: HDD   Bus Address : 81
Instrument : Instrument #1   Sample Rate : 10.00 Hz
Channel : 2 = 2 1   Run Time : 20.002 min

Chart Speed = 1.08 cm/min   Attenuation = 3112   Zero Offset = 2%
Start Time = 0.000 min   End Time = 20.002 min   Min / Tick = 1.00

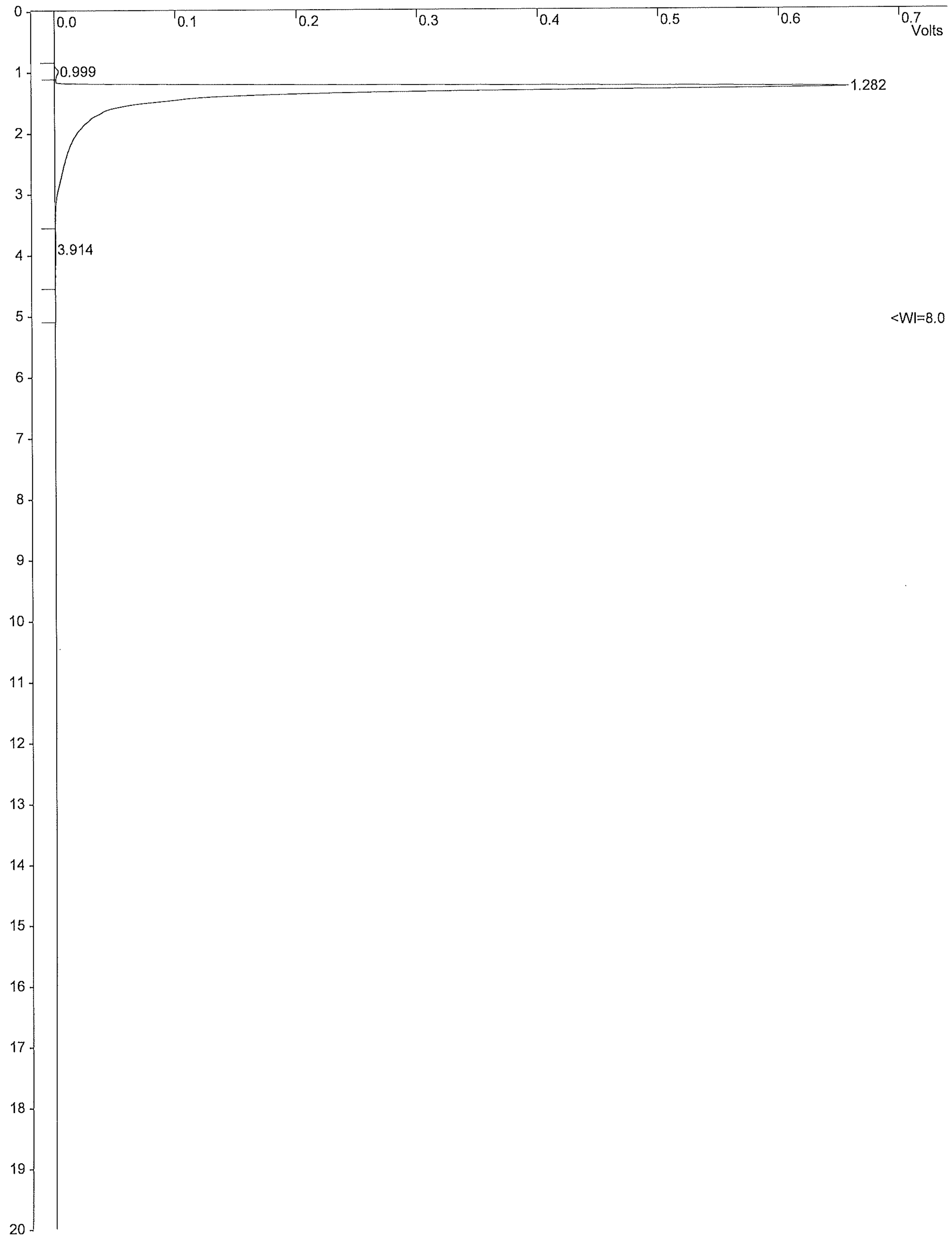

Title :
Run File : C:\Users\DG\Documents\SYNTHENOVA\Mode opératoire\SN0346-49 421-26\thiuram HPLC\HPLC ok\SN0423-L1.run
Method File : C:\star\startup1.mth
Sample ID : SN0423-L1

Injection Date: 27/05/2021 10:55 Calculation Date: 27/05/2021 11:15

Operator : DG
Workstation: HDD
Instrument : Instrument #1
Channel : 2 = 2 1

Detector Type: 0800 (1 Volt)
Bus Address : 81
Sample Rate : 10.00 Hz
Run Time : 20.002 min

Run Mode : Analysis
Peak Measurement: Peak Area
Calculation Type: Percent

Peak No.	Peak Name	Result ()	Ret. Time (min)	Time Offset (min)	Area (counts)	Sep. Code	Width 1/2 (sec)	Status Codes
1		0.3955	0.999	0.000	26791	BV	7.7	
2		99.4729	1.282	0.000	6738153	VB	6.2	
3		0.1316	3.914	0.000	8912	TS	0.0	
	Totals:	100.0000		0.000	6773856			

Total Unidentified Counts : 6773856 counts

Detected Peaks: 3 Rejected Peaks: 0 Identified Peaks: 0

Multiplier: 1 Divisor: 1 Unidentified Peak Factor: 1

Baseline Offset: 4 microVolts LSB: 1 microVolts

Noise (used): 35 microVolts - monitored before this run

Manual injection

****

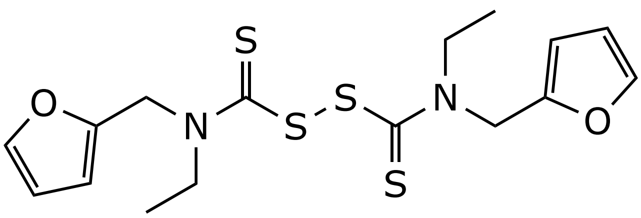

(**4g**)

Chemical Formula: $C_{16}H_{20}N_2O_2S_4$
Exact Mass: 400.0408

SHIMADZU

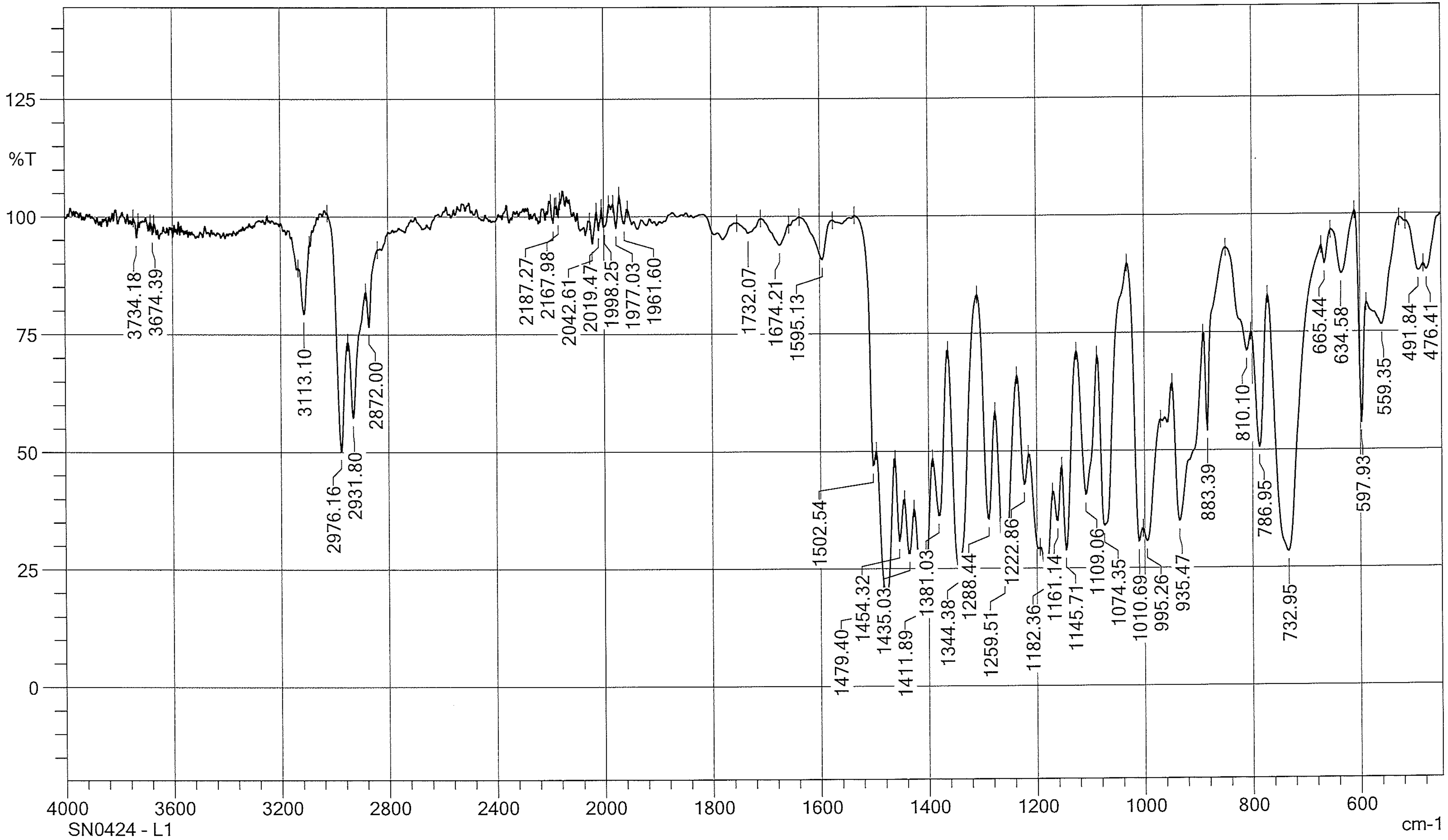

SN0424 – L1

28/05/2021 14:28:28

Elemental Composition Report

Single Mass Analysis

Tolerance = 5.0 PPM / DBE: min = 0.0, max = 14.0
Element prediction: Off
Number of isotope peaks used for i-FIT = 4

Monoisotopic Mass, Even Electron Ions
662 formula(e) evaluated with 1 results within limits (up to 50 best isotopic matches for each mass)
Elements Used:
C: 0-500 H: 0-1000 N: 0-5 O: 0-3 S: 0-6

SN0424 2:16

01-Jun-2021
2106009 510 (3.078) Cm (508:513) 1: TOF MS ES+ 7.96e+004

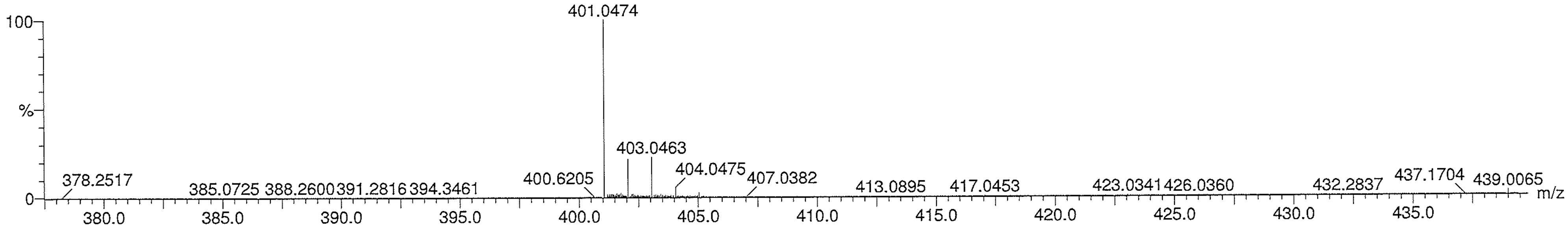

Minimum:				0.0			
Maximum:		5.0	5.0	14.0			
Mass	Calc. Mass	mDa	PPM	DBE	i-FIT	i-FIT (Norm)	Formula
401.0474	401.0486	-1.2	-3.0	7.5	866.2	0.0	C16 H21 N2 O2 S4

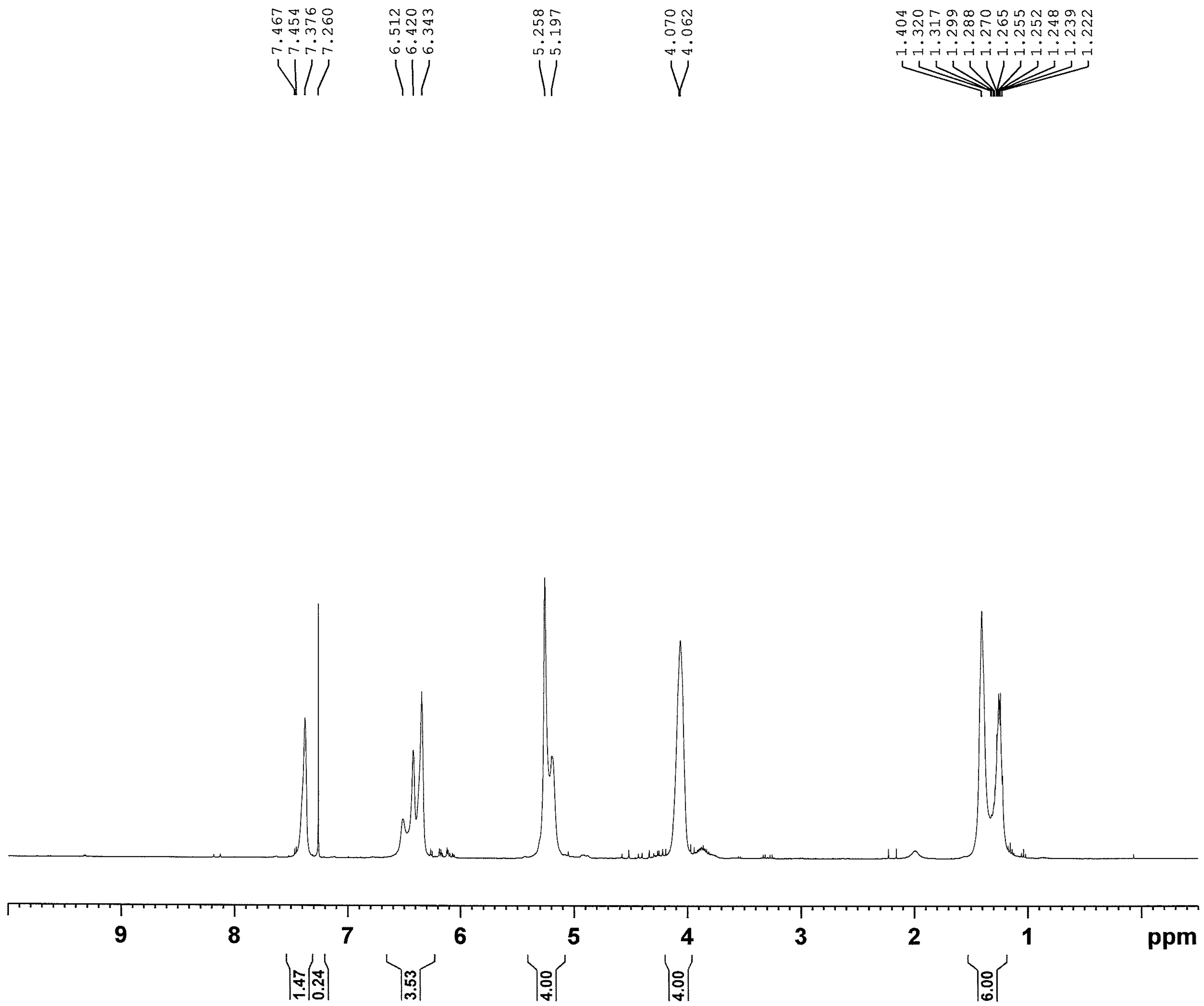


```
Current Data Parameters
NAME           SN0424LOT1
EXPNO                  10
PROCNO                  1

F2 - Acquisition Parameters
Date_            20210523
Time                 5.00
INSTRUM             spect
PROBHD    5 mm PABBO BB/
PULPROG              zg30
TD                  32768
SOLVENT             CDCl3
NS                     16
DS                      2
SWH              7978.724 Hz
FIDRES           0.243491 Hz
AQ              2.0534613 sec
RG                   30.7
DW                 62.667 usec
DE                   6.50 usec
TE                  296.2 K
D1             1.00000000 sec
TD0                     1

======== CHANNEL f1 ========
SFO1        399.7524686 MHz
NUC1                   1H
P1                   9.00 usec
PLW1         19.20000076 W

F2 - Processing parameters
SI                  65536
SF          399.7500098 MHz
WDW                    EM
SSB       0
LB                   0.30 Hz
GB        0
PC                   1.00
```

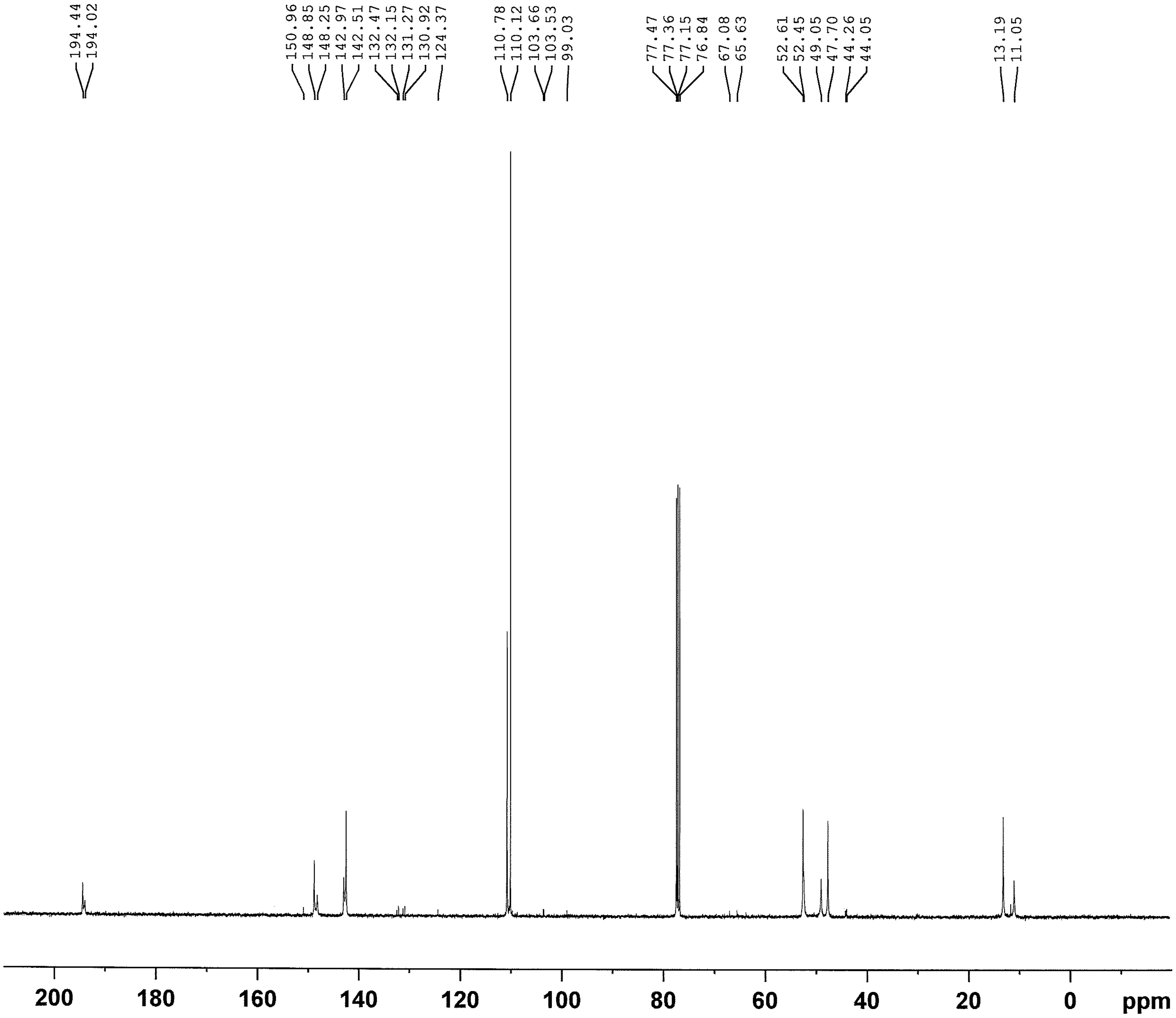


```
Current Data Parameters
NAME           SN0424LOT1
EXPNO                  11
PROCNO                  1

F2 - Acquisition Parameters
Date_            20210523
Time_                7.00
INSTRUM             spect
PROBHD    5 mm PABBO BB/
PULPROG           zgpg30
TD                 65536
SOLVENT            CDCl3
NS                  2048
DS                     4
SWH            24038.461 Hz
FIDRES          0.366798 Hz
AQ             1.3631488 sec
RG                 199.3
DW                20.800 usec
DE                  6.50 usec
TE                 295.0 K
D1            2.00000000 sec
D11           0.03000000 sec
TD0                    1

======== CHANNEL f1 ========
SFO1       100.5272687 MHz
NUC1               13C
P1                8.60 usec
PLW1       87.00000000 W

======== CHANNEL f2 ========
SFO2       399.7515990 MHz
NUC2                1H
CPDPRG[2        waltz16
PCPD2            90.00 usec
PLW2       19.20000076 W
PLW12       0.20000000 W
PLW13       0.15552001 W

F2 - Processing parameters
SI                32768
SF         100.5172175 MHz
WDW                  EM
SSB       0
LB                1.00 Hz
GB        0
PC                1.40
```

Title :
Run File : C:\Users\DG\Documents\SYNTHENOVA\Mode opératoire\SN0346-49 421-26\thiuram HPLC\HPLC ok\SN0424-L1.run
Method File : C:\star\startup1.mth
Sample ID : SN0424-L1

Injection Date: 21/05/2021 16:11   Calculation Date: 21/05/2021 16:31

Operator : dG   Detector Type: 0800 (1 Volt)
Workstation: HDD   Bus Address : 81
Instrument : Instrument #1   Sample Rate : 10.00 Hz
Channel : 2 = 2 1   Run Time : 20.002 min

Chart Speed = 1.08 cm/min   Attenuation = 4712   Zero Offset = 2%
Start Time = 0.000 min   End Time = 20.002 min   Min / Tick = 1.00

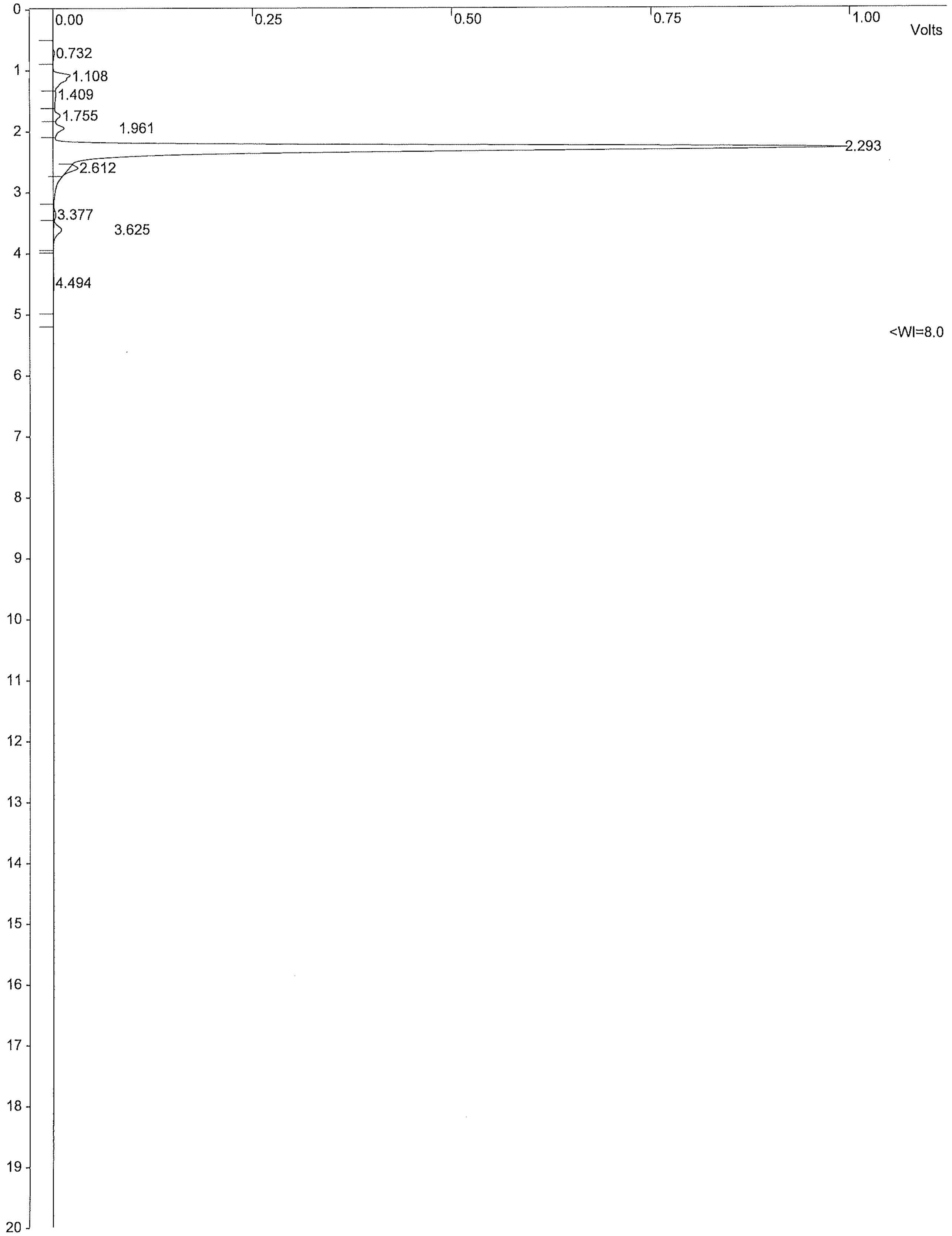

Print Date: Thu Jun 03 09:52:45 2021

Title :
Run File : C:\Users\DG\Documents\SYNTHENOVA\Mode opératoire\SN0346-49 421-26\thiuram HPLC\HPLC ok\SN0424-L1.run
Method File : C:\star\startup1.mth
Sample ID : SN0424-L1

Injection Date: 21/05/2021 16:11 Calculation Date: 21/05/2021 16:31

Operator : dG
Workstation: HDD
Instrument : Instrument #1
Channel : 2 = 2 1

Detector Type: 0800 (1 Volt)
Bus Address : 81
Sample Rate : 10.00 Hz
Run Time : 20.002 min

Run Mode : Analysis
Peak Measurement: Peak Area
Calculation Type: Percent

Peak No.	Peak Name	Result ()	Ret. Time (min)	Time Offset (min)	Area (counts)	Sep. Code	Width 1/2 (sec)	Status Codes
1		0.1945	0.732	0.000	16025	BV	6.9	
2		2.7035	1.108	0.000	222712	VV	9.3	
3		0.6027	1.409	0.000	49648	VV	0.0	
4		0.8249	1.755	0.000	67959	VV	6.8	
5		1.3568	1.961	0.000	111774	VV	6.8	
6		91.9821	2.293	0.000	7577512	VB	6.4	
7		0.6958	2.612	0.000	57322	TS	0.0	
8		0.2441	3.377	0.000	20110	TF	0.0	
9		1.1896	3.625	0.000	97998	TF	0.0	
10		0.2060	4.494	0.000	16973	TS	0.0	
	Totals:	100.0000		0.000	8238033			

Total Unidentified Counts : 8238033 counts

Detected Peaks: 10 Rejected Peaks: 0 Identified Peaks: 0

Multiplier: 1 Divisor: 1 Unidentified Peak Factor: 1

Baseline Offset: -43 microVolts LSB: 1 microVolts

Noise (used): 26 microVolts - monitored before this run

Manual injection

****

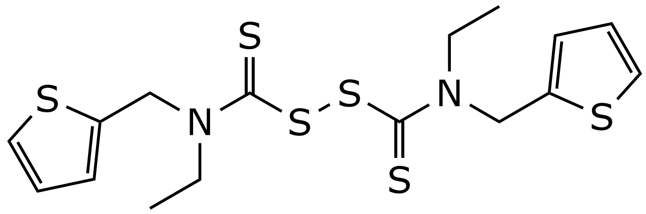

(**4h**)

Chemical Formula: $C_{16}H_{20}N_2S_6$
Exact Mass: 431.9951

SHIMADZU

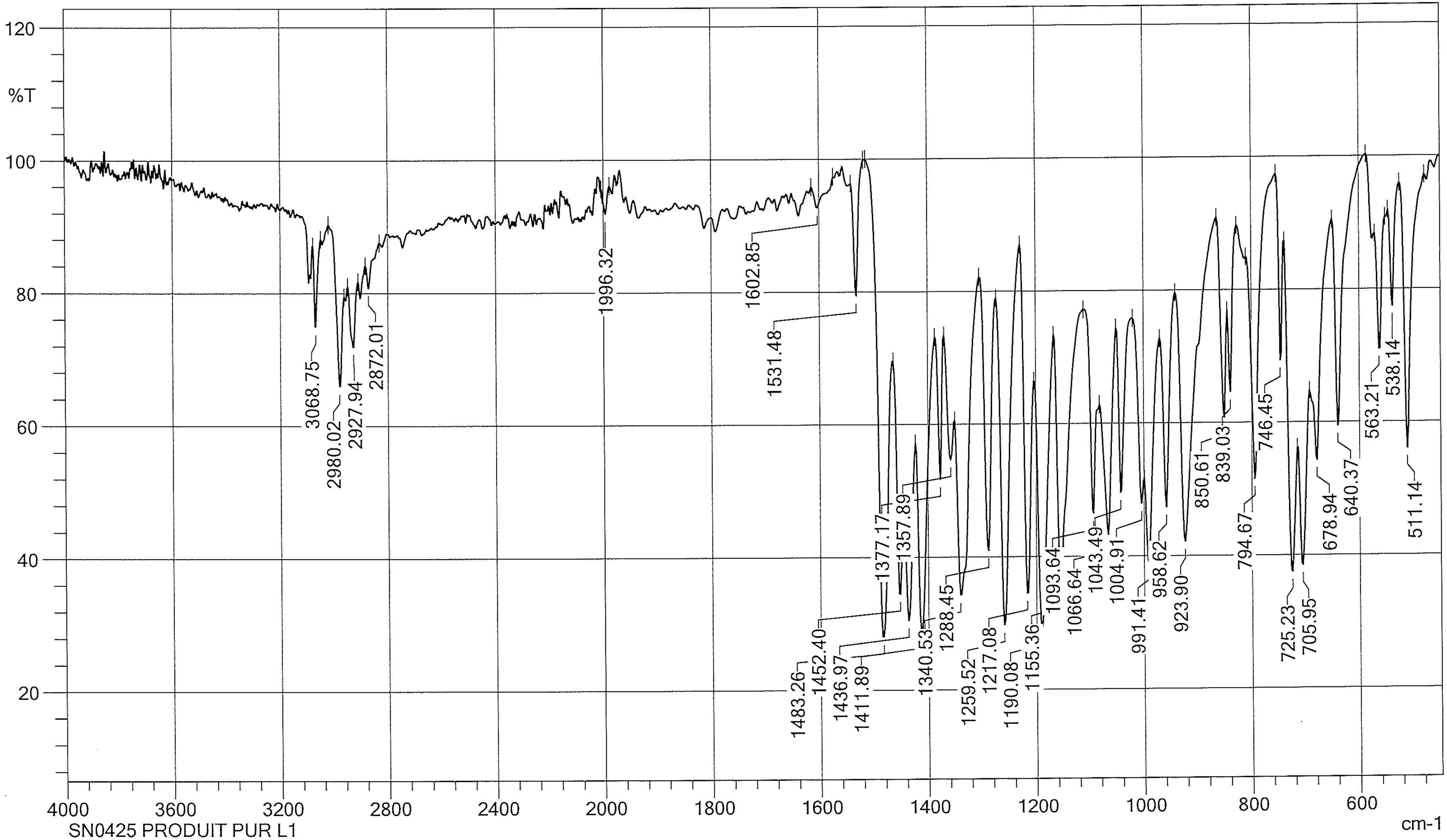

10/05/2021 16:05:18

SN0425 PRODUIT PUR L1

Elemental Composition Report

Single Mass Analysis

Tolerance = 5.0 PPM / DBE: min = 0.0, max = 14.0
Element prediction: Off
Number of isotope peaks used for i-FIT = 4

Monoisotopic Mass, Even Electron Ions
194 formula(e) evaluated with 1 results within limits (up to 50 best isotopic matches for each mass)
Elements Used:
C: 0-500 H: 0-1000 N: 0-5 S: 0-6

SN0425 2:17

01-Jun-2021
2106010 540 (3.282) Cm (537:543-(506:514+567:578))

1: TOF MS ES+
4.10e+004

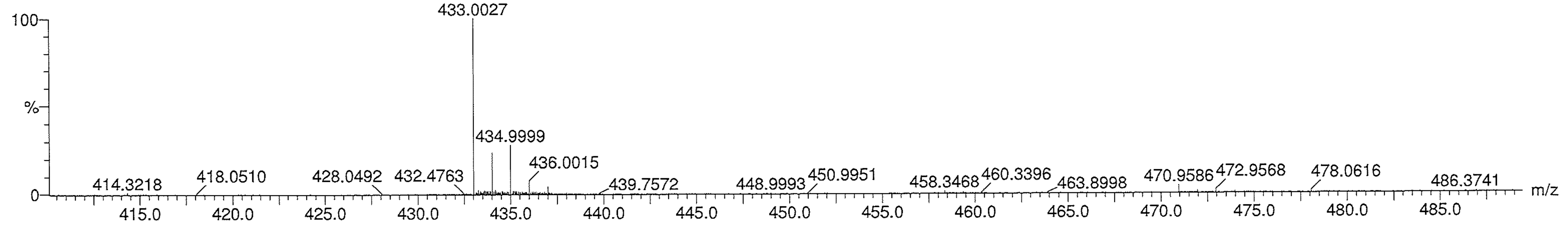

Minimum:				0.0			
Maximum:		5.0	5.0	14.0			
Mass	Calc. Mass	mDa	PPM	DBE	i-FIT	i-FIT (Norm)	Formula
433.0027	433.0029	-0.2	-0.5	7.5	714.7	0.0	C16 H21 N2 S6

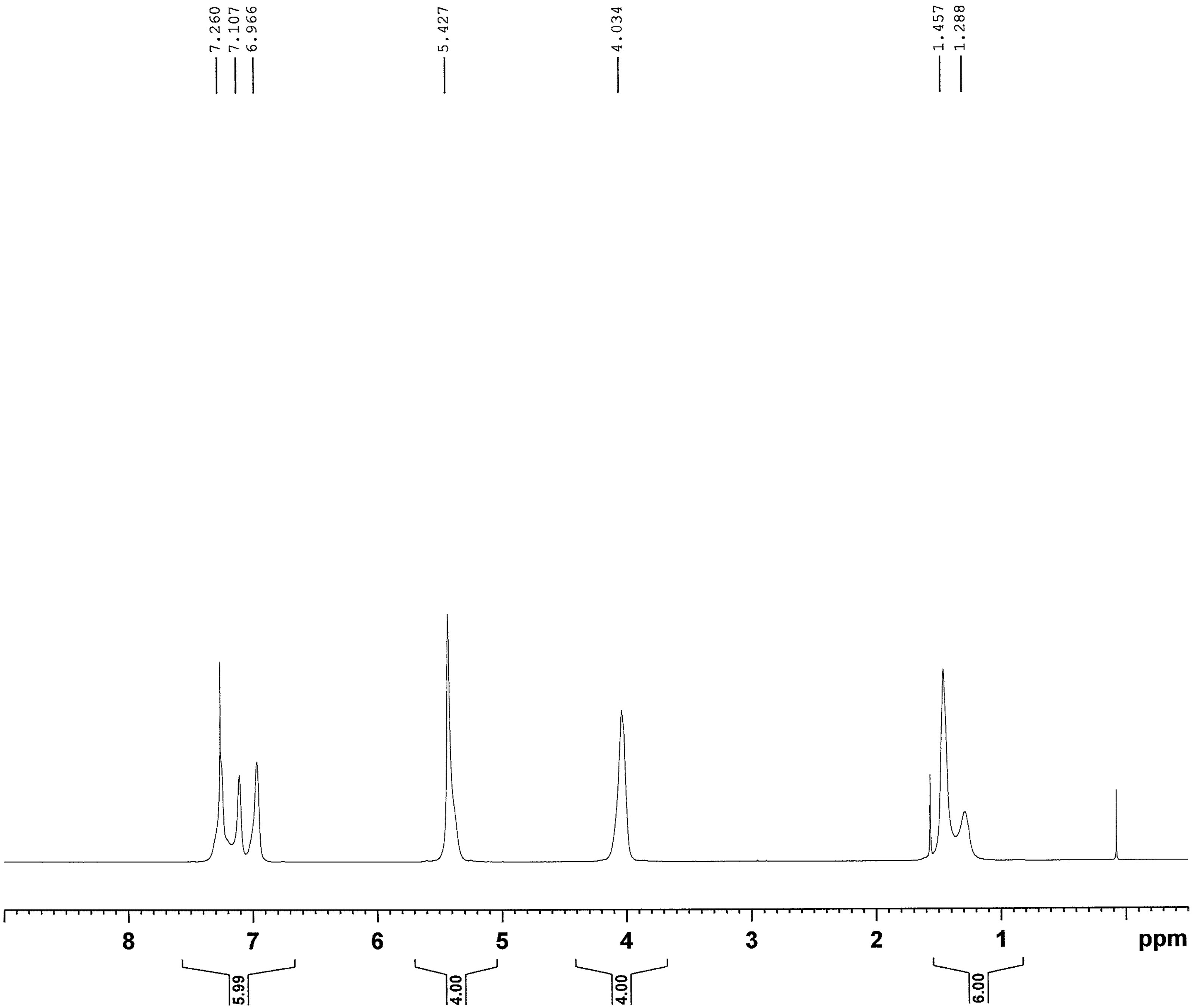

Current Data Parameters
NAME SN0425LOT1
EXPNO 10
PROCNO 1

F2 - Acquisition Parameters
Date_ 20210510
Time_ 14.43
INSTRUM spect
PROBHD 5 mm PABBO BB/
PULPROG zg30
TD 32768
SOLVENT CDCl3
NS 16
DS 2
SWH 7978.724 Hz
FIDRES 0.243491 Hz
AQ 2.0534613 sec
RG 69.13
DW 62.667 usec
DE 6.50 usec
TE 296.2 K
D1 1.00000000 sec
TD0 1

======== CHANNEL f1 ========
SFO1 399.7524686 MHz
NUC1 1H
P1 9.00 usec
PLW1 19.20000076 W

F2 - Processing parameters
SI 65536
SF 399.7500097 MHz
WDW EM
SSB 0
LB 0.30 Hz
GB 0
PC 1.00

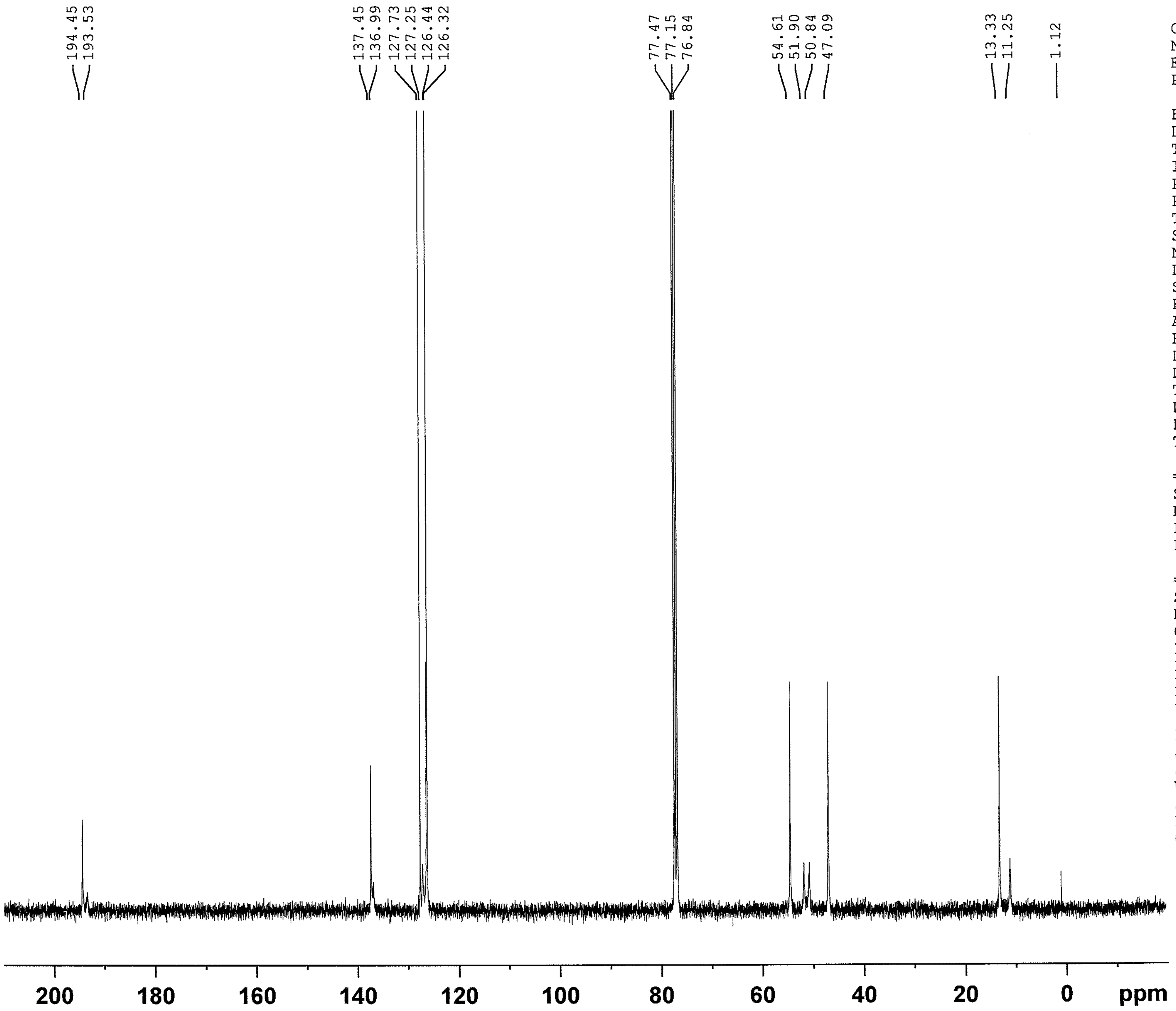

Current Data Parameters
NAME SN0425-FINAL
EXPNO 10
PROCNO 1

F2 - Acquisition Parameters
Date_ 20210511
Time 5.00
INSTRUM spect
PROBHD 5 mm PABBO BB/
PULPROG zgpg30
TD 65536
SOLVENT CDCl3
NS 2048
DS 4
SWH 24038.461 Hz
FIDRES 0.366798 Hz
AQ 1.3631488 sec
RG 199.3
DW 20.800 usec
DE 6.50 usec
TE 295.0 K
D1 2.00000000 sec
D11 0.03000000 sec
TD0 1

======== CHANNEL f1 ========
SFO1 100.5272687 MHz
NUC1 13C
P1 8.60 usec
PLW1 87.00000000 W

======== CHANNEL f2 ========
SFO2 399.7515990 MHz
NUC2 1H
CPDPRG[2 waltz16
PCPD2 90.00 usec
PLW2 19.20000076 W
PLW12 0.20000000 W
PLW13 0.15552001 W

F2 - Processing parameters
SI 32768
SF 100.5172114 MHz
WDW EM
SSB 0
LB 1.00 Hz
GB 0
PC 1.40

Title :
Run File : C:\Users\DG\Documents\SYNTHENOVA\Mode opératoire\SN0346-49 421-26\thiuram HPLC\HPLC ok\SN0425-L1.run
Method File : C:\star\startup1.mth
Sample ID : SN0425-L1

Injection Date: 12/05/2021 16:53   Calculation Date: 12/05/2021 17:13

Operator : dG   Detector Type: 0800 (1 Volt)
Workstation: HDD   Bus Address : 81
Instrument : Instrument #1   Sample Rate : 10.00 Hz
Channel : 2 = 2 1   Run Time : 20.002 min

Chart Speed = 1.08 cm/min   Attenuation = 2647   Zero Offset = 2%
Start Time = 0.000 min   End Time = 20.002 min   Min / Tick = 1.00

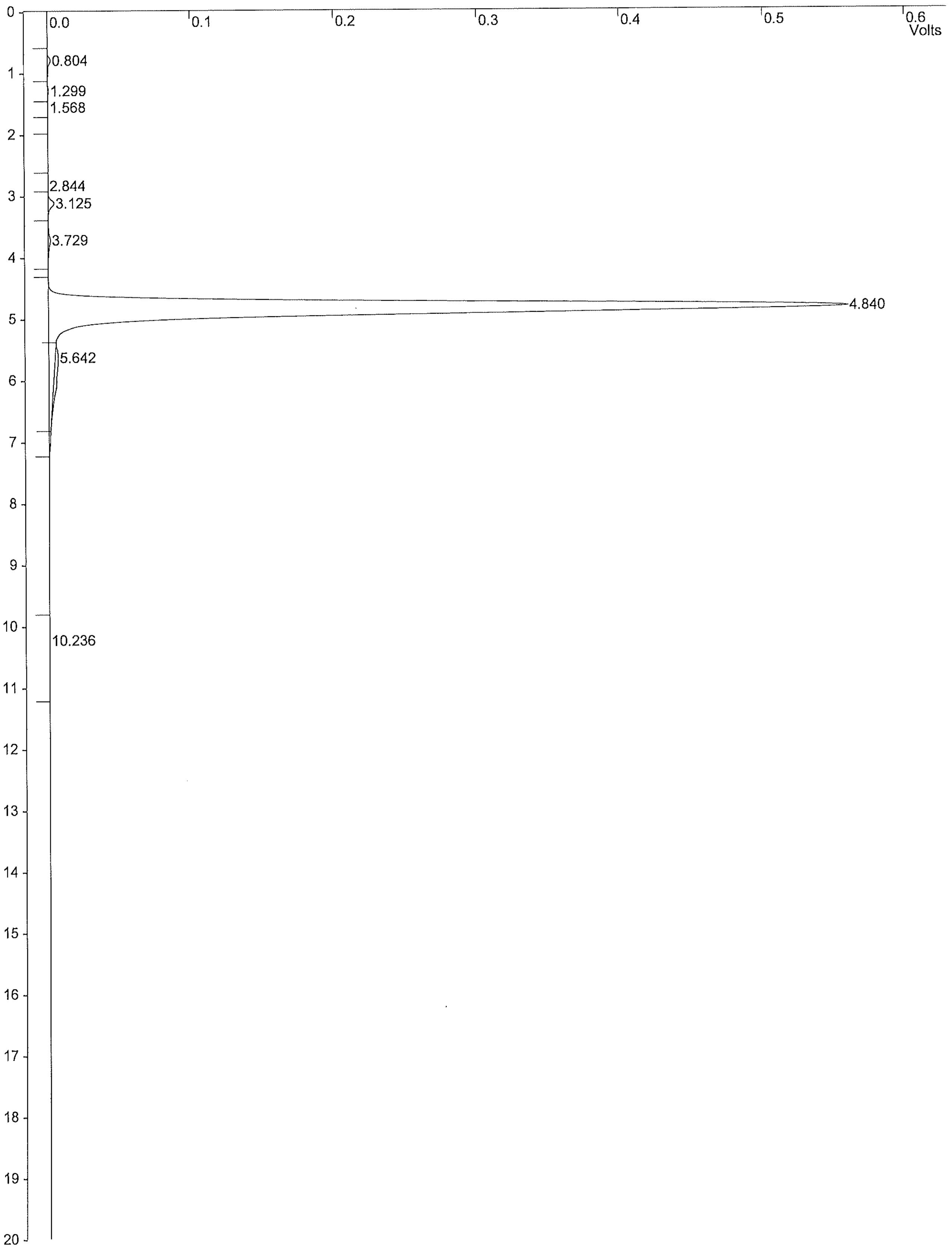

Title :
Run File : C:\Users\DG\Documents\SYNTHENOVA\Mode opératoire\SN0346-49 421-26\thiuram HPLC\HPLC ok\SN0425-L1.run
Method File : C:\star\startup1.mth
Sample ID : SN0425-L1

Injection Date: 12/05/2021 16:53 Calculation Date: 12/05/2021 17:13

Operator : dG
Workstation: HDD
Instrument : Instrument #1
Channel : 2 = 2 1

Detector Type: 0800 (1 Volt)
Bus Address : 81
Sample Rate : 10.00 Hz
Run Time : 20.002 min

Run Mode : Analysis
Peak Measurement: Peak Area
Calculation Type: Percent

Peak No.	Peak Name	Result ()	Ret. Time (min)	Time Offset (min)	Area (counts)	Sep. Code	Width 1/2 (sec)	Status Codes
1		0.2273	0.804	0.000	18378	BP	7.0	
2		0.0788	1.299	0.000	6368	PV	8.3	
3		0.0339	1.568	0.000	2744	VV	8.8	
4		0.0160	2.844	0.000	1292	BV	10.3	
5		0.4527	3.125	0.000	36597	VV	7.7	
6		0.2954	3.729	0.000	23887	VB	11.1	
7		97.5228	4.840	0.000	7884557	BB	12.2	
8		1.2970	5.642	0.000	104861	TS	0.0	
9		0.0761	10.236	0.000	6154	BB	0.0	
	Totals:	100.0000		0.000	8084838			

Total Unidentified Counts : 8084837 counts

Detected Peaks: 10 Rejected Peaks: 1 Identified Peaks: 0

Multiplier: 1 Divisor: 1 Unidentified Peak Factor: 1

Baseline Offset: -33 microVolts LSB: 1 microVolts

Noise (used): 28 microVolts - monitored before this run

Manual injection

****

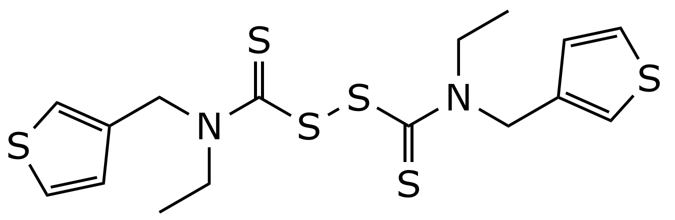

(**4i**)

Chemical Formula: $C_{16}H_{20}N_2S_6$
Exact Mass: 431.9951

SHIMADZU

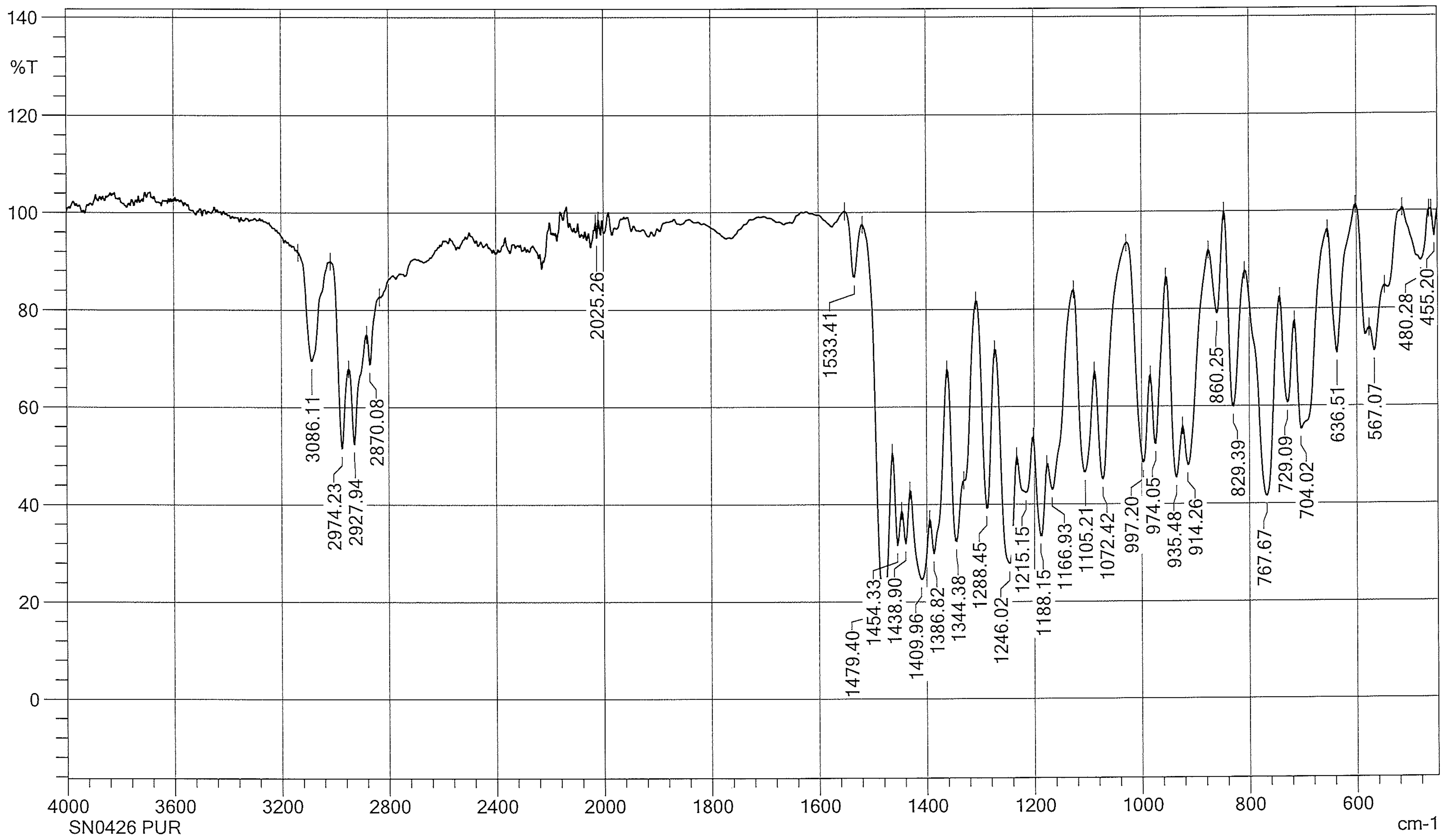

SN0426 PUR

05/05/2021 11:36:40

Elemental Composition Report

Single Mass Analysis

Tolerance = 5.0 PPM / DBE: min = 0.0, max = 14.0
Element prediction: Off
Number of isotope peaks used for i-FIT = 4

Monoisotopic Mass, Even Electron Ions
194 formula(e) evaluated with 1 results within limits (up to 50 best isotopic matches for each mass)
Elements Used:
C: 0-500 H: 0-1000 N: 0-5 S: 0-6

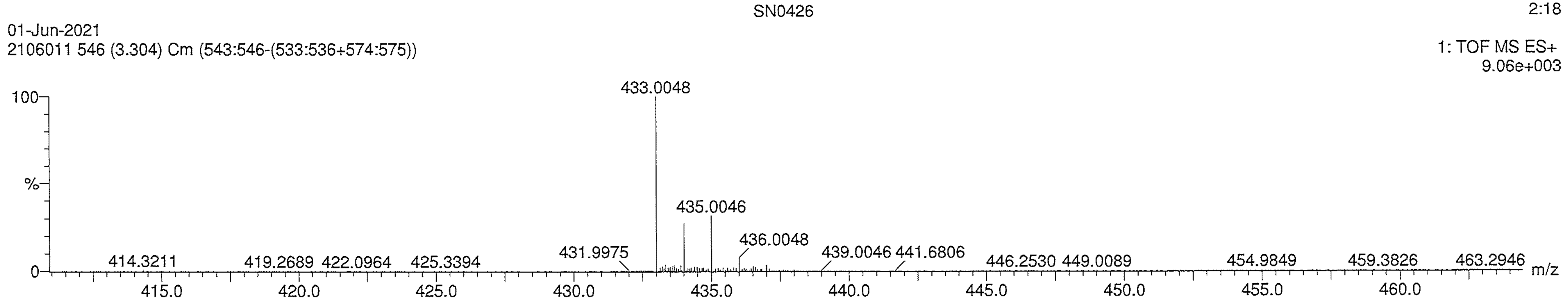

Minimum:				0.0			
Maximum:		5.0	5.0	14.0			
Mass	Calc. Mass	mDa	PPM	DBE	i-FIT	i-FIT (Norm)	Formula
433.0048	433.0029	1.9	4.4	7.5	432.0	0.0	C16 H21 N2 S6

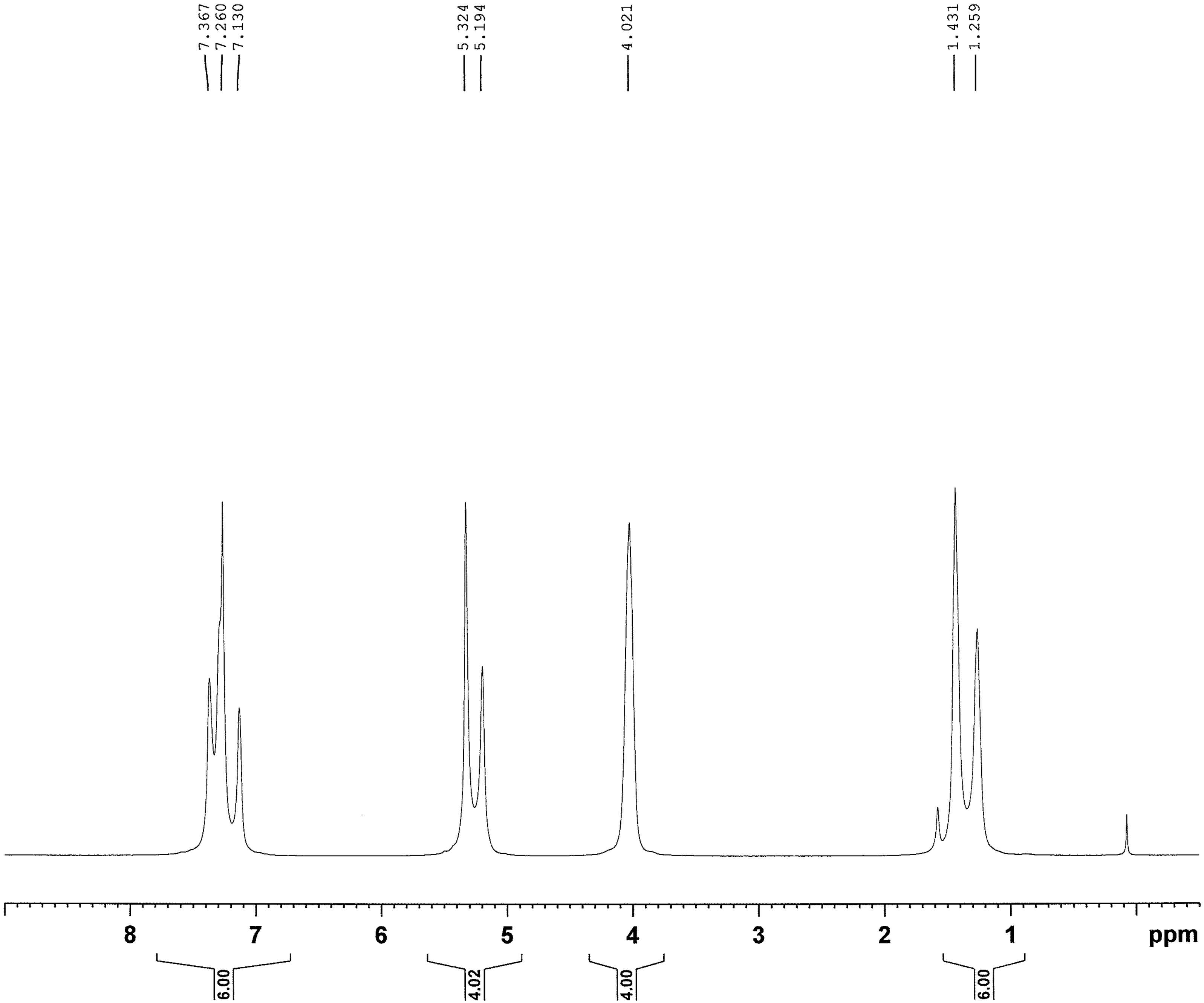


```
Current Data Parameters
NAME          SN0426-L1
EXPNO                10
PROCNO                1

F2 - Acquisition Parameters
Date_          20210429
Time              14.51
INSTRUM           spect
PROBHD   5 mm PABBO BB/
PULPROG            zg30
TD                32768
SOLVENT           CDCl3
NS                   16
DS                    2
SWH            7978.724 Hz
FIDRES         0.243491 Hz
AQ            2.0534613 sec
RG                61.21
DW               62.667 usec
DE                 6.50 usec
TE                296.2 K
D1           2.00000000 sec
TD0                   1

======== CHANNEL f1 ========
SFO1        399.7524686 MHz
NUC1                 1H
P1                 9.00 usec
PLW1        19.20000076 W

F2 - Processing parameters
SI                65536
SF          399.7500100 MHz
WDW                  EM
SSB      0
LB                 0.30 Hz
GB       0
PC                 1.00
```

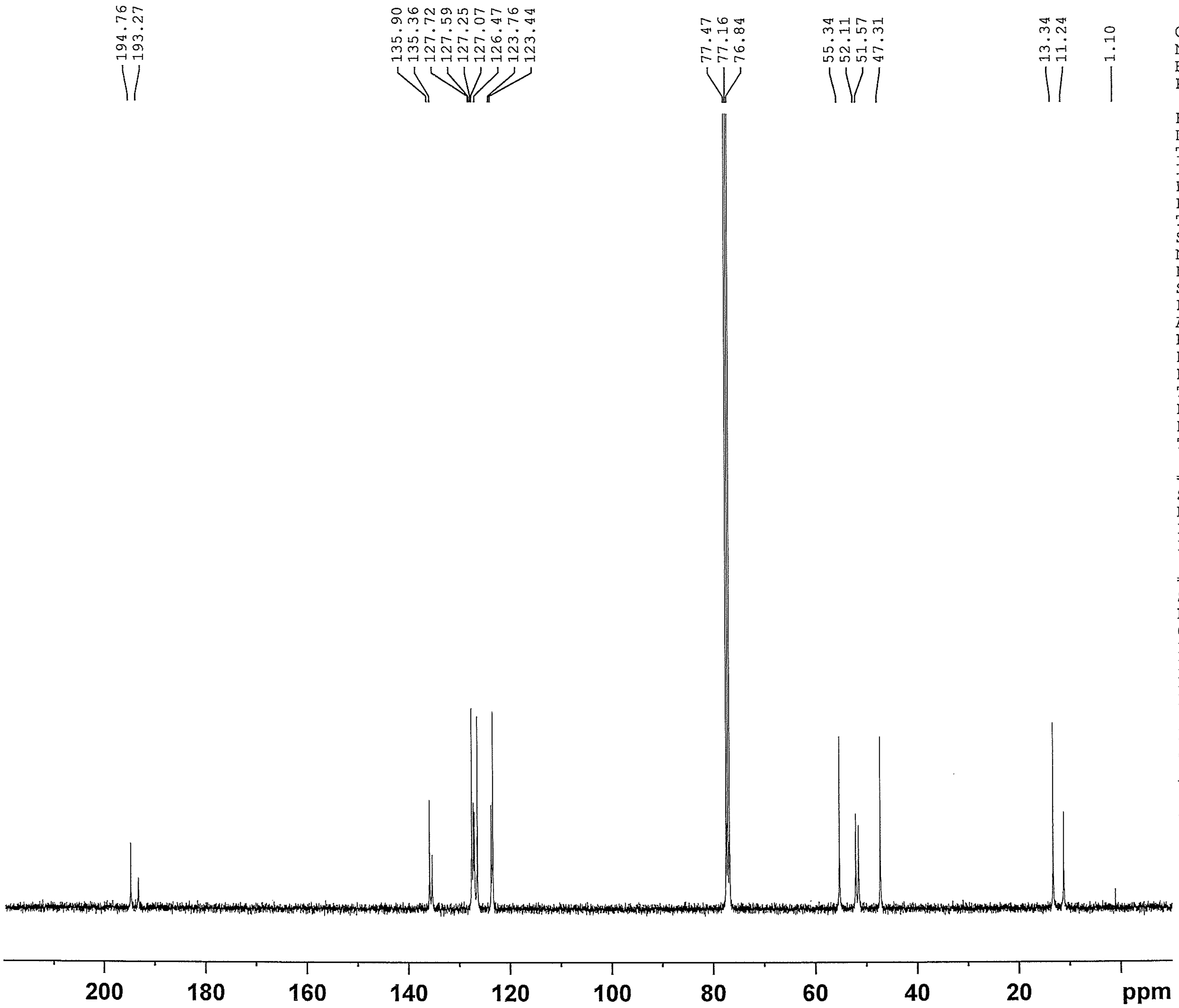

Current Data Parameters
NAME SN0426L1
EXPNO 10
PROCNO 1

F2 - Acquisition Parameters
Date_ 20210501
Time 22.35
INSTRUM spect
PROBHD 5 mm PABBO BB/
PULPROG zgpg30
TD 65536
SOLVENT CDCl3
NS 2048
DS 4
SWH 24038.461 Hz
FIDRES 0.366798 Hz
AQ 1.3631488 sec
RG 199.3
DW 20.800 usec
DE 6.50 usec
TE 295.0 K
D1 2.00000000 sec
D11 0.03000000 sec
TD0 1

======== CHANNEL f1 ========
SFO1 100.5272687 MHz
NUC1 13C
P1 8.60 usec
PLW1 87.00000000 W

======== CHANNEL f2 ========
SFO2 399.7515990 MHz
NUC2 1H
CPDPRG[2 waltz16
PCPD2 90.00 usec
PLW2 19.20000076 W
PLW12 0.20000000 W
PLW13 0.15552001 W

F2 - Processing parameters
SI 32768
SF 100.5172130 MHz
WDW EM
SSB 0
LB 1.00 Hz
GB 0
PC 1.40

Title ' :
Run File : C:\Users\DG\Documents\SYNTHENOVA\Mode opératoire\SN0346-49 421-26\thiuram HPLC\HPLC ok\SN0426-L1.run
Method File : C:\star\startup1.mth
Sample ID : SN0426-L1

Injection Date: 12/05/2021 15:31   Calculation Date: 12/05/2021 15:51

Operator : DG   Detector Type: 0800 (1 Volt)
Workstation: HDD   Bus Address : 81
Instrument : Instrument #1   Sample Rate : 10.00 Hz
Channel : 2 = 2 1   Run Time : 20.002 min

Chart Speed = 1.08 cm/min   Attenuation = 3539   Zero Offset = 2%
Start Time = 0.000 min   End Time = 20.002 min   Min / Tick = 1.00

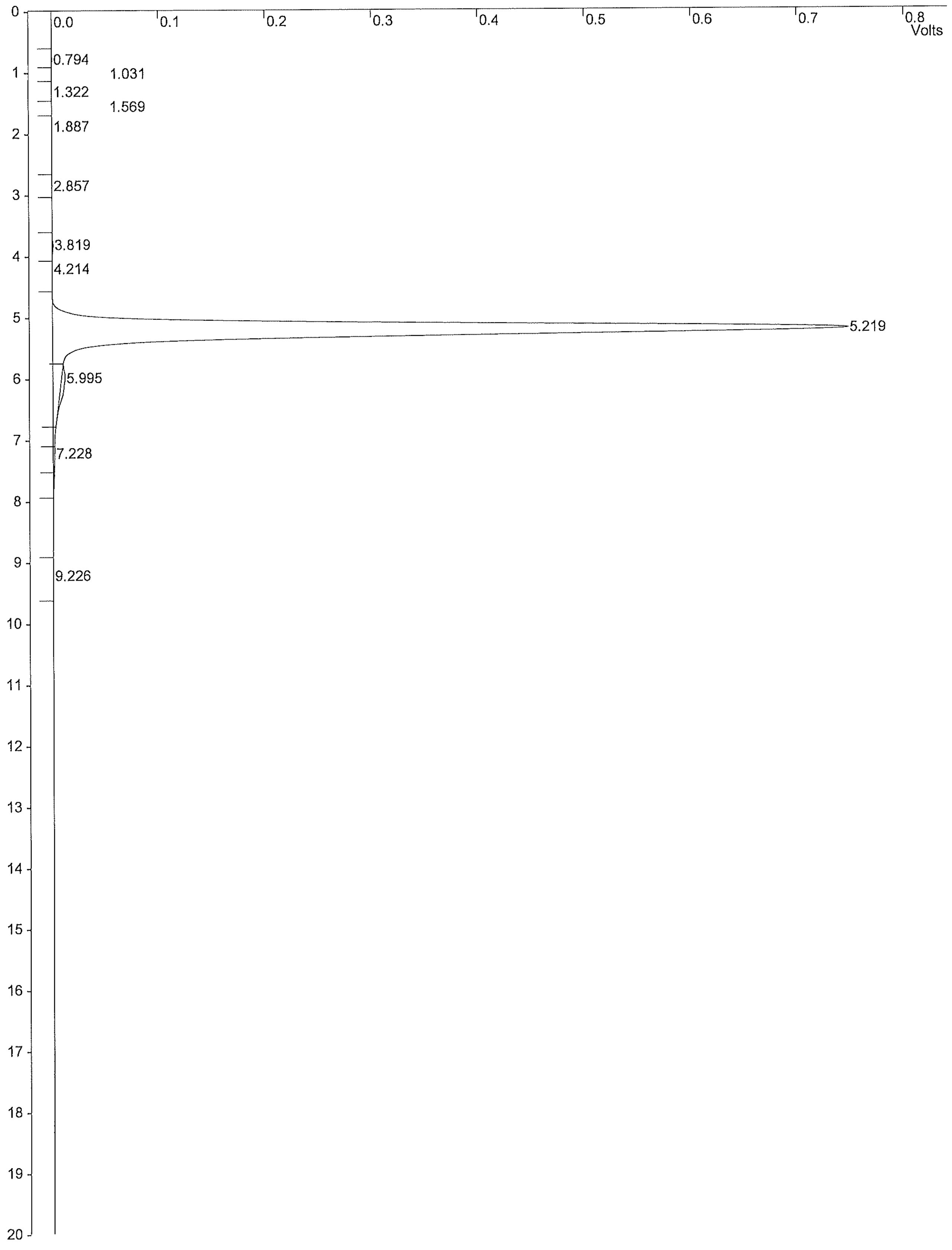

Title :
Run File : C:\Users\DG\Documents\SYNTHENOVA\Mode opératoire\SN0346-49 421-26\thiuram HPLC\HPLC ok\SN0426-L1.run
Method File : C:\star\startup1.mth
Sample ID : SN0426-L1

Injection Date: 12/05/2021 15:31 Calculation Date: 12/05/2021 15:51

Operator : DG
Workstation: HDD
Instrument : Instrument #1
Channel : 2 = 2 1

Detector Type: 0800 (1 Volt)
Bus Address : 81
Sample Rate : 10.00 Hz
Run Time : 20.002 min

Run Mode : Analysis
Peak Measurement: Peak Area
Calculation Type: Percent

Peak No.	Peak Name	Result ()	Ret. Time (min)	Time Offset (min)	Area (counts)	Sep. Code	Width 1/2 (sec)	Status Codes
1		0.0356	0.794	0.000	3907	BV	7.3	
2		0.0138	1.031	0.000	1518	VP	7.8	
3		0.0194	1.322	0.000	2129	PV	10.8	
4		0.0145	1.569	0.000	1591	VV	7.3	
5		0.0451	1.887	0.000	4947	VP	6.8	
6		0.0112	2.857	0.000	1226	PB	8.3	
7		0.1067	3.819	0.000	11693	BV	10.9	
8		0.0271	4.214	0.000	2969	VP	11.6	
9		98.7029	5.219	0.000	10818486	PB	12.2	
10		0.9584	5.995	0.000	105049	TS	0.0	
11		0.0240	7.228	0.000	2631	TS	0.0	
12		0.0411	9.226	0.000	4510	BB	16.1	
	Totals:	99.9998		0.000	10960656			

Total Unidentified Counts : 10960656 counts

Detected Peaks: 12 Rejected Peaks: 0 Identified Peaks: 0

Multiplier: 1 Divisor: 1 Unidentified Peak Factor: 1

Baseline Offset: -37 microVolts LSB: 1 microVolts

Noise (used): 31 microVolts - monitored before this run

Manual injection

****
